# Accuracy of Large Language Models When Answering Clinical Research Questions: Systematic Review and Network Meta-Analysis

**DOI:** 10.2196/64486

**Published:** 2025-04-30

**Authors:** Ling Wang, Jinglin Li, Boyang Zhuang, Shasha Huang, Meilin Fang, Cunze Wang, Wen Li, Mohan Zhang, Shurong Gong

**Affiliations:** 1 Fuzhou University Affiliated Provincial Hospital Shengli Clinical Medical College Fujian Medical University Fuzhou China; 2 School of Pharmacy Fujian Medical University Fuzhou China; 3 Fujian Center For Drug Evaluation and Monitoring Fuzhou China; 4 School of Pharmacy Fujian University of Traditional Chinese Medicine Fuzhou China; 5 The Third Department of Critical Care Medicine Fuzhou University Affiliated Provincial Hospital Shengli Clinical Medical College, Fujian Medical University Fuzhou, Fujian China

**Keywords:** large language models, LLM, clinical research questions, accuracy, network meta-analysis, PRISMA

## Abstract

**Background:**

Large language models (LLMs) have flourished and gradually become an important research and application direction in the medical field. However, due to the high degree of specialization, complexity, and specificity of medicine, which results in extremely high accuracy requirements, controversy remains about whether LLMs can be used in the medical field. More studies have evaluated the performance of various types of LLMs in medicine, but the conclusions are inconsistent.

**Objective:**

This study uses a network meta-analysis (NMA) to assess the accuracy of LLMs when answering clinical research questions to provide high-level evidence-based evidence for its future development and application in the medical field.

**Methods:**

In this systematic review and NMA, we searched PubMed, Embase, Web of Science, and Scopus from inception until October 14, 2024. Studies on the accuracy of LLMs when answering clinical research questions were included and screened by reading published reports. The systematic review and NMA were conducted to compare the accuracy of different LLMs when answering clinical research questions, including objective questions, open-ended questions, top 1 diagnosis, top 3 diagnosis, top 5 diagnosis, and triage and classification. The NMA was performed using Bayesian frequency theory methods. Indirect intercomparisons between programs were performed using a grading scale. A larger surface under the cumulative ranking curve (SUCRA) value indicates a higher ranking of the corresponding LLM accuracy.

**Results:**

The systematic review and NMA examined 168 articles encompassing 35,896 questions and 3063 clinical cases. Of the 168 studies, 40 (23.8%) were considered to have a low risk of bias, 128 (76.2%) had a moderate risk, and none were rated as having a high risk. ChatGPT-4o (SUCRA=0.9207) demonstrated strong performance in terms of accuracy for objective questions, followed by Aeyeconsult (SUCRA=0.9187) and ChatGPT-4 (SUCRA=0.8087). ChatGPT-4 (SUCRA=0.8708) excelled at answering open-ended questions. In terms of accuracy for top 1 diagnosis and top 3 diagnosis of clinical cases, human experts (SUCRA=0.9001 and SUCRA=0.7126, respectively) ranked the highest, while Claude 3 Opus (SUCRA=0.9672) performed well at the top 5 diagnosis. Gemini (SUCRA=0.9649) had the highest rated SUCRA value for accuracy in the area of triage and classification.

**Conclusions:**

Our study indicates that ChatGPT-4o has an advantage when answering objective questions. For open-ended questions, ChatGPT-4 may be more credible. Humans are more accurate at the top 1 diagnosis and top 3 diagnosis. Claude 3 Opus performs better at the top 5 diagnosis, while for triage and classification, Gemini is more advantageous. This analysis offers valuable insights for clinicians and medical practitioners, empowering them to effectively leverage LLMs for improved decision-making in learning, diagnosis, and management of various clinical scenarios.

**Trial Registration:**

PROSPERO CRD42024558245; https://www.crd.york.ac.uk/PROSPERO/view/CRD42024558245

## Introduction

Recent research has demonstrated the considerable success of large language models (LLMs) in a multitude of natural language tasks, including automatic summarization (the generation of a condensed version of a passage of text), machine translation (the automatic translation of text from one language to another), and question-and-answer systems (the construction of a system to automatically answer questions based on a passage of text) [1]. In this context, with the development of big biomedical data and artificial intelligence, the emergence of flexible natural language processing models such as ChatGPT provides a number of new possibilities for health care and biomedical research and has the potential to be a turning point in the field [2-4].

Although LLMs have shown great potential in the medical field, medicine is a demanding field, it is associated with life, and its complexity as well as specificity mean that any application must meet extremely high standards of accuracy. Controversy remains about whether LLMs can be applied to the medical field. Mu and He [5] reviewed the potential applications and challenges of ChatGPT in health care, noting that a lack of understanding of medical knowledge and specialized medical backgrounds hinder the ability of ChatGPT to delve into the complexity of medical concepts and terminology. Consequently, the capacity of ChatGPT to address specific medical queries, diagnose ailments, or furnish precise medical recommendations is restricted. Another study noted that the role of LLMs in health care may be limited by the presence of bias in training materials, their tendency to “hallucinate,” and ethical and legal considerations when LLMs provide inaccurate advice that leads to patient harm, as well as patient privacy issues [6].

Given the controversy over the application of LLMs in medicine and the continuous emergence and versioning of LLMs, more research has been devoted to evaluating the performance of various LLMs in medicine to provide stronger evidence. In addition to ChatGPT developed by OpenAI, the performance of many other LLMs such as Microsoft (eg, Copilot [7]), Google (eg, Gemini [8]), and Meta (eg, LLaMA [9]) in the medical domain has also been compared. Many aspects of assessment have been included, such as medical exams [10], case text diagnosis [11], and disease classification or grading [12].

Unfortunately, there are differences in the performance of different LLMs in different studies. For example, in a study by Vaishya et al [13] that explored the performance of ChatGPT-3.5, ChatGPT-4, and Google Bard when answering 120 multiple-choice questions, the results showed that Google Bard had 100% accuracy and was significantly more accurate than both ChatGPT-3.5 and ChatGPT-4 (*P*<.001). Another study showed that ChatGPT-4 was more accurate than Google Bard (83% vs 76%) [14]. At present, most related research is limited to a single type of LLM [15,16] or a specific domain area [17,18], and there is no high-level evidence comparing the accuracy rankings of different LLMs when responding to clinical research questions.

Therefore, this study aimed to compare the accuracy of different LLMs when answering clinical research questions, including objective questions, open-ended questions, top 1 diagnosis, top 3 diagnosis, top 5 diagnosis, and triage and classification. This study aimed to provide high-level evidence-based support for future clinical applications, enabling clinical workers to better use LLMs to make more accurate and informed decisions for future learning, diagnosis, and different clinical scenarios.

## Methods

### Network Meta-Analysis

The network meta-analysis (NMA) was based on the PRISMA (Preferred Reporting Items for Systematic Reviews and Meta-Analyses) reporting guidelines. The PRISMA checklist is shown in Multimedia Appendix 1. The Bayesian approach permits the indirect comparison of performance between a range of LLMs that were not explicitly articulated throughout the experiment. The study protocol was defined and registered in the PROSPERO database prior to the commencement of the study.

### Search Strategy and Selection Criteria

A computer search of the PubMed, Embase, Web of Science, and Scopus databases was conducted to identify relevant studies on the accuracy of different LLMs when answering questions in the medical field. The last search was updated to October 14, 2024, to identify studies published since the first search, with no restrictions on the type of study. When the results of a study were reported in multiple publications, we included the study with the richest and most recent findings. We also searched the list of literature on LLMs in medicine-related systematic reviews and manually searched the references included in the reviews for additional access to relevant literature. The search subject terms were “LLM,” “generative AI,” “open AI,” “Large language model,” “ChatGPT-3.5,” “ChatGPT-4,” “Google Bard,” and “Bing,” without any language restriction. The complete search strategies for all databases are shown in Multimedia Appendix 2.

A combination of EndNote X9 deduplication and manual deduplication was used to screen the literature in accordance with the developed inclusion criteria. The results of the literature searches conducted in different databases were then combined to create a new information database, which could be downloaded in full text. Independent review and assessment of the titles, abstracts, and full texts of the relevant literature were undertaken by 4 authors (LW, JL, BZ, and SH). The review encompassed studies using disparate LLMs systems to respond to medical queries. Letters, conference abstracts, editorials, reviews, and expert opinions for which no information was available were excluded from the review. In addition, the following studies were excluded: those that evaluated the performance of only 1 LLM; those that assessed the performance of 2 or more LLMs without specifying the LLM versions used (eg, the article only mentioned evaluating ChatGPT without mentioning ChatGPT-3.5, ChatGPT-4, or other versions), with the updated versions and timelines of various LLMs so far shown in Multimedia Appendix 3; those that assessed the performance of 2 or more LLMs but did not provide data isolating their accuracy when answering different types of questions; and the questions included in the study contained images. In addition, to reduce bias, we excluded research on accessing LLMs through an application programming interface (API).

### Assessment of Results

The primary outcomes were the accuracy of LLMs when answering medical questions. These included objective questions, open-ended questions, top 1 diagnosis, top 3 diagnosis, top 5 diagnosis, and triage and classification accuracy. Objective questions are exam questions with a clear, quantifiable answer that is usually predetermined, unique, or with a limited number of options. Open-ended questions are a type of question that does not have a fixed answer nor standardized answer. Diagnosis and triage and classification are open-ended questions, but most diagnostic questions end with “What is the most probable diagnosis?” whereas triage and classification questions end with “How would you classify this disease?” Corresponding examples are shown in Multimedia Appendix 4.

Accuracy for objective questions was calculated as the number of correctly answered questions divided by the total number of questions. For diagnosis and classification, accuracy was defined as the number of cases correctly diagnosed or triaged divided by the total number of cases. Specifically for open-ended questions, accuracy was determined based on the number of questions rated “good” or “accurate” on the accuracy scale divided by the total number of questions.

### Data Extraction

The 4 researchers jointly extracted and verified the following data: (1) basic information about the included studies, such as study title and first author; (2) baseline characteristics and interventions of the study population; (3) key elements evaluated for risk of bias; and (4) outcome indicators and relevant outcome measure data. Our study involved extracting raw data from each study. In cases of disagreement, these were resolved through discussion and consultation with a third party.

### Quality Assessment

Because they were cross-sectional studies, the quality of the included studies was evaluated using the Newcastle-Ottawa Scale [19]. The quality assessment was conducted by 3 independent researchers (LW, JL, and BZ), with a fourth researcher (SH) resolving any disagreements. A low overall risk of bias was determined when the Newcastle-Ottawa Scale score ranged from 7 to 9, moderate risk was determined when the score was between 4 and 6, and high risk was determined when the score was 0 to 3.

### Statistical Analyses

Statistical analyses were performed using Stata 18.0 and R (version 4.3.1), with the odds ratio (OR) as the analytical statistic. Accuracy was assessed using 95% CIs and the credible interval. NMA analyses were performed on different types of LLMs.

The confidence of the NMA results estimates was assessed according to the Confidence in Network Meta-Analysis (CINEMA) methodology, which is broadly based on the Grading of Recommendations Assessment, Development, and Evaluation (GRADE). An NMA was conducted within a Bayesian framework using Markov chain Monte Carlo methods and was computed using the BUGSnet and GeMTC packages in R (V.4.3.1) software. A network graph was constructed for each LLM included in the experiment in order to facilitate a comparison of the performance of multiple LLMs. The consistency between direct and indirect evidence was evaluated using a node-splitting method when there was a closed loop. If the *P* value between the direct, indirect, and network comparisons of the 2 interventions was >.05, we concluded that there was no statistical difference and consistency was good. The convergence of the network models derived from the Markov chain Monte Carlo simulations was assessed using trace and density plots. We used noninformative priors for all parameters and assumed common heterogeneity. Furthermore, for all LLMs, we determined the ranking probabilities, which were articulated as the surface under the cumulative ranking curve (SUCRA). Higher SUCRA values suggest superior accuracy in model ranking.

## Results

### Literature Search and Selection

**Figure 1:**
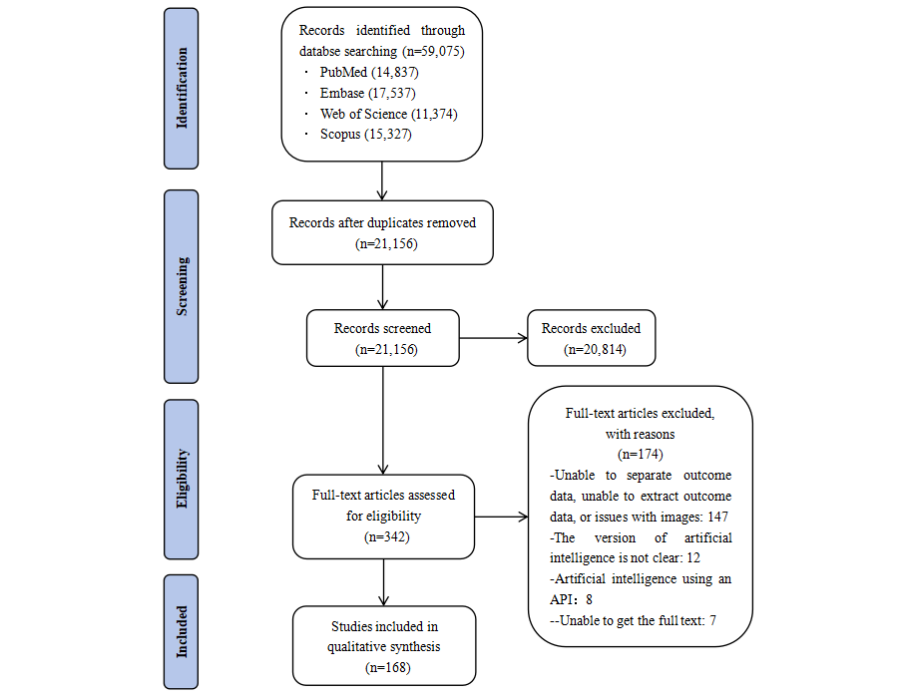
Literature screening flowchart. API: application programming interface.

A bibliographic search yielded 59,075 citations, of which 21,156 studies were identified as potential conditions based on abstract screening and retrieved for full text evaluation. Manual reading of the titles and abstracts of the remaining literature excluded 20,814 papers whose topics and interventions did not match the inclusion criteria for this study. Further reading of the full texts excluded the following: 174 articles that could not be separated nor extracted from the ending; 147 articles in which we were unable to separate outcome data, unable to extract outcome data, or detected issues related to images; 12 articles with unclear versions of the LLMs; and 8 articles that used an API to access LLMs. In addition, the full text of 7 articles was not available, resulting in the final inclusion of 168 articles from the literature. The literature screening process is shown in Figure 1.

### Basic Characteristics of the Incorporated Literature

To assess the accuracy of different LLMs when answering medical questions, a total of 168 studies underwent a screening process to determine their suitability for inclusion. A total of 35,896 questions and 3063 clinical cases were included in the study. The basic information of the 168 studies is presented in Multimedia Appendix 5.

### Quality Assessment of the Included Studies

In the quality assessment, 40 (40/168, 23.8%) studies were assessed as having a low overall risk of bias, while 128 (128/168, 76.2%) had a moderate overall risk of bias. No studies were identified as having a high overall risk of bias. The detailed quality assessment results for each study can be found in Multimedia Appendix 6.

### Network Meta-Analysis

#### Objective Questions

**Figure 2:**
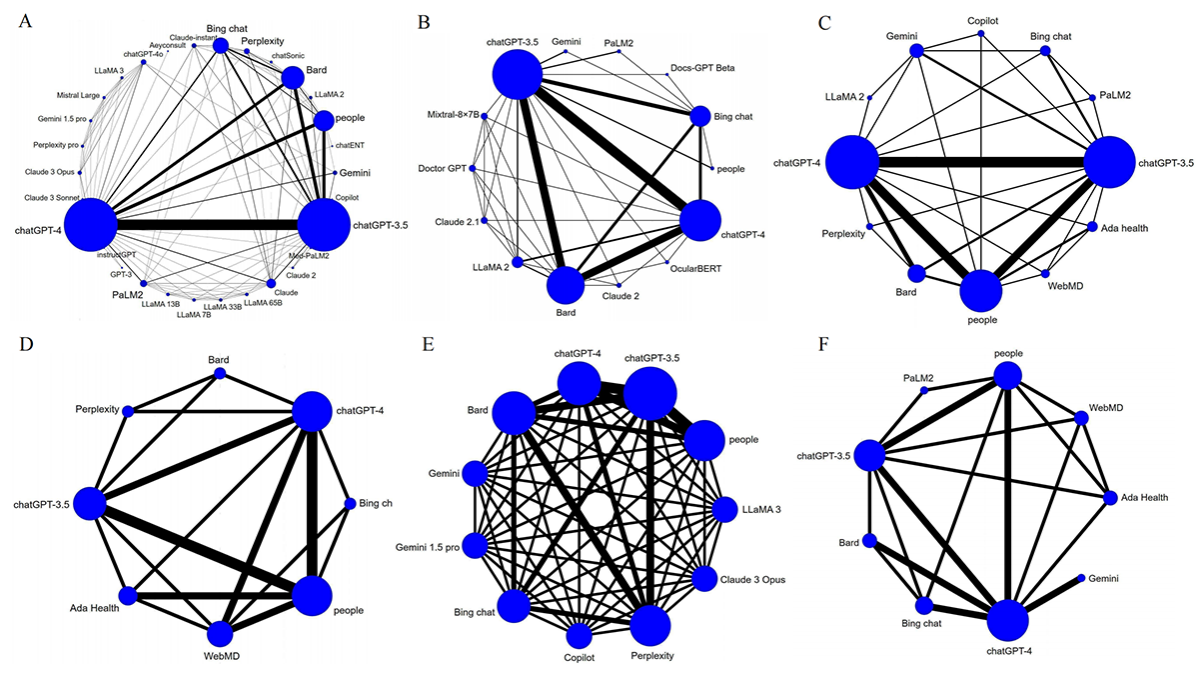
Comparison network diagram of different outcomes, where larger nodes indicate more questions and thicker line segments indicate more questions between 2 types of large language models (LLMs) when answering (A) objective questions, (B) open-ended questions, (C) a top 1 diagnosis, (D) a top 3 diagnosis, (E) a top 5 diagnosis, and (F) triage and classification questions.

**Figure 3:**
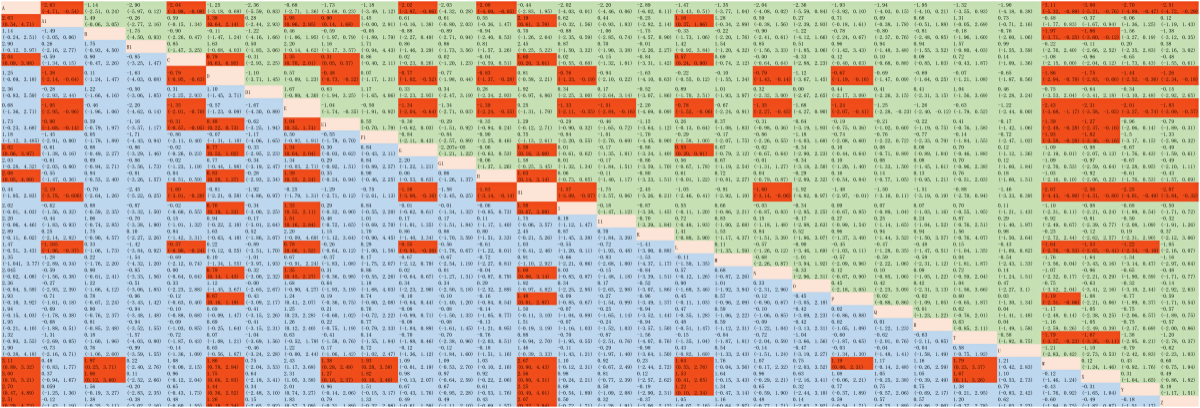
Indirect comparison of the accuracy of large language models (LLMs) when answering objective questions: A: instructGPT; A1: LLaMA 2; B: GTP-3; B1: LLaMA 3; C: ChatGPT-3.5; D: ChatGPT-4; D1: Mistral Large; E: ChatGPT-4o; E1: people; F1: chatENT; G: Bard; G1: ChatSonic; H: PaLM2; H1: Aeyeconsult; I: Gemini; I1: Med-PaLM 2; K: Gemini 1.5 pro; L: Bing chat; M: Copilot; N: Perplexity; O: Perplexity Pro; P: Claude; Q: Claude-instant; R: Claude 2; T: Claude 3 Opus; U: Claude 3 Sonnet; W: LLaMA 7B; X: LLaMA 13B; Y: LLaMA 33B; Z: LLaMA 65B.

**Figure 4:**
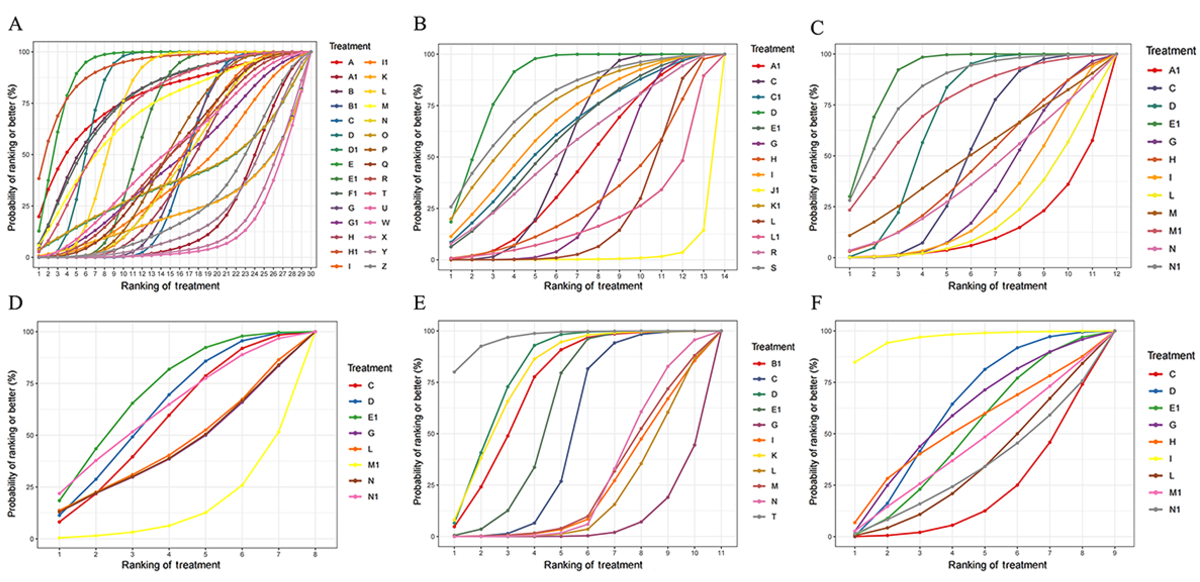
Surface under the cumulative ranking curve (SUCRAs) for the accuracy, with higher rankings associated with larger outcome values, of different large language models (LLMs) when answering (A) objective questions, (B) open-ended questions, (C) the top 1 diagnosis, (D) the top 3 diagnosis, (E) the top 5 diagnosis, and (F) triage and classification questions. The letters in the keys indicate the following LLMs: A: instructGPT; A1: LLaMA 2; B: GTP-3; B1: LLaMA 3; C: ChatGPT-3.5; C1: Mixtral-8x7B; D: ChatGPT-4; D1: Mistral Large; E: ChatGPT-4o; E1: people; F1: chatENT; G: Bard; G1: ChatSonic; H: PaLM2; H1: Aeyeconsult; I: Gemini; I1: Med-PaLM 2; J1: OcularBERT; K: Gemini 1.5 pro; K1: Doctor GPT; L: Bing chat; L1: Docs-GPT Beta; M: Copilot; M1: WebMD; N: Perplexity; N1: Ada Health; O: Perplexity Pro; P: Claude; Q: Claude-instant; R: Claude 2; S: Claude 2.1; T: Claude 3 Opus; U: Claude 3 Sonnet; W: LLaMA 7B; X: LLaMA 13B; Y: LLaMA 33B; Z: LLaMA 65B.

The accuracy of LLMs when answering objective questions was reported in 105 studies [10,13,14,20-121]. The evidence network relationships are plotted in Figure 2A and involve 30 LLMs and a total of 33,838 multiple choice questions. Direct and indirect comparisons were formed for each LLM, partially forming a closed loop. The results of the indirect comparison are shown in Figure 3 and Multimedia Appendix 7. The red cells indicate there are statistically significant differences between the column-defining regimen and the row-defining regimen. The values in the green and blue cells are the logOR and 95% CI, respectively, from the comparison of the LLMs represented in the columns with the LLMs represented in the rows. A logOR value <0 indicates that the accuracy of the LLM corresponding to a column is lower than the LLM corresponding to a row. A value >0 indicates a higher accuracy. There was no evidence of statistically significant inconsistency (all *P*>.05) in the node-splitting test for NMA, except for Claude 2 versus ChatGPT-4 (*P*=.04), Bing chat versus people (*P*=.004), and Perplexity versus people (*P*=.04; Multimedia Appendix 8). The convergence of iterations was evaluated as good in trace and density plots, with the bandwidth tending toward 0 and reaching stability (Multimedia Appendix 9). The best probability ranking showed that ChatGPT-4o (SUCRA=0.9207) ranked first in terms of accuracy when answering objective questions, Aeyeconsult (SUCRA=0.9187) ranked second, and ChatGPT-4 (SUCRA=0.8087) ranked third (Table 1, Figure 4A).

**Table 1:** Bayesian ranking results (surface under the cumulative ranking curve [SUCRA] value) of the network meta-analysis for each large language model (LLM).

LLM	SUCRA					
	Objective questions	Open-ended questions	Top 1 diagnosis	Top 3 diagnosis	Top 5 diagnosis	Triage and classification
instructGPT (A)	0.7805	—^a^	—	—	—	—
LLaMA 2 (A1)	0.2086	0.4629	0.1395	—	—	—
GTP-3 (B)	0.7704	—	—	—	—	—
LLaMA 3 (B1)	0.239	—	—	—	0.7405	—
ChatGPT-3.5 (C)	0.4343	0.5548	0.5039	0.565	0.5084	0.2093
Mixtral-8x7B (C1)	—	0.6224	—	—	—	—
ChatGPT-4 (D)	0.8087	0.8708	0.693	0.6302	0.8089	0.6185
Mistral Large (D1)	0.3842	—	—	—	—	—
ChatGPT-4o (E)	0.9207	—	—	—	—	—
People (E1)	0.6172	0.6067	0.9001	0.7126	0.6241	0.4934
chatENT (F1)	0.7687	—	—	—	—	—
Bard (G)	0.4443	0.3512	0.3353	0.4329	0.0722	0.5885
ChatSonic (G1)	0.4617	—	—	—	—	—
PaLM2 (H)	0.421	0.312	0.4496	—	—	0.5197
Aeyeconsult (H1)	0.9187	—	—	—	—	—
Gemini (I)	0.4543	0.6703	0.2812	—	0.2405	0.9649
Med-PaLM 2 (I1)	0.3919	—	—	—	—	—
OcularBERT (J1)	—	0.0176	—	—	—	—
Gemini 1.5 pro (K)	0.2449	—	—	—	0.7905	—
Doctor GPT (K1)	—	0.745	—	—	—	—
Bing chat (L)	0.728	0.23	0.2073	0.4499	0.2042	0.3391
Docs-GPT Beta (L1)	—	0.212	—	—	—	—
Copilot (M)	0.7038	—	0.5048	—	0.2633	—
WebMD (M1)	—	—	0.7511	0.1452	—	0.4348
Perplexity (N)	0.4424	—	0.3980	0.4367	0.2801	—
Ada Health (N1)	—	—	0.8363	0.6273	—	0.3319
Perplexity Pro (O)	0.3821	—	—	—	—	—
Claude (P)	0.5048	—	—	—	—	—
Claude-instant (Q)	0.4949	—	—	—	—	—
Claude 2 (R)	0.4928	0.5647	—	—	—	—
Claude 3 Opus (T)	0.7365	—	—	—	0.9672	—
Claude 3 Sonnet (U)	0.5094	—	—	—	—	—
LLaMA 7B (W)	0.1131	—	—	—	—	—
LLaMA 13B (X)	0.1365	—	—	—	—	—
LLaMA 33B (Y)	0.2147	—	—	—	—	—
LLaMA 65B (Z)	0.2721	—	—	—	—	—

^a^Not applicable because the LLM was not in the network.

#### Subgroup Analysis

**Figure 5:**
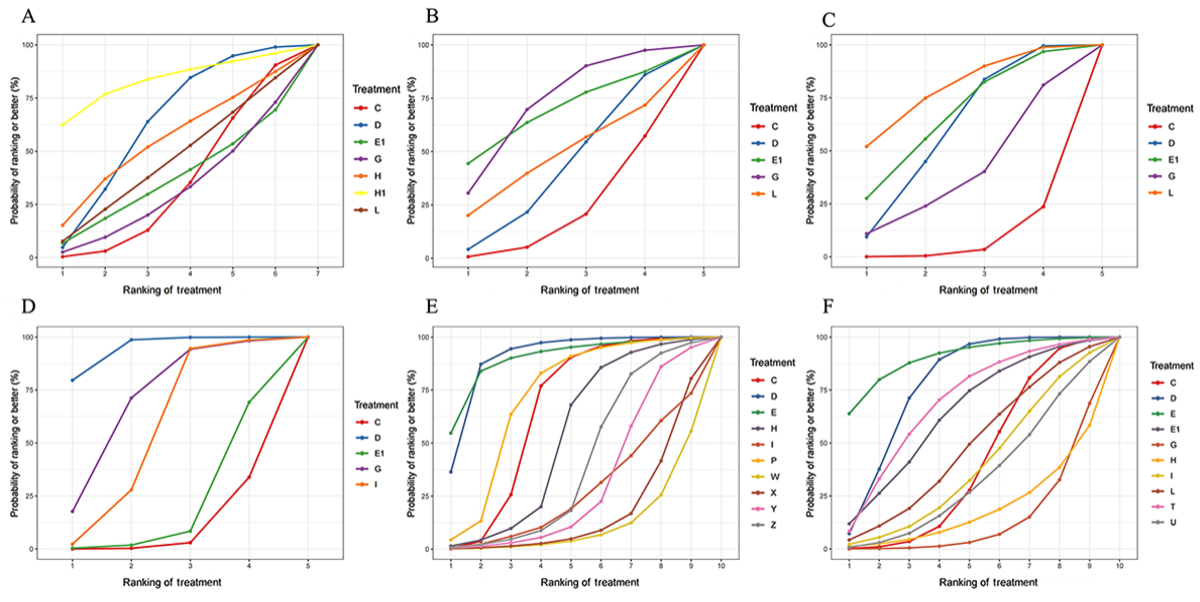
Surface under the cumulative ranking curve (SUCRAs) for the accuracy, with higher rankings associated with larger outcome values, of different large language models (LLMs) in (A) ophthalmology, (B) orthopedics, (C) urology, (D) dentistry, (E) oncology, and (F) radiology. The letters in the keys indicate the following LLMs: C: ChatGPT-3.5; D: ChatGPT-4; E: ChatGPT-4o; E1=people; G: Bard; H: PaLM2; H1: Aeyeconsult; I: Gemini; L: Bing chat; P: Claude; T: Claude 3 Opus; U: Claude 3 Sonnet; W: LLaMA 7B; X: LLaMA 13B; Y: LLaMA 33B; Z: LLaMA 65B.

We stratified the results based on the fields of the problem (Multimedia Appendix 10). Based on the results, we compared the accuracy of LLMs in 6 fields: ophthalmology, orthopedics, urology, dentistry, oncology, and radiology. In ophthalmology, the LLM with the highest accuracy was Aeyeconsult (SUCRA=0.8334), followed by ChatGPT-4 (SUCRA=0.6331) and PaLM2 (SUCRA=0.5517). In the field of orthopedics, the LLM accuracy rates, from highest to lowest, were for Bard (SUCRA=0.7219), people (SUCRA=0.6802), and Bing chat (SUCRA=0.4732). For urology, Bing chat (SUCRA=0.7905) was the most accurate, followed by people (SUCRA=0.6587) and ChatGPT-4 (SUCRA=0.5941). In dentistry, ChatGPT-4 (SUCRA=0.9473) was the most accurate, followed by Bard (SUCRA=0.7068) and Gemini (SUCRA=0.5535). ChatGPT-4 (SUCRA=0.9002) performed the best in oncology, followed by ChatGPT-4o (SUCRA=0.8998) and Claude (SUCRA=0.7159). In radiology, ChatGPT-4o (SUCRA=0.9053) performed the best, ChatGPT-4 (SUCRA=0.7777) was second, and Claude 3 Opus (SUCRA=0.6935) ranked third. The SUCRAs are shown in Figure 5.

#### Open-Ended Questions

The accuracy of the LLMs when responding to open-ended questions was examined in 34 studies [122-155]. The relationships within the evidence network are plotted in Figure 2B and include 14 LLMs and a total of 2026 open-ended questions. Direct and indirect comparisons were formed for each LLM, partially forming a closed loop. The results of the indirect comparison are presented in Multimedia Appendix 10, where red cells indicate statistically significant differences between the column-defining regimen and the row-defining regimen (Multimedia Appendix 7). There was no evidence of a statistically significant inconsistency (all *P*>.05) in the node-splitting test for the NMA, except for Bard versus ChatGPT-3.5 (*P*=.02; Multimedia Appendix 8). The trace and density plots are shown in Multimedia Appendix 9**,** and from the results, the iterative convergence was good. The best probability ranking indicated that ChatGPT-4 (SUCRA=0.8708) exhibited the highest accuracy when answering open-ended questions, followed by Claude 2.1 (SUCRA=0.7796) and Doctor GPT (SUCRA=0.7450; Table 1, Figure 4B).

#### Top 1 Diagnosis, Top 3 Diagnosis, and Top 5 Diagnosis

The accuracy of the top 1 diagnosis in clinical cases by LLMs was reported in 19 studies [11,156-173]. The evidence network relationship diagram is shown in Figure 2C and involves 12 LLMs and a total of 1266 clinical cases. The accuracy of LLMs for the top 3 diagnosis was reported in 7 studies [158,161,169,171,174-176]. The evidence network relationships are plotted in Figure 2D and involve 8 LLMs and a total of 453 clinical cases. The accuracy of LLMs for the top 5 diagnosis in clinical cases was reported in 7 studies [158,167,168,173,177-179]. The evidence network relationships are plotted in Figure 2E and involve 11 LLMs and a total of 443 clinical cases. Each LLM formed direct and indirect comparisons, partially closing the loop.

In terms of the top 1 diagnosis and top 5 diagnosis, the results of the indirect comparison are presented in Multimedia Appendix 7, where red cells indicate statistically significant differences between the column-defining regimen and the row-defining regimen. For the top 3 diagnosis, there was no statistical difference (all *P*>.05) in the comparisons between the LLMs (Multimedia Appendix 7). There was no evidence of a statistically significant inconsistency (all *P*>.05) for the top 1 diagnosis, except for Ada Health versus ChatGPT-3.5 (*P*=.04). For the top 3 diagnosis and top 5 diagnosis, all *P* were >.05 in the node-splitting test for the NMA (Multimedia Appendix 8). Iterative convergence was good, as shown by the trace and density plots (Multimedia Appendix 9). The best probability ranking showed that, in terms of accuracy of the top 1 diagnosis in clinical cases, people ranked first (SUCRA=0.9001), Ada Health ranked second (SUCRA=0.8363), and WebMD ranked third (SUCRA=0.7511; Table 1, Figure 4C). In terms of the accuracy of the top 3 diagnosis, people ranked first (SUCRA=0.7126), ChatGPT-4 ranked second (SUCRA=0.6302), and Ada Health ranked third (SUCRA=0.6273; Table 1, Figure 4D). For the accuracy of the top 5 diagnosis, Claude 3 Opus ranked first (SUCRA=0.9672), ChatGPT-4 ranked second (SUCRA=0.8089), and Gemini 1.5 pro ranked third (SUCRA=0.7905; Table 1, Figure 4E).

#### Triage and Classification

The accuracy of LLMs in triage and classification was reported in 7 studies [12,167,169,174,180-182]. The evidence network relationships are plotted in Figure 2F and involve 9 LLMs and a total of 901 clinical cases. Each LLM formed direct and indirect comparisons, partially closing the loop. The results of the indirect comparison are shown in Multimedia Appendix 7. There were significant differences between Gemini and ChatGPT-3.5, ChatGPT-4, or Bing chat (*P<*.05). There was no evidence of a statistically significant inconsistency (all *P*>.05) in the node-splitting test for the NMA, except for ChatGPT-3.5 versus ChatGPT-4 (*P*=.045; Multimedia Appendix 8). Iterative convergence was good, as shown by the trace and density plots (Multimedia Appendix 9). The best probability ranking showed that, for the accuracy of triage and classification, Gemini ranked first (SUCRA=0.9649), ChatGPT-4 ranked second (SUCRA=0.6185), and Bard ranked third (SUCRA=0.5885), as shown in Table 1 and Figure 4F.

## Discussion

### Principal Findings

This study presents the most comprehensive meta-analysis to date on the accuracy of various LLMs when responding to medical queries, encompassing objective questions, open-ended questions, top 1 diagnosis, top 3 diagnosis, top 5 diagnosis, and triage and classification. Variations in accuracy among different LLMs were observed. ChatGPT-4o demonstrated the highest accuracy when answering objective questions, while ChatGPT-4 excelled at open-ended questions. The superior performance of people at the top 1 diagnosis and top 3 diagnosis suggests that human expertise is generally more dependable than LLMs in complex medical scenarios, while Claude 3 Opus seems to perform the best in the top 5 diagnosis. In terms of triage and classification, Gemini appeared to be more reliable.

In addition, we stratified LLMs according to the medical field in which the objective questions were located and explored their accuracy in 6 fields: ophthalmology, orthopedics, urology, dentistry, oncology, and radiology. We found that Aeyeconsult performed the best in ophthalmology, Bard performed the best in orthopedics, Bing chat performed the best in urology, ChatGPT-4 performed the best in both dentistry and oncology, and ChatGPT-4o had the highest accuracy in radiology.

At present, language models based on transformer architecture, whether pretrained or fine-tuned using biomedical corpora, have been proven effective in a series of natural language processing benchmarks in the biomedical field [183]. We attempted to analyze the reasons for the performance differences when different LLMs answer questions. Parameter size is an important factor affecting the accuracy of LLMs when answering questions. Research has found that, when the parameter size of the PaLM model is expanded from 8B to 40B, the accuracy of answering medical questions is doubled [184]. However, the practicality of a model depends not only on its number of parameters but also on many factors such as its training data and architecture, fine-tuning protocols, and overall architecture [185]. Taking GPT-4 as an example, it achieved a higher performance than its predecessor by adopting more advanced training data and architecture. The timeliness and accuracy of training data are also crucial for model performance. Today, models can not only rely on a limited set of pretraining data but also obtain the latest knowledge from the internet in real time. For example, Bing AI and Google Bard already have the ability to obtain real-time updates, and ChatGPT has also begun to follow suit by accepting plugins to expand its capabilities [185,186].

In addition, we found that some models fine-tuned on the backend LLM can achieve higher accuracy and less energy consumption in specific fields. For example, in the field of ophthalmology, Aeyeconsult integrates many ophthalmic data sets based on GPT-4 for training and generation [24]. This targeted training can significantly improve its performance in ophthalmic clinical tasks. Other possible data sources include clinical texts and accurate medical information, such as guidelines and peer-reviewed literature. In fact, there are already some models built or fine-tuned based on clinical text, such as SkinGPT-4 and ChatDoctor, which perform better overall than various general LLMs at biomedical natural language processing tasks [187,188].

Progress on various grand prognostic models has been very rapid, with a newer, more arithmetically powerful version being released every few months. However, our results show that the newer versions do not necessarily outperform the older ones in terms of performance when measured as accuracy, possibly because the newer versions incorporate fewer studies, which may have biased the results somewhat. In addition, updated versions such as ChatGPT-4V provided multimodal models (eg, that can evaluate image problems), and these models may have a greater advantage for image evaluation, for example.

Studies indicate LLMs outperform humans at exams like medical licensing, orthopedics, and pediatrics globally, highlighting LLMs’ potential as a study aid. For the top 1 diagnosis and top 3 diagnosis, human accuracy is higher than that of LLMs. Despite the fact that Claude 3 Opus outperformed humans in the top 5 diagnostic results, due to the high level of accuracy required in the medical field and the multifaceted information and complex decision-making involved in medical diagnosis, we still recommend that LLMs should only be used as an auxiliary tool to assist doctors with more efficient data analysis and preliminary diagnostic recommendations.

Several meta-analyses have been conducted to assess the accuracy of LLMs in health care [15,189,190]. However, it is very unfortunate that the LLMs included in these studies included ChatGPT only and that some of the studies simply evaluated its performance on exams. Some studies did not differentiate between the types of questions answered by ChatGPT, which led to a significant amount of heterogeneity between the studies, resulting in biased results.

We acknowledge certain limitations in our study. First, for the top 3 diagnosis, top 5 diagnosis, and triage and classification, this may bias the results due to the number of included studies as well as the sample size, so caution is needed when interpreting these results. Although we minimized the heterogeneity of the research as much as possible, we cannot deny that the inclusion of different fields of study and the complexity of LLMs (such as different instructions and questioning dates) can affect the results of the study and generate heterogeneity. Therefore, caution should be exercised when interpreting the results. In addition, we did not assess the accuracy of multimodal grand prognostic models when solving medical image–related problems; with the development of artificial intelligence, more multimodal models are being developed, and in the future, these models will become indispensable in the exploration of image-based problems in the medical field.

### Conclusion

Existing studies suggest that ChatGPT-4o has an advantage for answering objective questions. For open-ended questions, ChatGPT-4 may be more credible. Humans are more accurate in the top 1 diagnosis and top 3 diagnosis of clinical cases. Claude 3 Opus performs better in the top 5 diagnosis, while for classification accuracy, Gemini is more advantageous. Although some LLMs excel at addressing medical queries, caution is advised due to the critical need for precision and rigor in medicine. Future high-quality studies and trials are necessary to gather more scientific evidence.

## Appendix

Multimedia Appendix 1 PRISMA checklist.

Multimedia Appendix 2 Search strategy.

Multimedia Appendix 3 Versions and timelines of LLMs iterations.

Multimedia Appendix 4 Examples of the outcomes.

Multimedia Appendix 5 Description of 168 studies included.

Multimedia Appendix 6 Quality assessment of observational study.

Multimedia Appendix 7 Indirect comparison results.

Multimedia Appendix 8 Node splitting inconsistency test.

Multimedia Appendix 9 Trace and density plots.

Multimedia Appendix 10 Objective questions are stratified according to different fields of the questions.

## Abbreviations

API: application programming interface
CINEMA: Confidence in Network Meta-Analysis
GRADE: Grading of Recommendations Assessment, Development, and Evaluation
LLM: large language model
NMA: network meta-analysis
OR: odds ratio
PRISMA: Preferred Reporting Items for Systematic Reviews and Meta-Analyses
SUCRA: surface under the cumulative ranking curve

## Data Availability

The data sets generated or analyzed during this study are available from the corresponding author on reasonable request.

## References

[1] Shen Y, Heacock L, Elias J, Hentel KD, Reig B, Shih G, Moy L (2023). ChatGPT and other large language models are double-edged swords. Radiology.

[2] No authors listed (2023). Will ChatGPT transform healthcare?. Nat Med.

[3] Park SH, Pinto-Powell R, Thesen T, Lindqwister A, Levy J, Chacko R, Gonzalez D, Bridges C, Schwendt A, Byrum T, Fong J, Shasavari S, Hassanpour S (2024). Preparing healthcare leaders of the digital age with an integrative artificial intelligence curriculum: a pilot study. Med Educ Online.

[4] Sblendorio E, Dentamaro V, Lo Cascio A, Germini F, Piredda M, Cicolini G (2024). Integrating human expertise and automated methods for a dynamic and multi-parametric evaluation of large language models' feasibility in clinical decision-making. Int J Med Inform.

[5] Mu Y, He D (2024). The potential applications and challenges of ChatGPT in the medical field. IJGM.

[6] Park Y, Pillai A, Deng J, Guo E, Gupta M, Paget M, Naugler C (2024). Assessing the research landscape and clinical utility of large language models: a scoping review. BMC Med Inform Decis Mak.

[7] Copilot. Microsoft.

[8] Gemini. Google.

[9] Llama. Meta.

[10] Tsoutsanis P, Tsoutsanis A (2024). Evaluation of large language model performance on the Multi-Specialty Recruitment Assessment (MSRA) exam. Comput Biol Med.

[11] Shukla R, Mishra A, Banerjee N, Verma A (2024). The comparison of ChatGPT 3.5, Microsoft Bing, and Google Gemini for diagnosing cases of neuro-ophthalmology. Cureus.

[12] Pressman SM, Borna S, Gomez-Cabello CA, Haider SA, Forte AJ (2024). AI in hand surgery: assessing large language models in the classification and management of hand injuries. J Clin Med.

[13] Vaishya R, Iyengar KP, Patralekh MK, Botchu R, Shirodkar K, Jain VK, Vaish A, Scarlat MM (2024). Effectiveness of AI-powered chatbots in responding to orthopaedic postgraduate exam questions-an observational study. Int Orthop.

[14] Lee Y, Tessier L, Brar K, Malone S, Jin D, McKechnie T, Jung JJ, Kroh M, Dang JT, ASMBS Artificial Intelligence and Digital Surgery Taskforce (2024). Performance of artificial intelligence in bariatric surgery: comparative analysis of ChatGPT-4, Bing, and Bard in the American Society for Metabolic and Bariatric Surgery textbook of bariatric surgery questions. Surg Obes Relat Dis.

[15] Wei Q, Yao Z, Cui Y, Wei B, Jin Z, Xu X (2024). Evaluation of ChatGPT-generated medical responses: a systematic review and meta-analysis. J Biomed Inform.

[16] Kaboudi N, Firouzbakht S, Shahir Eftekhar Mohammad, Fayazbakhsh Fatemeh, Joharivarnoosfaderani Niloufar, Ghaderi Salar, Dehdashti Mohammadreza, Mohtasham Kia Yasmin, Afshari Maryam, Vasaghi-Gharamaleki Maryam, Haghani Leila, Moradzadeh Zahra, Khalaj Fattaneh, Mohammadi Zahra, Hasanabadi Zahra, Shahidi Ramin (2024). Diagnostic accuracy of ChatGPT for patients' triage; a systematic review and meta-analysis. Arch Acad Emerg Med.

[17] Patil A, Serrato P, Chisvo N, Arnaout O, See PA, Huang KT (2024). Large language models in neurosurgery: a systematic review and meta-analysis. Acta Neurochir (Wien).

[18] Nguyen HC, Dang HP, Nguyen TL, Hoang V, Nguyen VA (2025). Accuracy of latest large language models in answering multiple choice questions in dentistry: a comparative study. PLoS One.

[19] Lo CK, Mertz D, Loeb M (2014). Newcastle-Ottawa Scale: comparing reviewers' to authors' assessments. BMC Med Res Methodol.

[20] Long C, Subburam D, Lowe K, Dos Santos André, Zhang J, Hwang S, Saduka N, Horev Y, Su T, Côté David W J, Wright ED (2024). ChatENT: augmented large language model for expert knowledge retrieval in otolaryngology-head and neck surgery. Otolaryngol Head Neck Surg.

[21] Tao BK, Hua N, Milkovich J, Micieli JA (2024). ChatGPT-3.5 and Bing Chat in ophthalmology: an updated evaluation of performance, readability, and informative sources. Eye (Lond).

[22] Shieh A, Tran B, He G, Kumar M, Freed JA, Majety P (2024). Assessing ChatGPT 4.0's test performance and clinical diagnostic accuracy on USMLE STEP 2 CK and clinical case reports. Sci Rep.

[23] Sarangi PK, Narayan RK, Mohakud S, Vats A, Sahani D, Mondal H (2024). Assessing the capability of ChatGPT, Google Bard, and Microsoft Bing in solving radiology case vignettes. Indian J Radiol Imaging.

[24] Singer MB, Fu JJ, Chow J, Teng CC (2024). Development and evaluation of Aeyeconsult: a novel ophthalmology chatbot leveraging verified textbook knowledge and GPT-4. J Surg Educ.

[25] Hanna RE, Smith LR, Mhaskar R, Hanna K (2024). Performance of language models on the family medicine in-training exam. Fam Med.

[26] Kadoya N, Arai K, Tanaka S, Kimura Y, Tozuka R, Yasui K, Hayashi N, Katsuta Y, Takahashi H, Inoue K, Jingu K (2024). Assessing knowledge about medical physics in language-generative AI with large language model: using the medical physicist exam. Radiol Phys Technol.

[27] Sallam M, Al-Mahzoum K, Almutawaa RA, Alhashash JA, Dashti RA, AlSafy DR, Almutairi RA, Barakat M (2024). The performance of OpenAI ChatGPT-4 and Google Gemini in virology multiple-choice questions: a comparative analysis of English and Arabic responses. BMC Res Notes.

[28] Gravina AG, Pellegrino R, Palladino G, Imperio G, Ventura A, Federico A (2024). Charting new AI education in gastroenterology: cross-sectional evaluation of ChatGPT and perplexity AI in medical residency exam. Dig Liver Dis.

[29] Passby L, Jenko N, Wernham A (2024). Performance of ChatGPT on specialty certificate examination in dermatology multiple-choice questions. Clin Exp Dermatol.

[30] Sabri H, Saleh MHA, Hazrati P, Merchant K, Misch J, Kumar PS, Wang H, Barootchi S (2025). Performance of three artificial intelligence (AI)-based large language models in standardized testing; implications for AI-assisted dental education. J Periodontal Res.

[31] Çamur Eren, Cesur T, Güneş Yasin Celal (2024). Can large language models be new supportive tools in coronary computed tomography angiography reporting?. Clin Imaging.

[32] Lubitz M, Latario L (2024). Performance of two artificial intelligence generative language models on the orthopaedic in-training examination. Orthopedics.

[33] Gupta R, Hamid A, Jhaveri M, Patel N, Suthar P (2024). Comparative evaluation of AI models such as ChatGPT 3.5, ChatGPT 4.0, and Google Gemini in neuroradiology diagnostics. Cureus.

[34] Lee G, Hong D, Kim S, Kim Jong Won, Lee Young Hwan, Park Sang O, Lee Kyeong Ryong (2024). Comparison of the problem-solving performance of ChatGPT-3.5, ChatGPT-4, Bing Chat, and Bard for the Korean emergency medicine board examination question bank. Medicine (Baltimore).

[35] Is EE, Menekseoglu AK (2024). Comparative performance of artificial intelligence models in rheumatology board-level questions: evaluating Google Gemini and ChatGPT-4o. Clin Rheumatol.

[36] D'Anna Gennaro, Van Cauter S, Thurnher M, Van Goethem J, Haller S (2024). Can large language models pass official high-grade exams of the European Society of Neuroradiology courses? A direct comparison between OpenAI chatGPT 3.5, OpenAI GPT4 and Google Bard. Neuroradiology.

[37] Altamimi I, Alhumimidi A, Alshehri S, Alrumayan Abdullah, Al-Khlaiwi Thamir, Meo Sultan A, Temsah Mohamad-Hani (2024). The scientific knowledge of three large language models in cardiology: multiple-choice questions examination-based performance. Ann Med Surg (Lond).

[38] Schoch J, Schmelz H, Strauch A, Borgmann H, Nestler T (2024). Performance of ChatGPT-3.5 and ChatGPT-4 on the European Board of Urology (EBU) exams: a comparative analysis. World J Urol.

[39] May M, Körner-Riffard Katharina, Kollitsch L, Burger M, Brookman-May SD, Rauchenwald M, Marszalek M, Eredics K (2024). Evaluating the efficacy of AI chatbots as tutors in urology: a comparative analysis of responses to the 2022 In-Service Assessment of the European Board of Urology. Urol Int.

[40] Sadeq MA, Ghorab RMF, Ashry MH, Abozaid AM, Banihani HA, Salem M, Aisheh MTA, Abuzahra S, Mourid MR, Assker MM, Ayyad M, Moawad MHED (2024). AI chatbots show promise but limitations on UK medical exam questions: a comparative performance study. Sci Rep.

[41] Khalpey Z, Kumar U, King N, Abraham A, Khalpey A (2024). Large language models take on cardiothoracic surgery: a comparative analysis of the performance of four models on American Board of Thoracic Surgery exam questions in 2023. Cureus.

[42] Patel EA, Fleischer L, Filip P, Eggerstedt M, Hutz M, Michaelides E, Batra PS, Tajudeen BA (2024). Comparative performance of ChatGPT 3.5 and GPT4 on rhinology standardized board examination questions. OTO Open.

[43] Irmici G, Cozzi A, Della Pepa G, De Berardinis C, D'Ascoli Elisa, Cellina M, Cè Maurizio, Depretto C, Scaperrotta G (2024). How do large language models answer breast cancer quiz questions? A comparative study of GPT-3.5, GPT-4 and Google Gemini. Radiol Med.

[44] Kollitsch L, Eredics K, Marszalek M, Rauchenwald M, Brookman-May SD, Burger M, Körner-Riffard Katharina, May M (2024). How does artificial intelligence master urological board examinations? A comparative analysis of different large language models' accuracy and reliability in the 2022 In-Service Assessment of the European Board of Urology. World J Urol.

[45] Morreel S, Verhoeven V, Mathysen D (2024). Microsoft Bing outperforms five other generative artificial intelligence chatbots in the Antwerp University multiple choice medical license exam. PLOS Digit Health.

[46] Bajčetić M, Mirčić A, Rakočević J, Đoković D, Milutinović K, Zaletel I (2024). Comparing the performance of artificial intelligence learning models to medical students in solving histology and embryology multiple choice questions. Ann Anat.

[47] Canillas Del Rey F, Canillas Arias M (2025). Exploring the potential of artificial intelligence in traumatology: conversational answers to specific questions. Rev Esp Cir Ortop Traumatol.

[48] Meyer A, Riese J, Streichert T (2024). Comparison of the performance of GPT-3.5 and GPT-4 with that of medical students on the written German Medical Licensing Examination: observational study. JMIR Med Educ.

[49] Toyama Y, Harigai A, Abe M, Nagano M, Kawabata M, Seki Y, Takase K (2024). Performance evaluation of ChatGPT, GPT-4, and Bard on the official board examination of the Japan Radiology Society. Jpn J Radiol.

[50] Touma NJ, Caterini J, Liblk K (2024). Is ChatGPT ready for primetime? Performance of artificial intelligence on a simulated Canadian urology board exam. Can Urol Assoc J.

[51] Chan J, Dong T, Angelini G (2024). The performance of large language models in intercollegiate Membership of the Royal College of Surgeons examination. Ann R Coll Surg Engl.

[52] Patil NS, Huang RS, van der Pol CB, Larocque N (2024). Comparative performance of ChatGPT and Bard in a text-based radiology knowledge assessment. Can Assoc Radiol J.

[53] Hubany S, Scala F, Hashemi K, Kapoor Saumya, Fedorova Julia R, Vaccaro Matthew J, Ridout Rees P, Hedman Casey C, Kellogg Brian C, Leto Barone Angelo A (2024). ChatGPT-4 surpasses residents: a study of artificial intelligence competency in plastic surgery in-service examinations and its advancements from ChatGPT-3.5. Plast Reconstr Surg Glob Open.

[54] Nakajima N, Fujimori T, Furuya M, Kanie Yuya, Imai Hirotatsu, Kita Kosuke, Uemura Keisuke, Okada Seiji (2024). A comparison between GPT-3.5, GPT-4, and GPT-4V: can the large language model (ChatGPT) pass the Japanese Board of Orthopaedic Surgery examination?. Cureus.

[55] Thibaut G, Dabbagh A, Liverneaux P (2024). Does Google's Bard Chatbot perform better than ChatGPT on the European hand surgery exam?. Int Orthop.

[56] Lum Z, Collins D, Dennison S, Guntupalli Lohitha, Choudhary Soham, Saiz Augustine M, Randall Robert L (2024). Generative artificial intelligence performs at a second-year orthopedic resident level. Cureus.

[57] Menekşeoğlu AK, İş EE (2024). Comparative performance of artificial ıntelligence models in physical medicine and rehabilitation board-level questions. Rev Assoc Med Bras (1992).

[58] Cheong RCT, Pang KP, Unadkat S, Mcneillis V, Williamson A, Joseph J, Randhawa P, Andrews P, Paleri V (2024). Performance of artificial intelligence chatbots in sleep medicine certification board exams: ChatGPT versus Google Bard. Eur Arch Otorhinolaryngol.

[59] Mesnard Benoît, Schirmann Aurélie, Branchereau Julien, Perrot Ophélie, Bogaert Guy, Neuzillet Yann, Lebret Thierry, Madec François-Xavier (2024). Artificial intelligence: ready to pass the European Board examinations in urology?. Eur Urol Open Sci.

[60] Ming S, Guo Q, Cheng W, Lei B (2024). Influence of model evolution and system roles on ChatGPT's performance in Chinese medical licensing exams: comparative study. JMIR Med Educ.

[61] Chow R, Hasan S, Zheng A, Gao C, Valdes G, Yu F, Chhabra A, Raman S, Choi JI, Lin H, Simone CB (2024). The accuracy of artificial intelligence ChatGPT in oncology examination questions. J Am Coll Radiol.

[62] Kim SE, Lee JH, Choi BS, Han H, Lee MC, Ro DH (2024). Performance of ChatGPT on solving orthopedic board-style questions: a comparative analysis of ChatGPT 3.5 and ChatGPT 4. Clin Orthop Surg.

[63] Oura T, Tatekawa H, Horiuchi D, Matsushita S, Takita H, Atsukawa N, Mitsuyama Y, Yoshida A, Murai K, Tanaka R, Shimono T, Yamamoto A, Miki Y, Ueda D (2024). Diagnostic accuracy of vision-language models on Japanese diagnostic radiology, nuclear medicine, and interventional radiology specialty board examinations. Jpn J Radiol.

[64] Lewandowski M, Łukowicz Paweł, Świetlik Dariusz, Barańska-Rybak Wioletta (2024). ChatGPT-3.5 and ChatGPT-4 dermatological knowledge level based on the Specialty Certificate Examination in Dermatology. Clin Exp Dermatol.

[65] Knoedler L, Alfertshofer M, Knoedler S, Hoch CC, Funk PF, Cotofana S, Maheta B, Frank K, Brébant Vanessa, Prantl L, Lamby P (2024). Pure wisdom or Potemkin villages? A comparison of ChatGPT 3.5 and ChatGPT 4 on USMLE Step 3 style questions: quantitative analysis. JMIR Med Educ.

[66] Khan AA, Yunus R, Sohail M, Rehman TA, Saeed S, Bu Y, Jackson CD, Sharkey A, Mahmood F, Matyal R (2024). Artificial intelligence for anesthesiology board-style examination questions: role of large language models. J Cardiothorac Vasc Anesth.

[67] Sheikh MS, Thongprayoon C, Qureshi F, Suppadungsuk S, Kashani KB, Miao J, Craici IM, Cheungpasitporn W (2024). Personalized medicine transformed: ChatGPT's contribution to continuous renal replacement therapy alarm management in intensive care units. J Pers Med.

[68] Mayo-Yáñez Miguel, Lechien JR, Maria-Saibene A, Vaira LA, Maniaci A, Chiesa-Estomba CM (2024). Examining the performance of ChatGPT 3.5 and Microsoft Copilot in otolaryngology: a comparative study with otolaryngologists' evaluation. Indian J Otolaryngol Head Neck Surg.

[69] Rydzewski NR, Dinakaran D, Zhao SG, Ruppin E, Turkbey B, Citrin DE, Patel KR (2024). Comparative evaluation of LLMs in clinical oncology. NEJM AI.

[70] Wang T, Mu J, Chen J, Lin C (2024). Comparing ChatGPT and clinical nurses' performances on tracheostomy care: a cross-sectional study. Int J Nurs Stud Adv.

[71] Liang R, Zhao A, Peng L, Xu X, Zhong J, Wu F, Yi F, Zhang S, Wu S, Hou J (2024). Enhanced artificial intelligence strategies in renal oncology: iterative optimization and comparative analysis of GPT 3.5 versus 4.0. Ann Surg Oncol.

[72] Jaworski A, Jasiński Dawid, Jaworski W, Hop Aleksandra, Janek Artur, Sławińska Barbara, Konieczniak Lena, Rzepka Maciej, Jung Maximilian, Sysło Oliwia, Jarząbek Victoria, Błecha Zuzanna, Haraziński Konrad, Jasińska Natalia (2024). Comparison of the performance of artificial intelligence versus medical professionals in the Polish final medical examination. Cureus.

[73] Bharatha A, Ojeh N, Fazle Rabbi AM, Campbell M, Krishnamurthy K, Layne-Yarde R, Kumar A, Springer D, Connell K, Majumder MA (2024). Comparing the performance of ChatGPT-4 and medical students on MCQs at varied levels of Bloom’s taxonomy. AMEP.

[74] Le M, Davis M (2024). ChatGPT yields a passing score on a pediatric board preparatory exam but raises red flags. Global Pediatric Health.

[75] Arango S, Flynn J, Zeitlin J, Lorenzana Daniel J, Miller Andrew J, Wilson Matthew S, Strohl Adam B, Weiss Lawrence E, Weir Tristan B (2024). The performance of ChatGPT on the American Society for Surgery of the Hand self-assessment examination. Cureus.

[76] Rojas M, Rojas M, Burgess V, Toro-Pérez Javier, Salehi S (2024). Exploring the performance of ChatGPT versions 3.5, 4, and 4 with vision in the Chilean medical licensing examination: observational study. JMIR Med Educ.

[77] Chau RCW, Thu KM, Yu OY, Hsung RT, Lo ECM, Lam WYH (2024). Performance of generative artificial intelligence in dental licensing examinations. Int Dent J.

[78] Thirunavukarasu AJ, Mahmood S, Malem A, Foster WP, Sanghera R, Hassan R, Zhou S, Wong SW, Wong YL, Chong YJ, Shakeel A, Chang Y, Tan BKJ, Jain N, Tan TF, Rauz S, Ting DSW, Ting DSJ (2024). Large language models approach expert-level clinical knowledge and reasoning in ophthalmology: a head-to-head cross-sectional study. PLOS Digit Health.

[79] Bicknell BT, Butler D, Whalen S, Ricks J, Dixon CJ, Clark AB, Spaedy O, Skelton A, Edupuganti N, Dzubinski L, Tate H, Dyess G, Lindeman B, Lehmann LS (2024). ChatGPT-4 Omni performance in USMLE disciplines and clinical skills: comparative analysis. JMIR Med Educ.

[80] Haddad F, Saade JS (2024). Performance of ChatGPT on ophthalmology-related questions across various examination levels: observational study. JMIR Med Educ.

[81] Noda R, Izaki Y, Kitano F, Komatsu J, Ichikawa D, Shibagaki Y (2024). Performance of ChatGPT and Bard in self-assessment questions for nephrology board renewal. Clin Exp Nephrol.

[82] Yudovich MS, Makarova E, Hague CM, Raman JD (2024). Performance of GPT-3.5 and GPT-4 on standardized urology knowledge assessment items in the United States: a descriptive study. J Educ Eval Health Prof.

[83] Li D, Kao Y, Tsai S, Bai Y, Yeh T, Chu C, Hsu C, Cheng S, Hsu T, Liang C, Su K (2024). Comparing the performance of ChatGPT GPT-4, Bard, and Llama-2 in the Taiwan Psychiatric Licensing Examination and in differential diagnosis with multi-center psychiatrists. Psychiatry Clin Neurosci.

[84] Farhat F, Chaudhry BM, Nadeem M, Sohail SS, Madsen D? (2024). Evaluating large language models for the National Premedical Exam in India: comparative analysis of GPT-3.5, GPT-4, and Bard. JMIR Med Educ.

[85] Gilson A, Safranek CW, Huang T, Socrates V, Chi L, Taylor RA, Chartash D (2023). How does ChatGPT perform on the United States Medical Licensing Examination (USMLE)? The implications of large language models for medical education and knowledge assessment. JMIR Med Educ.

[86] Kung JE, Marshall C, Gauthier C, Gonzalez TA, Jackson JB (2023). Evaluating ChatGPT performance on the orthopaedic in-training examination. JB JS Open Access.

[87] Gencer A, Aydin S (2023). Can ChatGPT pass the thoracic surgery exam?. Am J Med Sci.

[88] Ali R, Tang OY, Connolly ID, Zadnik Sullivan PL, Shin JH, Fridley JS, Asaad WF, Cielo D, Oyelese AA, Doberstein CE, Gokaslan ZL, Telfeian AE (2023). Performance of ChatGPT and GPT-4 on neurosurgery written board examinations. Neurosurgery.

[89] Massey PA, Montgomery C, Zhang AS (2023). Comparison of ChatGPT-3.5, ChatGPT-4, and orthopaedic resident performance on orthopaedic assessment examinations. J Am Acad Orthop Surg.

[90] Suchman K, Garg S, Trindade AJ (2023). Chat Generative Pretrained Transformer fails the multiple-choice American College of Gastroenterology self-assessment test. Am J Gastroenterol.

[91] Sakai D, Maeda T, Ozaki A, Kanda G, Kurimoto Y, Takahashi M (2023). Performance of ChatGPT in board examinations for specialists in the Japanese Ophthalmology Society. Cureus.

[92] Huang Y, Gomaa A, Semrau S, Haderlein M, Lettmaier S, Weissmann T, Grigo J, Tkhayat HB, Frey B, Gaipl U, Distel L, Maier A, Fietkau R, Bert C, Putz F (2023). Benchmarking ChatGPT-4 on a radiation oncology in-training exam and Red Journal Gray Zone cases: potentials and challenges for AI-assisted medical education and decision making in radiation oncology. Front Oncol.

[93] Yanagita Y, Yokokawa D, Uchida S, Tawara J, Ikusaka M (2023). Accuracy of ChatGPT on medical questions in the National Medical Licensing Examination in Japan: evaluation study. JMIR Form Res.

[94] Teebagy S, Colwell L, Wood E, Yaghy A, Faustina M (2023). Improved performance of ChatGPT-4 on the OKAP Examination: a comparative study with ChatGPT-3.5. J Acad Ophthalmol (2017).

[95] Kaneda Y, Takahashi R, Kaneda U, Akashima S, Okita H, Misaki S, Yamashiro A, Ozaki A, Tanimoto T (2023). Assessing the performance of GPT-3.5 and GPT-4 on the 2023 Japanese Nursing Examination. Cureus.

[96] Flores-Cohaila JA, García-Vicente Abigaíl, Vizcarra-Jiménez Sonia F, De la Cruz-Galán Janith P, Gutiérrez-Arratia Jesús D, Quiroga Torres BG, Taype-Rondan A (2023). Performance of ChatGPT on the Peruvian National Licensing Medical Examination: cross-sectional study. JMIR Med Educ.

[97] Fowler T, Pullen S, Birkett L (2024). Performance of ChatGPT and Bard on the official part 1 FRCOphth practice questions. Br J Ophthalmol.

[98] Moshirfar M, Altaf AW, Stoakes IM, Tuttle JJ, Hoopes PC (2023). Artificial intelligence in ophthalmology: a comparative analysis of GPT-3.5, GPT-4, and human expertise in answering StatPearls questions. Cureus.

[99] Brin D, Sorin V, Vaid A, Soroush A, Glicksberg BS, Charney AW, Nadkarni G, Klang E (2023). Comparing ChatGPT and GPT-4 performance in USMLE soft skill assessments. Sci Rep.

[100] Miao J, Thongprayoon C, Garcia Valencia OA, Krisanapan P, Sheikh MS, Davis PW, Mekraksakit P, Suarez MG, Craici IM, Cheungpasitporn W (2023). Performance of ChatGPT on nephrology test questions. CJASN.

[101] Kaneda Y, Namba M, Kaneda U, Tanimoto T (2023). Artificial intelligence in childcare: assessing the performance and acceptance of ChatGPT responses. Cureus.

[102] Takagi S, Watari T, Erabi A, Sakaguchi K (2023). Performance of GPT-3.5 and GPT-4 on the Japanese Medical Licensing Examination: comparison study. JMIR Med Educ.

[103] Ali R, Tang OY, Connolly ID, Fridley JS, Shin JH, Zadnik Sullivan PL, Cielo D, Oyelese AA, Doberstein CE, Telfeian AE, Gokaslan ZL, Asaad WF (2023). Performance of ChatGPT, GPT-4, and Google Bard on a neurosurgery oral boards preparation question bank. Neurosurgery.

[104] Ohta K, Ohta S (2023). The Performance of GPT-3.5, GPT-4, and Bard on the Japanese National Dentist Examination: a comparison study. Cureus.

[105] Watari T, Takagi S, Sakaguchi K, Nishizaki Y, Shimizu T, Yamamoto Y, Tokuda Y (2023). Performance comparison of ChatGPT-4 and Japanese medical residents in the General Medicine In-Training Examination: comparison study. JMIR Med Educ.

[106] Roos J, Kasapovic A, Jansen T, Kaczmarczyk R (2023). Artificial intelligence in medical education: comparative analysis of ChatGPT, Bing, and medical students in Germany. JMIR Med Educ.

[107] Guillen-Grima F, Guillen-Aguinaga S, Guillen-Aguinaga L, Alas-Brun R, Onambele L, Ortega W, Montejo R, Aguinaga-Ontoso E, Barach P, Aguinaga-Ontoso I (2023). Evaluating the efficacy of ChatGPT in navigating the Spanish Medical Residency Entrance Examination (MIR): promising horizons for AI in clinical medicine. Clin Pract.

[108] Huang RS, Lu KJQ, Meaney C, Kemppainen J, Punnett A, Leung F (2023). Assessment of resident and AI chatbot performance on the University of Toronto family medicine residency progress test: comparative study. JMIR Med Educ.

[109] Schubert MC, Wick W, Venkataramani V (2023). Performance of large language models on a neurology board-style examination. JAMA Netw Open.

[110] Torres-Zegarra BC, Rios-Garcia W, Ñaña-Cordova Alvaro Micael, Arteaga-Cisneros KF, Chalco XCB, Ordoñez Marina Atena Bustamante, Rios CJG, Godoy CAR, Quezada KLTP, Gutierrez-Arratia JD, Flores-Cohaila JA (2023). Performance of ChatGPT, Bard, Claude, and Bing on the Peruvian National Licensing Medical Examination: a cross-sectional study. J Educ Eval Health Prof.

[111] Kirshteyn G, Golan R, Chaet M (2024). Performance of ChatGPT vs. HuggingChat on OB-GYN topics. Cureus.

[112] van Nuland M, Erdogan A, Aςar Cenkay, Contrucci R, Hilbrants S, Maanach L, Egberts T, van der Linden PD (2024). Performance of ChatGPT on factual knowledge questions regarding clinical pharmacy. J Clin Pharmacol.

[113] Danesh A, Pazouki H, Danesh F, Danesh A, Vardar‐Sengul S (2024). Artificial intelligence in dental education: ChatGPT's performance on the periodontic in‐service examination. Journal of Periodontology.

[114] Huang CY, Zhang E, Caussade M, Brown T, Stockton Hogrogian G, Yan AC (2024). Pediatric dermatologists versus AI bots: evaluating the medical knowledge and diagnostic capabilities of ChatGPT. Pediatr Dermatol.

[115] Fiedler B, Azua EN, Phillips T, Ahmed AS (2024). ChatGPT performance on the American Shoulder and Elbow Surgeons maintenance of certification exam. J Shoulder Elbow Surg.

[116] Coleman MC, Moore JN (2024). Two artificial intelligence models underperform on examinations in a veterinary curriculum. J Am Vet Med Assoc.

[117] Abbas A, Rehman MS, Rehman SS (2024). Comparing the performance of popular large language models on the National Board of Medical Examiners sample questions. Cureus.

[118] Jarou ZJ, Dakka A, McGuire D, Bunting L (2024). ChatGPT versus human performance on emergency medicine board preparation questions. Ann Emerg Med.

[119] Sensoy E, Citirik M (2024). Assessing the proficiency of artificial intelligence programs in the diagnosis and treatment of cornea, conjunctiva, and eyelid diseases and exploring the advantages of each other benefits. Cont Lens Anterior Eye.

[120] Guerra GA, Hofmann HL, Le JL, Wong AM, Fathi A, Mayfield CK, Petrigliano FA, Liu JN (2025). ChatGPT, Bard, and Bing chat are large language processing models that answered orthopaedic in-training examination questions with similar accuracy to first-year orthopaedic surgery residents. Arthroscopy.

[121] Agarwal M, Goswami A, Sharma Priyanka (2023). Evaluating ChatGPT-3.5 and Claude-2 in answering and explaining conceptual medical physiology multiple-choice questions. Cureus.

[122] Cheong KX, Zhang C, Tan T, Fenner BJ, Wong WM, Teo KY, Wang YX, Sivaprasad S, Keane PA, Lee CS, Lee AY, Cheung CMG, Wong TY, Cheong Y, Song SJ, Tham YC (2024). Comparing generative and retrieval-based chatbots in answering patient questions regarding age-related macular degeneration and diabetic retinopathy. Br J Ophthalmol.

[123] Zhou S, Luo X, Chen C, Jiang Hong, Yang Chun, Ran Guanghui, Yu Juan, Yin Chengliang (2024). The performance of large language model-powered chatbots compared to oncology physicians on colorectal cancer queries. Int J Surg.

[124] Kozaily E, Geagea M, Akdogan ER, Atkins J, Elshazly MB, Guglin M, Tedford RJ, Wehbe RM (2024). Accuracy and consistency of online large language model-based artificial intelligence chat platforms in answering patients' questions about heart failure. Int J Cardiol.

[125] Xia S, Hua Q, Mei Z, Xu W, Lai L, Wei M, Qin Y, Luo L, Wang C, Huo S, Fu L, Zhou F, Wu J, Zhang L, Lv D, Li J, Wang X, Li N, Song Y, Zhou J (2025). Clinical application potential of large language model: a study based on thyroid nodules. Endocrine.

[126] Lee Y, Shin T, Tessier L, Javidan A, Jung J, Hong D, Strong AT, McKechnie T, Malone S, Jin D, Kroh M, Dang JT, ASMBS Artificial Intelligence and Digital Surgery Task Force (2024). Harnessing artificial intelligence in bariatric surgery: comparative analysis of ChatGPT-4, Bing, and Bard in generating clinician-level bariatric surgery recommendations. Surg Obes Relat Dis.

[127] Doğan L, Özçakmakcı Gazi Bekir, Yılmaz E (2024). The performance of chatbots and the AAPOS website as a tool for amblyopia education. J Pediatr Ophthalmol Strabismus.

[128] Lee T, Campbell D, Patel S, Hossain Afif, Radfar Navid, Siddiqui Emaad, Gardin Julius M (2024). Unlocking health literacy: the ultimate guide to hypertension education from ChatGPT versus Google Gemini. Cureus.

[129] Lang SP, Yoseph ET, Gonzalez-Suarez AD, Kim R, Fatemi P, Wagner K, Maldaner N, Stienen MN, Zygourakis CC (2024). Analyzing large language models' responses to common lumbar spine fusion surgery questions: a comparison between ChatGPT and Bard. Neurospine.

[130] Iannantuono G, Bracken-Clarke D, Karzai F, Choo-Wosoba H, Gulley J, Floudas C (2024). Comparison of large language models in answering immuno-oncology questions: a cross-sectional study. Oncologist.

[131] Anguita R, Downie C, Ferro Desideri L, Sagoo MS (2024). Assessing large language models' accuracy in providing patient support for choroidal melanoma. Eye (Lond).

[132] Zhang Y, Dong Y, Mei Z, Hou Y, Wei M, Yeung YH, Xu J, Hua Q, Lai L, Li N, Xia S, Zhou C, Zhou J (2024). Performance of large language models on benign prostatic hyperplasia frequently asked questions. Prostate.

[133] Xue E, Bracken-Clarke D, Iannantuono GM, Choo-Wosoba H, Gulley JL, Floudas CS (2024). Utility of large language models for health care professionals and patients in navigating hematopoietic stem cell transplantation: comparison of the performance of ChatGPT-3.5, ChatGPT-4, and Bard. J Med Internet Res.

[134] Cao JJ, Kwon DH, Ghaziani TT, Kwo P, Tse G, Kesselman A, Kamaya A, Tse JR (2024). Large language models' responses to liver cancer surveillance, diagnosis, and management questions: accuracy, reliability, readability. Abdom Radiol (NY).

[135] Monroe CL, Abdelhafez YG, Atsina K, Aman E, Nardo L, Madani MH (2024). Evaluation of responses to cardiac imaging questions by the artificial intelligence large language model ChatGPT. Clin Imaging.

[136] Chervonski E, Harish KB, Rockman CB, Sadek M, Teter KA, Jacobowitz GR, Berland TL, Lohr J, Moore C, Maldonado TS (2025). Generative artificial intelligence chatbots may provide appropriate informational responses to common vascular surgery questions by patients. Vascular.

[137] Kassab J, Hadi El Hajjar A, Wardrop RM, Brateanu A (2024). Accuracy of online artificial intelligence models in primary care settings. Am J Prev Med.

[138] Al-Sharif E, Penteado R, Dib El Jalbout Nahia, Topilow Nicole J, Shoji Marissa K, Kikkawa Don O, Liu Catherine Y, Korn Bobby S (2024). Evaluating the accuracy of ChatGPT and Google BARD in fielding oculoplastic patient queries: a comparative study on artificial versus human intelligence. Ophthalmic Plast Reconstr Surg.

[139] Mejia MR, Arroyave JS, Saturno M, Ndjonko LCM, Zaidat B, Rajjoub R, Ahmed W, Zapolsky I, Cho SK (2024). Use of ChatGPT for determining clinical and surgical treatment of lumbar disc herniation with radiculopathy: a North American Spine Society guideline comparison. Neurospine.

[140] Lee T, Rao A, Campbell D, Radfar N, Dayal M, Khrais A (2024). Evaluating ChatGPT-3.5 and ChatGPT-4.0 responses on hyperlipidemia for patient education. Cureus.

[141] Oliveira AL, Coelho M, Guedes LC, Cattoni MB, Carvalho H, Duarte-Batista P (2024). Performance of ChatGPT 3.5 and 4 as a tool for patient support before and after DBS surgery for Parkinson's disease. Neurol Sci.

[142] Lim ZW, Pushpanathan K, Yew SME, Lai Y, Sun C, Lam JSH, Chen DZ, Goh JHL, Tan MCJ, Sheng B, Cheng C, Koh VTC, Tham Y (2023). Benchmarking large language models' performances for myopia care: a comparative analysis of ChatGPT-3.5, ChatGPT-4.0, and Google Bard. EBioMedicine.

[143] Rahsepar AA, Tavakoli N, Kim GHJ, Hassani C, Abtin F, Bedayat A (2023). How AI responds to common lung cancer questions: ChatGPT vs Google Bard. Radiology.

[144] Pushpanathan K, Lim ZW, Er Yew SM, Chen DZ, Hui'En Lin HA, Lin Goh JH, Wong WM, Wang X, Jin Tan MC, Chang Koh VT, Tham Y (2023). Popular large language model chatbots' accuracy, comprehensiveness, and self-awareness in answering ocular symptom queries. iScience.

[145] Coskun BN, Yagiz B, Ocakoglu G, Dalkilic E, Pehlivan Y (2024). Assessing the accuracy and completeness of artificial intelligence language models in providing information on methotrexate use. Rheumatol Int.

[146] King RC, Samaan JS, Yeo YH, Peng Y, Kunkel DC, Habib AA, Ghashghaei R (2024). A multidisciplinary assessment of ChatGPT's knowledge of amyloidosis: observational study. JMIR Cardio.

[147] Pinto VBP, de Azevedo MF, Wroclawski ML, Gentile G, Jesus VLM, de Bessa Junior J, Nahas WC, Sacomani CAR, Sandhu JS, Gomes CM (2024). Conformity of ChatGPT recommendations with the AUA/SUFU guideline on postprostatectomy urinary incontinence. Neurourol Urodyn.

[148] Momenaei B, Wakabayashi T, Shahlaee A, Durrani AF, Pandit SA, Wang K, Mansour HA, Abishek RM, Xu D, Sridhar J, Yonekawa Y, Kuriyan AE (2024). Assessing ChatGPT-3.5 versus ChatGPT-4 performance in surgical treatment of retinal diseases: a comparative study. Ophthalmic Surg Lasers Imaging Retina.

[149] Stevenson E, Walsh C, Hibberd L (2024). Can artificial intelligence replace biochemists? A study comparing interpretation of thyroid function test results by ChatGPT and Google Bard to practising biochemists. Ann Clin Biochem.

[150] Dronkers EAC, Geneid A, Al Yaghchi C, Lechien JR (2024). Evaluating the potential of AI chatbots in treatment decision-making for acquired bilateral vocal fold paralysis in adults. J Voice.

[151] Rahimli Ocakoglu S, Coskun B (2024). The emerging role of AI in patient education: a comparative analysis of LLM accuracy for pelvic organ prolapse. Med Princ Pract.

[152] Gandhi AP, Joesph FK, Rajagopal V, Aparnavi P, Katkuri S, Dayama S, Satapathy P, Khatib MN, Gaidhane S, Zahiruddin QS, Behera A (2024). Performance of ChatGPT on the India undergraduate community medicine examination: cross-sectional study. JMIR Form Res.

[153] Tariq R, Malik S, Khanna S (2024). Evolving landscape of large language models: an evaluation of ChatGPT and Bard in answering patient queries on colonoscopy. Gastroenterology.

[154] Li P, Zhang X, Zhu E, Yu S, Sheng B, Tham YC, Wong TY, Ji H (2024). Potential multidisciplinary use of large language models for addressing queries in cardio-oncology. J Am Heart Assoc.

[155] Sosa BR, Cung M, Suhardi VJ, Morse K, Thomson A, Yang HS, Iyer S, Greenblatt MB (2024). Capacity for large language model chatbots to aid in orthopedic management, research, and patient queries. J Orthop Res.

[156] Koga S, Martin NB, Dickson DW (2024). Evaluating the performance of large language models: ChatGPT and Google Bard in generating differential diagnoses in clinicopathological conferences of neurodegenerative disorders. Brain Pathol.

[157] Warrier A, Singh R, Haleem A, Zaki H, Eloy JA (2024). The comparative diagnostic capability of large language models in otolaryngology. Laryngoscope.

[158] Kumar RP, Sivan V, Bachir H, Sarwar SA, Ruzicka F, O'Malley GR, Lobo P, Morales IC, Cassimatis ND, Hundal JS, Patel NV (2024). Can artificial intelligence mitigate missed diagnoses by generating differential diagnoses for neurosurgeons?. World Neurosurg.

[159] Hirosawa T, Harada Y, Mizuta K, Sakamoto T, Tokumasu K, Shimizu T (2024). Diagnostic performance of generative artificial intelligences for a series of complex case reports. Digit Health.

[160] Mandalos A, Tsouris D (2024). Artificial versus human intelligence in the diagnostic approach of ophthalmic case scenarios: a qualitative evaluation of performance and consistency. Cureus.

[161] Krusche M, Callhoff J, Knitza J, Ruffer N (2024). Diagnostic accuracy of a large language model in rheumatology: comparison of physician and ChatGPT-4. Rheumatol Int.

[162] Delsoz M, Madadi Y, Raja H, Munir WM, Tamm B, Mehravaran S, Soleimani M, Djalilian A, Yousefi S (2024). Performance of ChatGPT in diagnosis of corneal eye diseases. Cornea.

[163] Kozel G, Gurses ME, Gecici NN, Gökalp Elif, Bahadir S, Merenzon MA, Shah AH, Komotar RJ, Ivan ME (2024). Chat-GPT on brain tumors: an examination of artificial intelligence/machine learning's ability to provide diagnoses and treatment plans for example neuro-oncology cases. Clin Neurol Neurosurg.

[164] Stoneham S, Livesey A, Cooper H, Mitchell C (2024). ChatGPT versus clinician: challenging the diagnostic capabilities of artificial intelligence in dermatology. Clin Exp Dermatol.

[165] Albaladejo A, Lorleac'h A, Allain J (2024). [The spring of artificial intelligence: AI vs. expert for internal medicine cases]. Rev Med Interne.

[166] Zandi R, Fahey JD, Drakopoulos M, Bryan JM, Dong S, Bryar PJ, Bidwell AE, Bowen RC, Lavine JA, Mirza RG (2024). Exploring diagnostic precision and triage proficiency: a comparative study of GPT-4 and Bard in addressing common ophthalmic complaints. Bioengineering (Basel).

[167] Hirosawa T, Kawamura R, Harada Y, Mizuta K, Tokumasu K, Kaji Y, Suzuki T, Shimizu T (2023). ChatGPT-generated differential diagnosis lists for complex case-derived clinical vignettes: diagnostic accuracy evaluation. JMIR Med Inform.

[168] Hirosawa T, Harada Y, Yokose M, Sakamoto T, Kawamura R, Shimizu T (2023). Diagnostic accuracy of differential-diagnosis lists generated by Generative Pretrained Transformer 3 Chatbot for clinical vignettes with common chief complaints: a pilot study. Int J Environ Res Public Health.

[169] Fraser H, Crossland D, Bacher I, Ranney M, Madsen T, Hilliard R (2023). Comparison of diagnostic and triage accuracy of Ada Health and WebMD symptom checkers, ChatGPT, and Physicians for Patients in an emergency department: clinical data analysis study. JMIR Mhealth Uhealth.

[170] Rojas-Carabali W, Cifuentes-González Carlos, Wei X, Putera I, Sen A, Thng ZX, Agrawal R, Elze T, Sobrin L, Kempen JH, Lee B, Biswas J, Nguyen QD, Gupta V, de-la-Torre A, Agrawal R (2024). Evaluating the diagnostic accuracy and management recommendations of ChatGPT in uveitis. Ocul Immunol Inflamm.

[171] Gräf Markus, Knitza J, Leipe J, Krusche M, Welcker M, Kuhn S, Mucke J, Hueber AJ, Hornig J, Klemm P, Kleinert S, Aries P, Vuillerme N, Simon D, Kleyer A, Schett G, Callhoff J (2022). Comparison of physician and artificial intelligence-based symptom checker diagnostic accuracy. Rheumatol Int.

[172] Ward M, Unadkat P, Toscano D, Kashanian Alon, Lynch Daniel G, Horn Alexander C, D'Amico Randy S, Mittler Mark, Baum Griffin R (2024). A quantitative assessment of ChatGPT as a neurosurgical triaging tool. Neurosurgery.

[173] Hirosawa T, Mizuta K, Harada Y, Shimizu T (2023). Comparative evaluation of diagnostic accuracy between Google Bard and physicians. Am J Med.

[174] Lyons RJ, Arepalli SR, Fromal O, Choi JD, Jain N (2024). Artificial intelligence chatbot performance in triage of ophthalmic conditions. Can J Ophthalmol.

[175] Makhoul M, Melkane AE, Khoury PE, Hadi CE, Matar N (2024). A cross-sectional comparative study: ChatGPT 3.5 versus diverse levels of medical experts in the diagnosis of ENT diseases. Eur Arch Otorhinolaryngol.

[176] Shemer A, Cohen M, Altarescu A, Atar-Vardi M, Hecht I, Dubinsky-Pertzov B, Shoshany N, Zmujack S, Or L, Einan-Lifshitz A, Pras E (2024). Diagnostic capabilities of ChatGPT in ophthalmology. Graefes Arch Clin Exp Ophthalmol.

[177] Gunes Y, Cesur T (2024). The diagnostic performance of large language models and general radiologists in thoracic radiology cases: a comparative study. J Thorac Imaging.

[178] Sarangi PK, Irodi A, Panda S, Nayak DSK, Mondal H (2024). Radiological differential diagnoses based on cardiovascular and thoracic imaging patterns: perspectives of four large language models. Indian J Radiol Imaging.

[179] Berg HT, van Bakel B, van de Wouw L, Jie KE, Schipper A, Jansen H, O'Connor Rory D, van Ginneken B, Kurstjens S (2024). ChatGPT and generating a differential diagnosis early in an emergency department presentation. Ann Emerg Med.

[180] Haider SA, Pressman SM, Borna S, Gomez-Cabello CA, Sehgal A, Leibovich BC, Forte AJ (2024). Evaluating large language model (LLM) performance on established breast classification systems. Diagnostics (Basel).

[181] Gan RK, Ogbodo JC, Wee YZ, Gan AZ, González Pedro Arcos (2024). Performance of Google bard and ChatGPT in mass casualty incidents triage. Am J Emerg Med.

[182] Aiumtrakul N, Thongprayoon C, Arayangkool C, Vo KB, Wannaphut C, Suppadungsuk S, Krisanapan P, Garcia Valencia OA, Qureshi F, Miao J, Cheungpasitporn W (2024). Personalized medicine in urolithiasis: AI chatbot-assisted dietary management of oxalate for kidney stone prevention. J Pers Med.

[183] Wang H, Gao C, Dantona C, Hull B, Sun J (2024). DRG-LLaMA: tuning LLaMA model to predict diagnosis-related group for hospitalized patients. NPJ Digit Med.

[184] Singhal K, Azizi S, Tu T, Mahdavi SS, Wei J, Chung HW, Scales N, Tanwani A, Cole-Lewis H, Pfohl S, Payne P, Seneviratne M, Gamble P, Kelly C, Babiker A, Schärli Nathanael, Chowdhery A, Mansfield P, Demner-Fushman D, Agüera Y Arcas Blaise, Webster D, Corrado GS, Matias Y, Chou K, Gottweis J, Tomasev N, Liu Y, Rajkomar A, Barral J, Semturs C, Karthikesalingam A, Natarajan V (2023). Large language models encode clinical knowledge. Nature.

[185] Thirunavukarasu AJ, Ting DSJ, Elangovan K, Gutierrez L, Tan TF, Ting DSW (2023). Large language models in medicine. Nat Med.

[186] Researcher Access Program application. OpenAI.

[187] Zhou J, He X, Sun L, Xu J, Chen X, Chu Y, Zhou L, Liao X, Zhang B, Afvari S, Gao X (2024). Pre-trained multimodal large language model enhances dermatological diagnosis using SkinGPT-4. Nat Commun.

[188] Li Y, Li Z, Zhang K, Dan R, Jiang S, Zhang Y (2023). ChatDoctor: a medical chat model fine-tuned on a large language model meta-AI (LLaMA) using medical domain knowledge. Cureus.

[189] Bagde H, Dhopte A, Alam MK, Basri R (2023). A systematic review and meta-analysis on ChatGPT and its utilization in medical and dental research. Heliyon.

[190] Levin G, Horesh N, Brezinov Y, Meyer R (2024). Performance of ChatGPT in medical examinations: a systematic review and a meta-analysis. BJOG.

