# Supplementary material for: Accuracy of Large Language Models When Answering Clinical Research Questions: Systematic Review and Network Meta-Analysis
Source: J Med Internet Res. 2025 Apr 30;27:e64486. doi: 10.2196/64486 (PMC12079073; doi:10.2196/64486)
Supplement: Multimedia Appendix 2 [file jmir_v27i1e64486_app2.docx]

# Multimedia Appendix 2 Search strategy

| Search | Query | Results |
| --- | --- | --- |
| PubMed | | |
| #1 | (generative AI [Title/Abstract] OR open AI [Title/Abstract] OR Large language model [Title/Abstract] OR LLM [Title/Abstract] OR chatGPT [Title/Abstract] or Google [Title/Abstract] OR Bing [Title/Abstract] OR Ada Health [Title/Abstract] OR WebMD [Title/Abstract] OR Aeyeconsult [Title/Abstract] OR Perplexity [Title/Abstract] OR Claude [Title/Abstract] OR OcularBERT [Title/Abstract] OR Llama Chat [Title/Abstract] OR Gemini [Title/Abstract] OR Grok [Title/Abstract] OR PaLM [Title/Abstract]) AND (Accuracy [Title/Abstract] OR Performance [Title/Abstract] OR Evaluation [Title/Abstract]) | 15155 |
| Limit | Abstract | 14938 |
| Exclude | Preprints | **14837** |
| Embase | | |
| #1 | ('generative ai':ab,ti OR 'open ai':ab,ti OR 'large language model':ab,ti OR llm:ab,ti OR chatgpt:ab,ti OR google:ab,ti OR bing:ab,ti OR 'ada health':ab,ti OR webmd:ab,ti OR aeyeconsult:ab,ti OR perplexity:ab,ti OR claude:ab,ti OR ocularbert:ab,ti OR 'llama chat':ab,ti OR gemini:ab,ti OR grok:ab,ti OR palm:ab,ti) AND (accuracy:ab,ti OR performance:ab,ti OR evaluation:ab,ti) | 18227 |
| Limit | Abstract | 18020 |
| Exclude | Preprints | **17537** |
| Web of Science | | |
| #1 | ("generative AI" OR "open AI " OR "Large language model" OR "LLM" OR "chatGPT" or "Google" OR "Bing" OR "Ada Health" OR "WebMD" OR "Aeyeconsult" OR "Perplexity" OR "Claude" OR "OcularBERT" OR "Llama Chat" OR "Gemini" OR "Grok" OR "PaLM") (title) AND ("Accuracy" OR "Performance" OR "Evaluation") (title) | 11374 |
|  |  | **11374** |
| Scopus | | |
| #1 | ({generative AI} OR {open AI} OR {Large language model} OR {LLM} OR {chatGPT} or {Google} OR {Bing} OR {Ada Health} OR {WebMD} OR {Aeyeconsult} OR {Perplexity} OR {Claude} OR {OcularBERT} OR {Llama Chat} OR {Gemini} OR {Grok} OR {PaLM}) AND ({Accuracy} OR {Performance} OR {Evaluation}) | 15327 |
|  |  | **15327** |
