# Supplementary material for: Accuracy of Large Language Models When Answering Clinical Research Questions: Systematic Review and Network Meta-Analysis
Source: J Med Internet Res. 2025 Apr 30;27:e64486. doi: 10.2196/64486 (PMC12079073; doi:10.2196/64486)
Supplement: Multimedia Appendix 3 [file jmir_v27i1e64486_app3.docx]

# Multimedia Appendix 3 Versions and timelines of LLMs iterations

| Time | Event |
| --- | --- |
| **OpenAI** | |
| 2020.5 | OpenAI releases **GPT-3**, the world's largest pre-trained language model at the time. |
| 2022.1 | OpenAI releases the predecessor of ChatGPT, **InstructionGPT** model, which allows users to calibrate and fine tune the model. |
| 2022.11 | OpenAI has officially released the new conversational AI model ChatGPT, which was fine tuned from the **GPT-3.5** series of large-scale speech models. |
| 2023.3 | OpenAI has launched a large-scale multimodal model **GPT-4**, which can not only read text, but also recognize images and generate text results. It has now been integrated into ChatGPT and opened to Plus users. |
| 2023.3 | GPT-4 was officially released on March 14, 2023.ChatGPT Plus was launched in February 2023 as a subscription service by OpenAI. |
| 2023.9 | OpenAI releases **GPT-4V(ision)** with visual capabilities, enabling users to instruct GPT-4 to analyze user-supplied image inputs. |
| 2024.5 | OpenAI has officially released **GPT-4o**, where "o" represents "omni" (meaning comprehensive and all powerful). This model has the ability to handle text, images, videos, and speech simultaneously |
| **Google** | |
| 2023.2 | Google announced the launch of **Bard**, whose underlying model was initially **LaMDA** |
|  | **Bard** upgraded from a lightweight LaMDA-based model to a larger scale **PaLM** model. |
| 2023.5 | Google announced **PaLM2**, a 340 billion parameter model trained on 3.6 trillion tokens, at the annual Google I/O keynote |
| 2023.12 | Google has officially announced the launch of **Gemini 1.0**, which consists of three tiers: Gemini Ultra for the most powerful, Gemini Pro for multitasking, and Gemini Nano for specific tasks and end-sides. |
| 2024.2 | （2024.2.15）, we rolled out our most capable model, Gemini 1.0 Ultra（the version with Ultra will be called Gemini Advanced）, and took a significant step forward in making Google products more helpful, starting with **Gemini Advanced**. |
| 2024.2 | Bard has been fully upgraded with Gemini Pro as the underlying big model driver, and Google has announced a new era of Gemini. Starting today, Bard will be renamed Gemini. |
| 2024.2 | Google has upgraded the Gemini family of models and released **Gemini version 1.5** for early testing. |
| 2024.4 | Google Launches Public Preview of **Gemini 1.5 Pro** |
| 2024.5 | Google held its I/O conference and unveiled the newest member of the Gemini family of models, the **Gemini 1.5 Flash**. |
| 2024.9 | Google Releases **Gemini 1.5-Pro-002** and **Gemini 1.5-Flash-002** |
| **Microsoft** | |
| 2023.2 | Microsoft Releases **Bing Chat** Large Language Model Based on ChatGPT-4 |
| 2023.3 | Microsoft has rebranded its AI assistant from “Microsoft Copilot for Microsoft 365″ to “Microsoft 365 **Copilot**”. This is the same name it had from its introduction in March 2023 to autumn of 2023. |
| 2023.5 | Microsoft has announced that Bing Chat is now officially in Open Preview mode, allowing anyone to use. |
| **Anthropic** | |
| 2023.3 | Anthropic officially releases the initial version of **Claude** |
| 2023.3 | On March 15, Anthropic, an AI startup founded by a former OpenAI employee, released a ChatGPT-like AI assistant called “Claude” Claude is available in two versions: “Claude” and “**Claude Instant**. Claude offers two versions: “Claude” and “Claude Instant”. |
| 2023.7 | **Claude 2** officially released |
| 2023.11 | **Claude 2.1** officially released |
| 2024.3 | The **Claude 3** series has been officially released. Claude 3 can receive text and images (such as tables, graphics, and photos) as input. Claude 3 is divided into three sub models: **Claude 3 Opus, Claude 3 Sonnet, and Claude 3 Haiku**. Opus is the most powerful model among the three, while Haiku is the faster and more cost-effective model on the market. |
| 2024.6 | Anthropic releases Claude 3.5 series. The first version released this time is **Claude 3.5 Sonnet**. Free use is already supported. |
| **Meta** | |
| 2023.2 | Meta has publicly released **Llama 1** for the first time, which includes four parameter sizes: **7B, 13b, 33B, and 65B** (where "B" represents one billion, intuitively reflecting the complexity of the model and its depth of understanding of the training data) |
| 2023.7 | Meta has released a free commercial version of **Llama-2**, which includes four parameter versions: **7B, 13b, 34B, and 70B**. Except for the 34B model, all others are open source. |
| 2024.4 | Meta has officially released the open-source large model **Llama 3**, including two parameter versions, **8B and 70B**. |
| **Perplexity** | |
| 2022.12 | Perplexity launches core product Perplexity AI: **Perplexity Ask** |
| 2023.6 | Perplexity launches **Perplexity pro** |
| **Mistral** | |
| 2023.9 | Mistral AI releases **Mistral 7B** |
| 2023.12 | Mistral AI Team Officially Launches **Mixtral 8×7B** to the Public |
| 2024.2 | On 2024.2.26, Mistral AI officially released the **Mistral Large** model and launched a conversational product that benchmarks ChatGPT. |
