# Supplementary material for: Accuracy of Large Language Models When Answering Clinical Research Questions: Systematic Review and Network Meta-Analysis
Source: J Med Internet Res. 2025 Apr 30;27:e64486. doi: 10.2196/64486 (PMC12079073; doi:10.2196/64486)
Supplement: Multimedia Appendix 4 [file jmir_v27i1e64486_app4.docx]

**Multimedia Appendix 4** Examples of objective question、open-ended question、top 1 diagnosis, top 3 diagnosis, top 5 diagnosis and triage and claasification


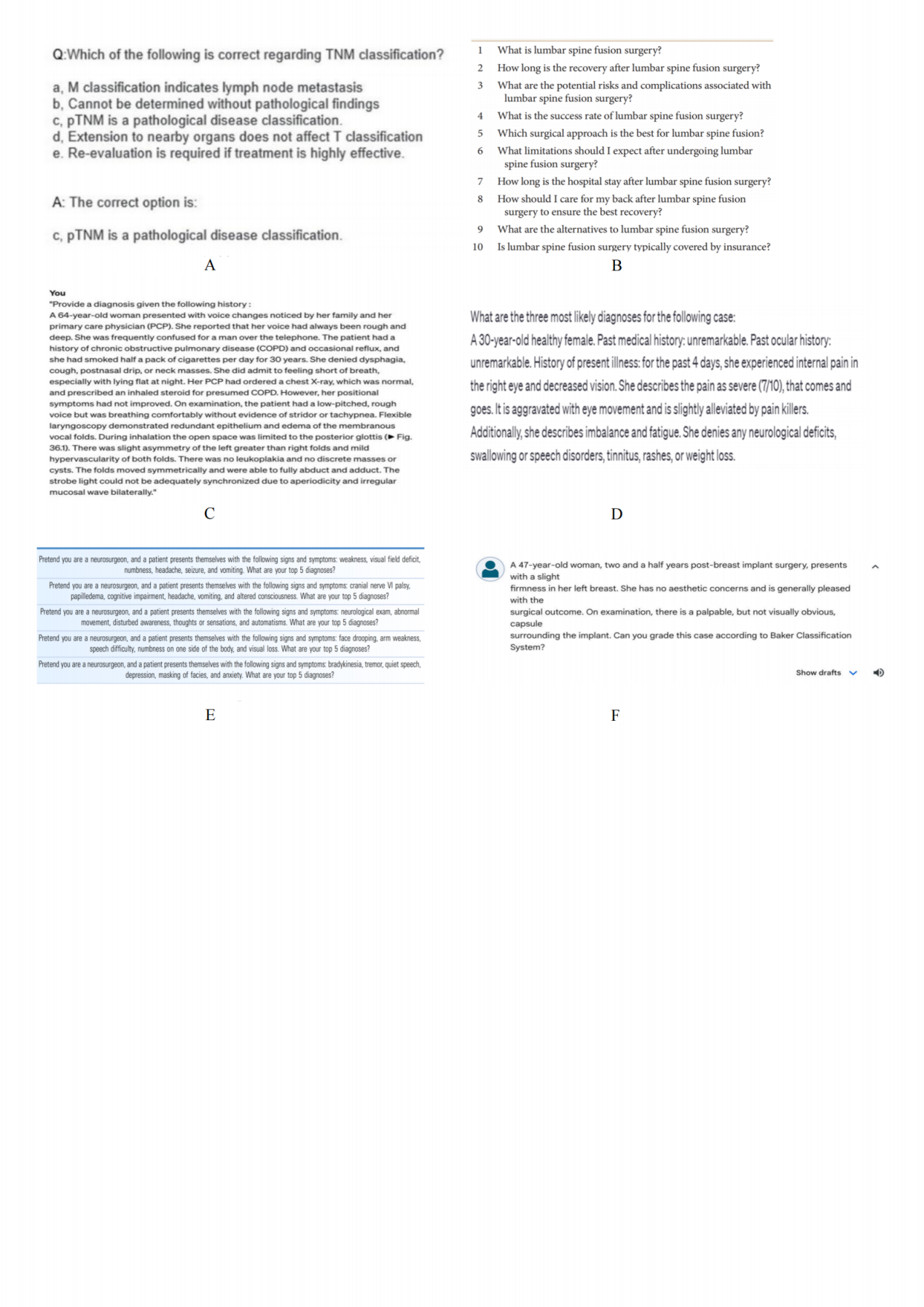


Note: A: objective question; B: open-ended question; C: top 1 diagnosis; D: top 3 diagnosis; E: top 5 diagnosis; F: triage and classification

References:

A: Kadoya N, Arai K, Tanaka S, et al. Assessing knowledge about medical physics in language-generative AI with large language model: using the medical physicist exam. Radiol Phys Technol. Published online September 10, 2024.

B: Lang SP, Yoseph ET, Gonzalez-Suarez AD, et al. Analyzing Large Language Models' Responses to Common Lumbar Spine Fusion Surgery Questions: A Comparison Between ChatGPT and Bard. Neurospine. 2024;21(2):633-641.

C: Warrier A, Singh R, Haleem A, Zaki H, Eloy JA. The Comparative Diagnostic Capability of Large Language Models in Otolaryngology. Laryngoscope. 2024;134(9):3997-4002.

D: Shemer A, Cohen M, Altarescu A, et al. Diagnostic capabilities of ChatGPT in ophthalmology. Graefes Arch Clin Exp Ophthalmol. 2024;262(7):2345-2352.

E: Kumar RP, Sivan V, Bachir H, et al. Can Artificial Intelligence Mitigate Missed Diagnoses by Generating Differential Diagnoses for Neurosurgeons?. World Neurosurg. 2024;187:e1083-e1088.

F: Haider SA, Pressman SM, Borna S, et al. Evaluating Large Language Model (LLM) Performance on Established Breast Classification Systems. Diagnostics (Basel). 2024;14(14):1491. Published 2024 Jul 11.
