# Supplementary material for: Accuracy of Large Language Models When Answering Clinical Research Questions: Systematic Review and Network Meta-Analysis
Source: J Med Internet Res. 2025 Apr 30;27:e64486. doi: 10.2196/64486 (PMC12079073; doi:10.2196/64486)
Supplement: Multimedia Appendix 5 [file jmir_v27i1e64486_app5.docx]

Multimedia Appendix 5 Description of 168 studies included

| Study ID | generative AI | Sample size | Outcome | type of question /cases | Questions /cases source | Datasets introduction | Field | Language | Access interface |
| --- | --- | --- | --- | --- | --- | --- | --- | --- | --- |
| Tsoutsanis P，2024^1^ | chatGPT-3.5 | 100 | obj. | MCQs | Qbank | one of the most popular  commercially available question banks | dermatology, ENT, medicine, musculoskeletal, ophthalmology, paediatrics, pharmacology, psychiatry, surgery, women’s health | English | / |
|  | Bard |  |  |  |  |  |  |  | / |
|  | Bing chat |  |  |  |  |  |  |  | / |
|  | Llama 2 |  |  |  |  |  |  |  | / |
|  | Qbank users |  |  |  |  |  |  |  | / |
| Long C，2024^2^ | chatGPT-4 | 160 | obj. | MCQs | BoardVitals | US board practice  question banks that were widely used by US senior OHNS residents | general ear, nose and throat (ENT), Head and Neck Surgery, Laryngology, Otology-Neurotology, Facial Plastics Surgery, Pediatrics, sleep medicine, and Rhinology | English | / |
|  | chatENT |  |  |  |  |  |  |  | / |
|  | senior‐year OHNS residents |  |  |  |  |  |  |  | / |
| Tao BK，2024^3^ | chatGPT-3.5 | 913 | obj. | MCQs | Basic and Clinical Science Course (BCSC) Resident Set collection | a key resource for ophthalmology learners to become eventual wellinformed and competent practitioners in the feld. | ophthalmology | English | / |
|  | Bing chat |  |  |  |  |  |  |  | / |
| Shieh A，2024^4^ | chatGPT-3.5 | 109 | obj. | MCQs | USMLE 2 CK | United States  Medical Licensing Exam STEP 2 | / | English | / |
|  | chatGPT-4 |  |  |  |  |  |  |  | / |
| Sarangi PK，2023^5^ | chatGPT-3.5 | 120 | obj. | MCQs | FRCR2A | Royal College of Radiologists 2A examination | radiology | English | / |
|  | Bard |  |  |  |  |  |  |  | / |
|  | Bing chat |  |  |  |  |  |  |  | / |
|  | residents |  |  |  |  |  |  |  | / |
| Singer MB，2024^6^ | chatGPT-4 | 260 | obj. | MCQs | OKAP | Ophthalmic Knowledge Assessment Program | ophthalmology | English | https://chat.openai.com/ |
|  | Ayeconsult |  |  |  |  |  |  |  | A web page, JavaScript code  that was publicly available on GitHub |
| Hanna RE，2024^7^ | chatGPT-3.5 | 193 | obj. | MCQs | family medicine in-training exam (ITE) | ITE is a 200-question multiple-choice exam, used to gauge residents’ progress throughout their training | / | English | / |
|  | chatGPT-4 |  |  |  |  |  |  |  | / |
|  | Bard |  |  |  |  |  |  |  | / |
| Kadoya N，2024^8^ | chatGPT-3.5 | 718 | obj. | MCQs | Japan’s medical physicist board examinations | consists of medicine/ biology questions and physics/engineering questions. | medical physics | Japanese | / |
|  | chatGPT-4 |  |  |  |  |  |  |  | / |
| Sallam M，2024^9^ | chatGPT-4 | 40 | obj. | MCQs | testing of medical students during the period 2017-2022 | designed by the first author (M.S.) | Virology | English/  Arabic | https://openai.com/ |
|  | Gemini |  |  |  |  |  |  |  | https://gemini.google.com/app |
| Gravina AG，2024^10^ | chatGPT-3.5 | 60 | obj. | MCQs | SSM23 | the 2023 Italiann national residency admission exam spans all fields of medicine and surgery | gastroenterology | English | https://chat.openai.com/ |
|  | Perplexity AI |  |  |  |  |  |  |  | https://www.Perplexity.ai/ |
| Passby L，2024^11^ | chatGPT-3.5 | 84 | obj. | MCQs | dermatology SCE | The Specialty Certificate Examination (SCE) in dermatology is a postgraduate examination and is a requirement for achieving specialist registration to practice as a dermatologist | dermatology | English | https://chat.openai.com/chat |
|  | chatGPT-4 |  |  |  |  |  |  |  | https://chat.openai.com/chat |
| Sabri H，2024^12^ | chatGPT-3.5 | 1312 | obj. | MCQs | examination organized by the American Academy of Periodontology | This exam comprises 10 sections spanning clinical and foundational sciences, diagnosis, and treatment planning. | dentistry | English | / |
|  | chatGPT-4 |  |  |  |  |  |  |  | / |
|  | Gemini |  |  |  |  |  |  |  | / |
|  | Residents |  |  |  |  |  |  |  | / |
| Çamur E，2024^13^ | chatGPT-4 | 30 | obj. | MCQs | CAD-RADS 2.0 | designed by Radiologist (E.Ç.) utilizing the information in  CAD-RADS 2.0 | cardiology | English | / |
|  | chatGPT-4o |  |  |  |  |  |  |  | / |
|  | Claude 3 Opus |  |  |  |  |  |  |  | / |
|  | Gemini 1.5 Pro |  |  |  |  |  |  |  | / |
|  | Mistral Large |  |  |  |  |  |  |  | / |
|  | Llama 3 70B |  |  |  |  |  |  |  | / |
|  | Perplexity Pro |  |  |  |  |  |  |  | / |
| Lubitz M，2024^14^ | chatGPT-4 | 207 | obj. | MCQs | OITE | Orthopaedic In-Training Examination is organized by AAOS, and orthopedic surgery residents should participate every year | orthopaedics | English | https://openai.com/blog/chatgpt |
|  | Bard |  |  |  |  |  |  |  | https://bard.google.com |
| Gupta R，2024^15^ | chatGPT-3.5 | 262 | obj. | MCQs | quizzes from the  Textbook  "Neuroradiology: A Core Review."/ | / | radiology | English | / |
|  | chatGPT-4 |  |  |  |  |  |  |  | / |
|  | Gemini |  |  |  |  |  |  |  | / |
| Lee GU，2024^16^ | chatGPT-3.5 | 123 | obj. | SCQs | The Korean Emergency Medicine Board Examination | It is divided into 23 detailed categories and covers. The total score is 100 points, and a score ≥ 60 points is required to pass this examination. | / | Korean | / |
|  | chatGPT-4 |  |  |  |  |  |  |  | / |
|  | Bard |  |  |  |  |  |  |  | / |
|  | Bing chat |  |  |  |  |  |  |  | / |
| Is EE，2024^17^ | chatGPT-4o | 420 | obj. | MCQs | Board level examinations | rheumatologists in the USA are required to take a 240-question multiple -choice exam. | orthopaedics | English | / |
|  | Gemini |  |  |  |  |  |  |  | / |
| D'Anna G，2024^18^ | chatGPT-3.5 | 180 | obj. | MCQs | Neuroradiology MCE exams | exams of The European Course in Neuroradiology organized by the European Society  of Neuroradiology | neuroradiology | English | / |
|  | chatGPT-4 |  |  |  |  |  |  |  | / |
|  | Bard |  |  |  |  |  |  |  | / |
| Altamimi I，2024^19^ | chatGPT-4 | 100 | obj. | MCQs | MCQ bank | The creation of the MCQ bank was a meticulous process undertaken by the research team. The team sourced questions  from a variety of authoritative textbooks and question banks. | cardiology | English | / |
|  | Bard |  |  |  |  |  |  |  | / |
|  | Bing chat |  |  |  |  |  |  |  | / |
| Lee Y，2024^20^ | chatGPT-4 | 200 | obj. | MCQs | The ASMBS Textbook of Bariatric Surgery: Second Edition | / | bariatric surgery | English | / |
|  | Bard |  |  |  |  |  |  |  | / |
|  | Bing chat |  |  |  |  |  |  |  | / |
| Schoch J，2024^21^ | chatGPT-3.5 | 600 | obj. | MCQs | In-Service Assessment  of the EBU training book | One exam included about 200 multiple-choice  questions with different subtopics like oncology, surgery, trauma, incontinence, andrology, and others. | urology | English | / |
|  | chatGPT-4 |  |  |  |  |  |  |  | / |
| May M，2024^22^ | chatGPT-3.5 | 100 | obj. | MCQs | ISA-2022 | In-Service Assessment of  the European Board of Urology | urology | English | https://chat.openai.com/ |
|  | chatGPT-4 |  |  |  |  |  |  |  | https://chat.openai.com/ |
|  | Bing chat |  |  |  |  |  |  |  | https://www.bing.com/?/ai |
| Sadeq MA，2024^23^ | chatGPT-3.5 | 333 | obj. | MCQs | UK medical board exam | board examination websites including MRCS, MRCP, RCPCH,  RCOG, RCOopth, MRCPsych, FRCR (physics), FRCA, and MCEM in addition to sample obstetrics and gynecology questions provided by BMJ | / | English | / |
|  | chatGPT-4 |  |  |  |  |  |  |  | / |
|  | Bard |  |  |  |  |  |  |  | / |
|  | Bing chat |  |  |  |  |  |  |  | / |
|  | Perplexity |  |  |  |  |  |  |  | / |
|  | Claud |  |  |  |  |  |  |  | / |
|  | Claude-instant |  |  |  |  |  |  |  | / |
| Khalpey Z，2024^24^ | chatGPT-3.5 | 400 | obj. |  | SESATS XIII question bank | Self-Education and Self-Assessment in Thoracic Surgery is a sophisticated online educational and assessment platform designed specifically for cardiothoracic surgeons | cardiothoracic  surgery | English | https://openai.com/blog/chatgpt |
|  | chatGPT-4 |  |  |  |  |  |  |  | https://openai.com/blog/chatgpt |
|  | Claude 2 |  |  |  |  |  |  |  | https://www.anthropic.com/news/claude-2 |
|  | Med-PaLM 2 |  |  |  |  |  |  |  | / |
| Patel EA，2024^25^ | chatGPT-3.5 | 93 | obj. | MCQs | American Board of Otolaryngology Written Question Exam | Questions were generated from www.boardvitals.  com, offering study  material and question banks for physicians, medical students, and others in the health‐care industry | rhinology | English | / |
|  | chatGPT-4 |  |  |  |  |  |  |  | / |
|  | Otolaryngology  residents |  |  |  |  |  |  |  | / |
| Irmici G，2024^26^ | chatGPT-3.5 | 60 | obj. | MCQs | the training sample database of the European Diploma in Breast Imaging; Diagnostic Radiology  In-Training Exam of the American College of Radiology; the practice test database of the RadiologyKey website; the online database of Medscape | / | breast cancer | English | / |
|  | chatGPT-4 |  |  |  |  |  |  |  | / |
|  | Gemini |  |  |  |  |  |  |  | / |
| Kollitsch L，2024^27^ | chatGPT-3.5 | 100 | obj. | MCQs | ISA-2022 | In-Service Assessment of  the European Board of Urology | urology | English | https://chat.openai.com/ |
|  | chatGPT-4 |  |  |  |  |  |  |  | https://chat.openai.com/ |
|  | Bing chat |  |  |  |  |  |  |  | https://www.bing.com/?/ai |
| Morreel S，2024^28^ | chatGPT-3.5 | 102 | obj. | MCQs | University of Antwerp medical licensing exam | At the end of the undergraduate medical training, medical students must pass a general medical knowledge examination before being licensed as medical doctor | / | English | Accessed through Poe |
|  | chatGPT-4 |  |  |  |  |  |  |  | Conversation style = More precise |
|  | PaLM2 |  |  |  |  |  |  |  | Accessed using a virtual private network to emulate US location |
|  | Bing chat |  |  |  |  |  |  |  | Accessed through Poe |
|  | Claude+ |  |  |  |  |  |  |  | Accessed through Poe |
|  | Claude instant |  |  |  |  |  |  |  | Accessed through Poe |
| Bajčetić M，2024^29^ | chatGPT-3.5 | 60 | obj. | MCQs | University of Belgrade  , Department of Histology and Embryology’ internal database | / | histology and embryology | English | https://chat.openai.com/ |
|  | PaLM2 |  |  |  |  |  |  |  | https://bard.google.com/ |
|  | Bing chat |  |  |  |  |  |  |  | https://www.bing.com/?/ai |
|  | Perplexity |  |  |  |  |  |  |  | https://www.perplexity.ai/ |
|  | ChatSonic |  |  |  |  |  |  |  | https://writesonic.com/chat |
|  | student |  |  |  |  |  |  |  | / |
| Canillas Del Rey F，2024^30^ | chatGPT-3.5 | 129 | obj. | MCQs | Spanish Professional Medical Training Entrance Examination | / | / | Spanish | / |
|  | Bard |  |  |  |  |  |  |  | / |
|  | Peiplexity |  |  |  |  |  |  |  | / |
| Meyer A，2024^31^ | chatGPT-3.5 | 937 | obj. | MCQs | German Medical Licensing  Examination | the third-party client Amboss, a web-based learning platform that provides the original questions from the IMPP | / | English | / |
|  | chatGPT-4 |  |  |  |  |  |  |  | / |
| Toyama Y，2024^32^ | chatGPT-4 | 103 | obj. | MCQs | JRBE | Japan Radiology Board Examination | radiology | Japanese | https://chat.openai.com/auth/login |
|  | PaLM 2 |  |  |  |  |  |  |  | https://bard.google.com |
| Touma NJ，2024^33^ | chatGPT-4 | 100 | obj. | MCQs | QUEST | Queen's Urology Exam Skill Test | urology | English | / |
|  | urology residents |  |  |  |  |  |  |  | / |
| Chan J，2024^34^ | chatGPT-3.5 | 300 | obj. | MCQs | Pastest | a commonly used question bank for junior doctors  preparing for the Membership of the Royal College of  Surgeons examination | / | English | / |
|  | chatGPT-4 |  |  |  |  |  |  |  | / |
|  | Bard |  |  |  |  |  |  |  | / |
| Patil NS，2024^35^ | chatGPT-4 | 318 | obj. | MCQs | American College of  Radiology’s Diagnostic Radiology In-Training (DXIT) examination | The DXIT examination is an annual exam prepared by the ACR used to simulate the American Board of Radiology (ABR) Core exam. | radiology | English | https://chat.openai.com/ |
|  | Bard |  |  |  |  |  |  |  | https://bard.google.com/ |
| Hubany SS，2024^36^ | chatGPT-3.5 | 1250 | obj. | MCQs | The Plastic Surgery In-service Examination (PSISE) | PSISE conducted annually for all plastic surgery residents in the United States, serves as a critical tool to gauge their progress in medical training and assess their competencies as physicians. | Plastic Surgery | English | / |
|  | chatGPT-4 |  |  |  |  |  |  |  | https://openai.com/research/gpt-4 |
| Vaishya R，2024^37^ | chatGPT-3.5 | 120 | obj. | MCQs | internationally recognized orthopaedic examinations/tests | such asSICOT Diploma and  the National Board of Examinations (NBE) in Medical Sciences in Orthopaedics | orthopedics | English | https://openai.com/blog/chatgpt/ |
|  | chatGPT-4 |  |  |  |  |  |  |  | https://openai.com/blog/chatgpt/ |
|  | Bard |  |  |  |  |  |  |  | https://bard.google.com/ |
| Nakajima N，2024^38^ | chatGPT-3.5 | 294 | obj. | MCQs | JBOSE | The Japanese Board of Orthopaedic Surgery Examination | orthopedics | English | / |
|  | chatGPT-4 |  |  |  |  |  |  |  | / |
| Thibaut G，2024^39^ | chatGPT-3.5 | 90 | obj. | MCQs | EBHS diploma examination | The European Board of Hand Surgery diploma examination questions published in the Journal of Hand Surgery (European Volume) | / | English | / |
|  | Bard |  |  |  |  |  |  |  | / |
| Lum ZC，2024^40^ | chatGPT-3.5 | 390 | obj. | MCQs | OITE | Orthopaedic In-Training Examination | orthopedics | English | / |
|  | Bard |  |  |  |  |  |  |  | / |
| Menekşeoğlu AK，2024^41^ | chatGPT-3.5 | 94 | obj. | MCQs | PMR100 | PMR100 is an example question set for the American Board of Physical Medicine and Rehabilitation Part I exam, focusing on artificial intelligence models’ ability to answer and categorize questions by difficulty | / | English | / |
|  | chatGPT-4 |  |  |  |  |  |  |  | / |
|  | Bard |  |  |  |  |  |  |  | / |
| Cheong RCT，2024^42^ | chatGPT-3.5 | 301 | obj. | MCQs | American Sleep Medicine Certifcation Board Exam | / | sleep medicine | English | https://openai.com/blog/chatgpt |
|  | chatGPT-4 |  |  |  |  |  |  |  | https://openai.com/blog/chatgpt |
|  | Bard |  |  |  |  |  |  |  | https://blog.google/technology/ai/bard-google-ai-search-updates/ |
| Mesnard B，2024^43^ | chatGPT-3.5 | 100 | obj. | MCQs | in-service assessment of the European Board of Urology | / | urological surgery | English | / |
|  | chatGPT-4 |  |  |  |  |  |  |  | / |
|  | Bard |  |  |  |  |  |  |  | / |
|  | training students |  |  |  |  |  |  |  | / |
| Ming S，2024^44^ | chatGPT-3.5 | 600 | obj. | MCQs | Chinese National Medical Licensing Examination 2022 question set | the written part of the examination, which  emphasizes medical knowledge and clinical decision-making skills, is created and supervised by the Chinese National Medical Examination Center (NMEC) | Chinese medicine | Chinese | / |
|  | chatGPT-4 |  |  |  |  |  |  |  | https://openai.com/research/gpt-4/ |
| Chow R，2024^45^ | chatGPT-3.5 | 600 | obj. | MCQs | national radiation oncology in-service multiple-choice examinations | / | oncology | English | / |
|  | chatGPT-4o |  |  |  |  |  |  |  | / |
| Kim SE，2024^46^ | chatGPT-3.5 | 160 | obj. | MCQs | Orthopedic Surgery Department at Seoul National University Hospital | it have been validated by board-certified orthopedic surgeons and adapted to align with the format of the Korean Orthopedic Association board certification examinations | orthopaedics | Korean | / |
|  | chatGPT-4 |  |  |  |  |  |  |  | / |
| Oura T，2024^47^ | chatGPT-4 | 459 | obj. | MCQs | JDR test, JNM test, JIR test | / | radiology | Japanese | https://chat.openai.  com/ |
|  | chatGPT-4o |  |  |  |  |  |  |  | https://chat.openai.  com/ |
|  | Claude 3 Sonnet |  |  |  |  |  |  |  | https://claude.ai/ |
|  | Claude 3 Opus |  |  |  |  |  |  |  | https://claude.ai/ |
| Lewandowski M，2024^48^ | chatGPT-3.5 | 358 | obj. | MCQs | Three Specialty Certificate Examination in Dermatology tests | / | Dermatology | Polish, English | / |
|  | chatGPT-4 |  |  |  |  |  |  |  | https://openai.com/research/gpt-4 |
| Knoedler L，2024^49^ | chatGPT-3.5 | 1840 | obj. | MCQs | AMBOSS question bank | / | / | English | / |
|  | chatGPT-4 |  |  |  |  |  |  |  | / |
| Khan AA，2024^50^ | chatGPT-3.5 | 884 | obj. | MCQs | *Anesthesia: A Comprehensive Review (Sixth Edition)* | regarded as one of the premium study sources for ABA certification and recertification examinations | anesthesiology | English | https://openai.com/blog/chatgpt/ |
|  | chatGPT-4 |  |  |  |  |  |  |  | https://openai.com/blog/chatgpt/ |
|  | PaLM2 |  |  |  |  |  |  |  | / |
| Sheikh MS，2024^51^ | chatGPT-3.5 | 50 | obj. | MCQs | Selected by two nephrologists in intensive care | / | nephropathy | English | https://openai.com/blog/chatgpt |
|  | chatGPT-4 |  |  |  |  |  |  |  | https://openai.com/blog/chatgpt |
| Mayo-Yáñez M，2024^52^ | chatGPT-3.5 | 135 | obj. | MCQs | otolaryngology job competition exams for the public healthcare system | / | otolaryngology | English | https://openai.com/ |
|  | Copilot |  |  |  |  |  |  |  | https://www.bing.com |
| Rydzewski NR，2024^53^ | chatGPT-3.5 | 2044 | obj. | MCQs | American College  of Radiology in-training radiation oncology examinations | / | oncology | English | https://openai.com/blog/chatgpt |
|  | chatGPT-4 |  |  |  |  |  |  |  | https://openai.com/blog/chatgpt |
|  | Claude |  |  |  |  |  |  |  | / |
|  | PaLM2 |  |  |  |  |  |  |  | / |
|  | LLaMA 7B |  |  |  |  |  |  |  | / |
|  | LLaMA 13B |  |  |  |  |  |  |  | / |
|  | LLaMA 33B |  |  |  |  |  |  |  | / |
|  | LLaMA 65B |  |  |  |  |  |  |  | / |
| Wang T，2024^54^ | chatGPT-3.5 | 43 | obj. | MCQs | tracheostomy care practice | the tracheostomy care practice survey comprises 43 items, including 16 multiple-choice questions and 27 select-all-that-apply questions. | / | English | https://openai.com |
|  | chatGPT-4 |  |  |  |  |  |  |  | https://openai.com |
|  | clinical nurses |  |  |  |  |  |  |  | / |
| Liang R，2024^55^ | chatGPT-3.5 | 80 | obj. | yes or no | urology experts | / | oncology | English | https://openai.com/blog/chatgpt |
|  | chatGPT-4 |  |  |  |  |  |  |  | https://openai.com/blog/chatgpt |
| Jaworski A，2024^56^ | chatGPT-3.5 | 196 | obj. | MCQs | Center for  Medical Education in Łódź, Poland | / | / | English | / |
|  | chatGPT-4 |  |  |  |  |  |  |  | / |
| Bharatha A，2024^57^ | chatGPT-4 | 304 | obj. | MCQs | Faculty of Medical Sciences courses | / | / | English | / |
|  | MBBS students |  |  |  |  |  |  |  | / |
| Le M，2024^58^ | chatGPT-3.5 | 490 | obj. | MCQs | Pediatrics 2021 PREP^®^, Pediatrics 2021 PREP^®^ | / | pediatrics | English | https://openai.com/about/ |
|  | chatGPT-4 |  |  |  |  |  |  |  | https://openai.com/about/ |
|  | human test takers |  |  |  |  |  |  |  | / |
| Arango SD，2024^59^ | chatGPT-3.5 | 238 | obj. | MCQs | American Society for Surgery of the Hand Self-Assessment Examination | the examination consists of  200 multiple-choice questions, with five answer choices per question | surgery | English | https://www.openai.com/chatgpt |
|  | chatGPT-4 |  |  |  |  |  |  |  | https://www.openai.com/chatgpt |
| Rojas M，2024^60^ | chatGPT-3.5 | 540 | obj. | MCQs | Examen Único Nacional de Conocimientos de Medicina practice drills | / | ophthalmology | Spanish | / |
|  | chatGPT-4 |  |  |  |  |  |  |  | https://openai.com/research/gpt-4v-system-card |
| Chau RCW，2024^61^ | chatGPT-3.5 | 1461 | obj. | MCQs | US and the  UK dental licensing examinations | / | dentistry | English | / |
|  | chatGPT-4 |  |  |  |  |  |  |  | / |
| Thirunavukarasu AJ，2024^62^ | chatGPT-3.5 | 87 | obj. | MCQs | FRCOphth Part 2 examination questions | / | ophthalmology | English | / |
|  | chatGPT-4 |  |  |  |  |  |  |  | / |
|  | PaLM2 |  |  |  |  |  |  |  | / |
|  | LLaMA2 |  |  |  |  |  |  |  | / |
| Bicknell BT，2024^63^ | chatGPT-3.5 | 750 | obj. | MCQs | various question banks provided by medical schools | / | / | English | https://openai.com/index/chatgpt/ |
|  | chatGPT-4 |  |  |  |  |  |  |  | https://openai.com/index/gpt-4/ |
|  | chatGPT-4o |  |  |  |  |  |  |  | https://openai.com/index/hellogpt-4o/ |
| Haddad F，2024^64^ | chatGPT-3.5 | 380 | obj. | MCQs | United States Medical Licensing Examination, *Ophthalmology Board Review Q&A* | / | ophthalmology | English | / |
|  | chatGPT-4 |  |  |  |  |  |  |  | / |
| Noda R，2024^65^ | chatGPT-3.5 | 99 | obj. | MCQs | Self-Assessment Questions for Nephrology Board Renewal | The Self-Assessment Questions for Nephrology Board Renewal are Japanese-written multiple-choice questions conducted annually by the Japanese Society of Nephrology | nephrology | Japanese | https://openai.com/blog/chatgpt/ |
|  | chatGPT-4 |  |  |  |  |  |  |  | https://openai.com/blog/chatgpt/ |
|  | Bard |  |  |  |  |  |  |  | https://bard.google.com. |
| Yudovich MS，2024^66^ | chatGPT-3.5 | 700 | obj. | MCQs | designed by author | / | urology | English | / |
|  | chatGPT-4 |  |  |  |  |  |  |  | / |
| Li DJ，2024^67^ | chatGPT-4 | 100 | obj. | MCQs | 2022 Taiwan  Psychiatric Licensing Examination | / | psychiatry | Chinese | / |
|  | LLaMA2 |  |  |  |  |  |  |  | / |
| Farhat F，2024^68^ | ChatGPT-3.5 | 175 | Obj. | MCQs | NEET-2023 | the National Eligibility cum Entrance Test (NEET) in India. | / | English | https://openai.com/ChatGPT |
|  | ChatGPT-34 |  |  |  |  |  |  |  | https://openai.com/ChatGPT |
|  | Bard |  |  |  |  |  |  |  | https://bard.google.com/chat |
| Gilson A，2023^69^ | Instruct GPT | 100 | Obj. | MCQs | AMBOSS | a widely used question bank that contains over 2700 Step 1 and 3150 Step 2 questions | / | English | https://openai.com/blog/chatgpt/ |
|  | GPT-3 | 120 |  |  | NBME | National Board of Medical Examiners |  |  |  |
| Kung JE，2023^70^ | ChatGPT-3.5 | 215 | Obj. | MCQs | AAOS | American Academy of Orthopaedic Surgeons | orthopedics | English | / |
|  | ChatGPT-4 |  |  |  |  |  |  |  |  |
| Gencer A，2023^71^ | ChatGPT-3.5 | 15 | Obj. | MCQs | Turkish-language thoracic surgery exam questions | After completing a one-week thoracic surgery  internship at our university, students are required to pass  both theoretical and practical exams. | thoracic | English | https://chat.openai.com |
|  | ChatGPT-4 |  |  |  |  |  |  |  |  |
|  | Students |  |  |  |  |  |  |  |  |
| Ali R，2023^72^ | ChatGPT-3.5 | 500 | Obj. | MCQs | SANS ABNS 1 | SelfAssessment Neurosurgery Exams (SANS) American Board of Neurological Surgery (ABNS)  Self-Assessment Exam 1. | neurosurgery | English | / |
|  | ChatGPT-4 |  |  |  |  |  |  |  |  |
|  | question bank users |  |  |  |  |  |  |  |  |
| Massey PA，2023^73^ | ChatGPT-3.5 | 180 | Obj. | MCQs | The ResStudy orthopaedic examination question bank, | The ResStudy orthopaedic examination question bank,  endorsed by the American Academy of Orthopaedic Surgeons (AAOS) | orthopedics | English | / |
|  | ChatGPT-4 |  |  |  |  |  |  |  |  |
|  | ortho residents |  |  |  |  |  |  |  |  |
| Suchman K，2023^74^ | ChatGPT-3 | 500 | Obj. | MCQs | ACG | 2022 and 2021 American College  of Gastroenterology (ACG) Self-Assessment Tests. | gastroenterology | English | / |
|  | ChatGPT-4 |  |  |  |  |  |  |  |  |
| Sakai D，2023^75^ | ChatGPT-3.5 | 500 | Obj. | MCQs | Board examination for a specialist in Japanese Ophthalmology Society | five sets of past board examination problems for specialists in the Japanese Ophthalmology Society (30th to 34th, available online, https://www.nichigan.or.jp/senmon/purpose/examination.html). T | ophthalmologic | Japanese | / |
|  | ChatGPT-4 |  |  |  |  |  |  |  |  |
| Huang Y，2023^76^ | ChatGPT-3.5 | 300 | Obj. | MCQs | TXIT、  2022 Red Journal Gray Zone cases | The 38th American College of Radiology (ACR) radiation oncology intraining (TXIT) exam and the 2022 Red Journal Gray Zone cases | radiation oncology | English | / |
|  | ChatGPT-4 |  |  |  |  |  |  |  |  |
| Yanagita Y，2023^77^ | ChatGPT-3.5 | 292 | Obj. | MCQs | NMLE | Questions from the National Medical Licensing Examination (NMLE) in Japan, administered by the Japanese Ministry  of Health, Labour and Welfare in 2022, were used. | / | Japanese | / |
|  | ChatGPT-4 |  |  |  |  |  |  |  |  |
| Teebagy S，2023^78^ | ChatGPT-3.5 | 180 | Obj. | MCQs | OKAP | Ophthalmology Knowledge  Assessment Program | ophthalmologic | English | / |
|  | ChatGPT-4 |  |  |  |  |  |  |  |  |
| Kaneda Y，2023^79^ | ChatGPT-3.5 | 237 | Obj. | MCQs | JNNE | Japanese National Nursing Examination | nursing | Japanese | / |
| Flores-Cohaila JA，2023^80^ | ChatGPT-3.5 | 180 | Obj. | MCQs | ENAM 2022 data set | data source was the 2022 ENAM question set  obtained directly from the official website of the Peruvian  Society of Medical Schools (ASPEFAM) | / | Spanish | / |
|  | ChatGPT-4 |  |  |  |  |  |  |  |  |
|  | examinees |  |  |  |  |  |  |  |  |
| Fowler T，2024^81^ | ChatGPT-4 | 49 | Obj. | MCQs | the part 1 FRCOphth MCQ exam | the Royal  College of Ophthalmologists website | / | English | / |
|  | Bard |  |  |  |  |  |  |  |  |
| Moshirfar M，2023^82^ | ChatGPT-3.5 | 467 | Obj. | MCQs | the StatPearls question bank | ChatGPT's performance was evaluated utilizing StatPearls ophthalmology questions, which are peer- reviewed questions from a medical question bank designed to be at the level of ophthalmology residents, fellows, and attendings. | ophthalmology | English | / |
|  | ChatGPT-4 |  |  |  |  |  |  |  |  |
|  | human professionals |  |  |  |  |  |  |  |  |
|  | ChatGPT-4 |  |  |  |  |  |  |  |  |
| Brin D，2023^83^ | ChatGPT-3.5 | 80 | Obj. | MCQs | USMLE and  AMBOSS | AMBOSS, a widely recognized question bank for medical practitioners and students. AMBOSS provides performance statistics from its past users, allowing  a comparative analysis of LLMs’ performance against that of medical students and physicians. | / | English | https://openai.com |
|  | ChatGPT-4 |  |  |  |  |  |  |  |  |
| Miao J，2024^84^ | ChatGPT-3.5 | 975 | Obj. | MCQs | Nephrology test question bank | Nephrology Self-Assessment Program and Kidney  Self-Assessment Program | Nephrology | English | / |
|  | ChatGPT-4 |  |  |  |  |  |  |  |  |
|  | nephrologists and nephrology trainees |  |  |  |  |  |  |  |  |
| Kaneda Y，2023^85^ | ChatGPT-3.5 | 155 | Obj. | MCQs | Japanese national childcare worker examination | The Japanese national childcare worker examination is a test for obtaining a childcare worker qualification and consists of two parts: a written examination and a practical examination | Childcare | Japanese | / |
|  | ChatGPT-4 |  |  |  |  |  |  |  |  |
| Takagi S，2023^86^ | ChatGPT-3.5 | 254 | Obj. | MCQs | JMLE | The latest  JMLE, number 117, conducted on February 4 and 5, 2023, was  also used for this study. | / | Japanese | / |
|  | ChatGPT-4 |  |  |  |  |  |  |  |  |
|  | examinees |  |  |  |  |  |  |  |  |
| Ali R，2023^87^ | ChatGPT-3.5 | 149 | Obj. | MCQs | SANS | Self-Assessment Neurosurgery  Examination (SANS) Indications Examination, a 149-question module  designed specifically for oral boards preparation, focusing on surgical  indications, diagnostic testing, and interventional decision-making | Neurosurgery | English | / |
|  | ChatGPT-4 |  |  |  |  |  |  |  |  |
|  | Bard |  |  |  |  |  |  |  |  |
| Ohta K，2023^88^ | ChatGPT-3.5 | 185 | Obj. | MCQs | JNDE | Japanese National Dentist Examination | dentists | Japanese | / |
|  | ChatGPT-4 |  |  |  |  |  |  |  |  |
|  | Bard |  |  |  |  |  |  |  |  |
| Watari T，2023^89^ | ChatGPT-4 | 137 | Obj. | MCQs | GM-ITE | GM-ITE examination questions for the years 2020, 2021,  and 2022 | / | Japanese | / |
|  | People |  |  |  |  |  |  |  |  |
| Roos J，2023^90^ | ChatGPT-3.5 | 630 | Obj. | MCQs | the spring and fall  2022 German Medical State Examinations. | A total of 630 out  of 640 multiple-choice questions in German (including questions  containing media) were analyzed. | / | English | / |
|  | ChatGPT-4 |  |  |  |  |  |  |  |  |
|  | Bing chat |  |  |  |  |  |  |  |  |
|  | People |  |  |  |  |  |  |  |  |
| Guillen-Grima F，2023^91^ | ChatGPT-3.5 | 182 | Obj. | MCQs | MIR Exam | 2022 Spanish Medical Residency Entrance Exam | / | English and  Spanish | / |
|  | ChatGPT-4 |  |  |  |  |  |  |  |  |
| Huang RS，2023^92^ | ChatGPT-3.5 | 110 | Obj. | MCQs | The University of Toronto Department of Family and  Community Medicine Progress Test | An official University of  Toronto Department of Family and Community Medicine  Progress Test was used for this study, consisting of 110  questions. | / | English | / |
|  | ChatGPT-4 |  |  |  |  |  |  |  |  |
|  | Family Medicine residents |  |  |  |  |  |  |  |  |
| Schubert MC，2024^93^ | ChatGPT-3.5 | 1956 | Obj. | MCQs | an educational company | A question bank from an educational company with 2036 questions that resemble neurology board  questions1 | Neurology | English | / |
|  | ChatGPT-4 |  |  |  |  |  |  |  |  |
|  | Human |  |  |  |  |  |  |  |  |
| Torres-Zegarra BC，2023^94^ | ChatGPT-3.5 | 180 | Obj. | MCQs | P-NLME | Peruvian National Licensing Examination | / | English | / |
|  | ChatGPT-4 |  |  |  |  |  |  |  |  |
|  | Bard |  |  |  |  |  |  |  |  |
|  | Claude |  |  |  |  |  |  |  |  |
|  | Bing chat |  |  |  |  |  |  |  |  |
|  | Peruvian examinees |  |  |  |  |  |  |  |  |
| Kirshteyn G，2024^95^ | ChatGPT-3.5 | 70 | Obj. | MCQs | NBME and uWISE | the National Board of Medical Examiners(NBME) and the Association of Professors of Gynecology & Obstetrics (APGO) Web-Based Interactive Self-Evaluation (uWISE) | obstetrics and gynecology | English | / |
|  | LLaMA 2 70B |  |  |  |  |  |  |  |  |
| van Nuland M，2024^96^ | ChatGPT-3.5 | 264 | Obj. | MCQs | parate kennis | This database holds multiple choice questions (four answering options) used  to maintain a factual knowledge level within the field  of clinical pharmacy. | clinical pharmacy practice | Dutch and English | / |
|  | pharmacists |  |  |  |  |  |  |  |  |
| Danesh A，2024^97^ | ChatGPT-3.5 | 311 | Obj. | MCQs | 2023 in-service examination administered by the American Academy of Periodontology(AAP). | The dataset of in-service examination questions was accessed through Nova  Southeastern University’s Department of Periodontology. | Periodontology | English | / |
|  | ChatGPT-4 |  |  |  |  |  |  |  |  |
| Huang CY，2024^98^ | ChatGPT-3.5 | 24 | Obj. and Open | MCQs、MAQs and CBQs | ABD and journal Pediatric  Dermatology | American Board of Dermatology and the “Photoquiz” section of the journal Pediatric  Dermatology from issues published between July 2022 and July 2023 | Pediatric  Dermatology | English | / |
|  | ChatGPT-4 |  |  |  |  |  |  |  |  |
|  | clinicians |  |  |  |  |  |  |  |  |
| Fiedler B，2024^99^ | ChatGPT-3.5 | 98 | Obj. | MCQs | 2023 ASES MOC examination | 2023 American  Shoulder and Elbow Surgeons (ASES) Maintenance of Certification (MOC) self-assessment  exam. | Shoulder and Elbow Surgery | English | / |
|  | ChatGPT-4 |  |  |  |  |  |  |  |  |
|  | fellowship-trained surgeons |  |  |  |  |  |  |  |  |
| Coleman MC，2024^100^ | ChatGPT-3.5 | 495 | Obj. | MCQs | a database | All examination questions, which were delivered via a digital assessment platform (ExamSoft;  ExamSoft Worldwide LLC) to third-year veterinary students at the University of Georgia College  of Veterinary Medicine in both core and elective  courses during the 2022–2023 academic year, were  collected into a database. | veterinary surgeon | English | / |
| Abbas A，2024^101^ | ChatGPT-3.5 | 163 | Obj. | MCQs | NBME | The National Board of Medical Examiners (NBME) | / | English | / |
|  | ChatGPT-4 |  |  |  |  |  |  |  |  |
|  | Bard |  |  |  |  |  |  |  |  |
|  | Claude |  |  |  |  |  |  |  |  |
| Jarou ZJ，2024^102^ | ChatGPT-3.5 | 44 | Obj. | MCQs | PEERprep In-Training Self-Test and Study Guide. | the  American College of Emergency Physicians (ACEP)  PEERprep In-Training Self-Test and Study Guide. | emergency medicine | English | / |
|  | ChatGPT-4 |  |  |  |  |  |  |  |  |
|  | human test takers |  |  |  |  |  |  |  |  |
| Sensoy E，2024^103^ | ChatGPT-3.5 | 41 | Obj. | MCQs | the American Academy of Ophthalmology 2022–2023 Basic and Clinical  Science Course External Disease and Cornea book | / | ophthalmologic | English | / |
|  | Bard |  |  |  |  |  |  |  |  |
|  | Bing chat |  |  |  |  |  |  |  |  |
| Guerra GA，2024^104^ | ChatGPT-3.5 | 420 | Obj. | MCQs | OITE | Orthopaedic In-Training Examination question sets from 2021 and 2022 were compiled | orthopedics | English | / |
|  | Bard |  |  |  |  |  |  |  |  |
| Agarwal M，2023^105^ | Bing chat | 55 | Obj. | MCQs | a curated set of MCQs in medical physiology | The MCQs were created following the CBME guidelines provided by the NMC | medical physiology | English | / |
|  | ChatGPT-3.5 |  |  |  |  |  |  |  |  |
|  | Claude 2 |  |  |  |  |  |  |  |  |
| Cheong KX，2024^106^ | chatGPT-3.5 | 45 | open | / | three categories were assessed: AMD, DR and others | these questions were separate and distinct from the paired question-and-answer training dataset that was originally used to train OcularBERT.22 These questions covered risk factors, clinical presentation, diagnosis, treatment and prevention and prognosis | ophthalmology | English | / |
|  | chatGPT-4 |  |  |  |  |  |  |  | / |
|  | Bard |  |  |  |  |  |  |  | / |
|  | OcularBERT |  |  |  |  |  |  |  | / |
| Zhou S，2024^107^ | chatGPT-3.5 | 150 | open | / | NCCN guidelines for colon and rectal cancer | These domains encompassed the principles of imaging, principles of pathology and molecular review, principles of surgery, treatment of nonmetastatic colon cancer, treatment of nonmetastatic rectal cancer, and management of metastatic CRC | colorectal cancer | English and Chinese | https://openai.com/blog/chatgpt |
|  | chatGPT-4 |  |  |  |  |  |  |  | https://openai.com/blog/chatgpt |
|  | Doctor GPT |  |  |  |  |  |  |  | https://openai.com/blog/chatgpt |
|  | Llama 2 70B |  |  |  |  |  |  |  | / |
|  | Mixtral-8x7B |  |  |  |  |  |  |  | / |
|  | Bard |  |  |  |  |  |  |  | / |
|  | Claude 2.1 |  |  |  |  |  |  |  | / |
| Kozaily E，2024^108^ | chatGPT-3.5 | 30 | open | / | American Heart Association/American College of Cardiology/Heart Failure Society of America (AHA/ACC/HFSA) heart failure guidelines | with the default settings of the chatbots interface, each question was asked in a separate chat window. Then, each set of responses was assessed by two board certified HF cardiologists. | cardiology | English | https://chat.openai.com/ |
|  | Bard |  |  |  |  |  |  |  | https://bard.google.com/ |
| Xia S，2024^109^ | chatGPT-3.5 | 145 | open | / | experts specialized in thyroid disease according to the patients’ history | / | Endocrinology | English | / |
|  | New Bing chat |  |  |  |  |  |  |  | / |
|  | Junior doctors |  |  |  |  |  |  |  | / |
| Lee Y，2024^110^ | chatGPT-4 | 36 | open | clinical vignettes | 26 preexisting bariatric and metabolic guidelines | we segmented questions into distinct categories as follows: bariatric surgery techniques, complications, weight recurrence, special populations, perioperative management, nutrition, and concurrent surgery | bariatric surgery | English | / |
|  | Bard |  |  |  |  |  |  |  | / |
|  | Bing chat |  |  |  |  |  |  |  | / |
| Doğan L，2024^111^ | chatGPT-3.5 | 25 | open | / | AAPOS website | These questions included the definition of amblyopia and its causes, treatment methods, management of treatment, and prognosis | ophthalmology | English | / |
|  | Bing chat |  |  |  |  |  |  |  | / |
|  | PaLM2 |  |  |  |  |  |  |  | / |
| Lee TJ，2024^112^ | chatGPT-3.5 | 208 | open | clinical vignettes | 2017 American College of Cardiology’s | / | cardiology | English | / |
|  | Gemini |  |  |  |  |  |  |  | / |
| Lang SP，2024^113^ | chatGPT-3.5 | 50 | open | / | google | comprised the most critical and commonly addressed patient concerns on lumbar fusion surgery | spinal surgery | English | https://chat.openai.com/chat |
|  | Bard |  |  |  |  |  |  |  | https://bard.google.com/chat |
| Iannantuono GM，2024^114^ | chatGPT-3.5 | 60 | open | / | clinical experience | we generated 60 open-ended questions based on our clinical experience covering 4 different domains of IO including “mechanisms” (of action)，“indications” (for use), “toxicities,” and “prognosis”. | oncology | English | / |
|  | chatGPT-4 |  |  |  |  |  |  |  | / |
|  | Bard |  |  |  |  |  |  |  | / |
| Anguita R，2024^115^ | chatGPT-3.5 | 27 | open | clinical vignettes | a set of questions commonly encountered in our clinical practice managing patients with malignant melanoma of the choroid, as well as questions from the clinic email inbox monitored by the specialist ocular oncology nurses | the set of questions was classified into two categories: medical advice with 12 queries and pre- and post-operative questions with 15 queries | choroidal melanoma | English | / |
|  | Bing chat |  |  |  |  |  |  |  | / |
|  | Docs-GPT Beta |  |  |  |  |  |  |  | / |
| Zhang Y，2024^116^ | chatGPT-3.5 | 45 | open | / | we collected FAQs about BPH from some of the world's authoritative professional associations and renowned medical and device institutions | these questions covered a range of topics, including the definition of BPH, pathologic features, risk factors, epidemiologic features, clinical manifestations, laboratory tests, diagnosis, and treatment. | benign prostatic hyperplasia frequently asked | English | / |
|  | chatGPT-4 |  |  |  |  |  |  |  | / |
|  | New Bing chat |  |  |  |  |  |  |  | / |
| Xue E，2024^117^ | chatGPT-3.5 | 72 | open | clinical scenarios | the 7th edition European Bone Marrow Transplantation Handbook, Lexicomp, and BeTheMatch | with 3 levels of difficulty ranging from “easy,” testing superficial factual knowledge, to “difficult,” testing complex clinical scenarios | Orthopedics and Spinal Surgery | English | / |
|  | chatGPT-4 |  |  |  |  |  |  |  | / |
|  | Bard |  |  |  |  |  |  |  | / |
| Cao JJ，2024^118^ | chatGPT-3.5 | 20 | open | / | the American College of Radiology Liver Imaging Reporting and Data System (LI-RADS) and American Association for the Study of Liver Disease guidelines for hepatocellular carcinomaoncologist, and one hepatologist | these questions were inspired by real questions the panel encountered from patients and multidisciplinary tumor boards, with an emphasis on the American College of Radiology Liver Imaging Reporting and Data System (LI-RADS) and American Association for the Study of Liver Disease guidelines for hepatocellular carcinoma | liver cancer | English | / |
|  | Gemini |  |  |  |  |  |  |  | / |
|  | Bing chat |  |  |  |  |  |  |  | / |
| Monroe CL，2024^119^ | chatGPT-3.5 | 28 | open | clinical vignettes | common terminology used in radiology reports and cardiac imaging guidelines | / | cardiac imaging | English | / |
|  | chatGPT-4 |  |  |  |  |  |  |  | / |
| Chervonski E，2024^120^ | chatGPT-3.5 | 24 | open | / | three questions were drawn from the “Common Questions” subsection for Patients and Referring Physicians on the Society for Vascular Surgery website. The other 21 questions included commonly asked questions written by three vascular surgery attendings at different institutions | these questions focused on seven disease domains: peripheral artery disease, abdominal aortic aneurysms, carotid artery disease, deep venous thrombosis, varicose veins, dialysis access for chronic kidney disease, and thoracic outlet syndrome. Within each domain, a question on symptoms, diagnosis, and treatment was asked | hematology department | English | / |
|  | PaLM2 |  |  |  |  |  |  |  | / |
| Kassab J，2024^121^ | chatGPT-4 | 45 | open | / | addressing screening guidelines, preventive strategies, and management of common diseases | 45 derived from the USPTF grade A, B recommendations,21 6 from the CDC,22 and 5 on the management of commonly encountered diseases in the primary care setting | orthopedics and spinal surgery | English | https://openai.com/gpt-4 |
|  | Bard |  |  |  |  |  |  |  | https://bard.google.com |
| Al-Sharif EM，2024^122^ | chatGPT-3.5 | 112 | open | / | Electronic Medical Record System | / | oculoplastic patient | English | / |
|  | Bard |  |  |  |  |  |  |  | / |
| Mejia MR，2024^123^ | chatGPT-3.5 | 29 | open | / | NASS guidelines | / | lumbar disc herniation | English | / |
|  | chatGPT-4 |  |  |  |  |  |  |  | / |
| Lee TJ，2024^124^ | chatGPT-3.5 | 75 | open | clinical vignettes | the 2022 Cleveland Clinic | / | hyperlipidemia | English | / |
|  | chatGPT-4 |  |  |  |  |  |  |  | / |
| Oliveira AL，2024^125^ | chatGPT-3.5 | 80 | open | open-ended，introductory contextualization component | patients, prior to surgery, during the perioerative period or at reassessment appointments | the format for each question includes an introductory contextualization component | parkinson's disease | English | / |
|  | chatGPT-4 |  |  |  |  |  |  |  | / |
| Lim ZW，2023^126^ | chatGPT-3.5 | 31 | open | / | National Eye Institute, the American Academy of Ophthalmology, and the Brien Holden Vision Institute | questions were categorised into 6 domains—pathogenesis, risk factors, clinical presentation, diagnosis, treatment and prevention, and prognosis | myopia care | English | / |
|  | chatGPT-4 |  |  |  |  |  |  |  | / |
|  | Bard |  |  |  |  |  |  |  | / |
| Rahsepar AA，2023^127^ | chatGPT-3.5 | 120 | open | / | Lung Imaging Reporting and Data System (Lung-RADS) version 2022 from the American College of Radiology and the Fleischner Society | / | Lung Cancer | English | http://openai.com |
|  | Bard |  |  |  |  |  |  |  | http://openai.com |
|  | Bing chat |  |  |  |  |  |  |  | / |
| Pushpanathan K，2023^128^ | chatGPT-3.5 | 37 | open | / | reputable online health information sites (e.g., National Eye Institute, American Academy of Ophthalmology) | selecting those commonly encountered in a clinical setting, ensuring the relevance and practicality of the inquiries | ophthalmology | English | / |
|  | chatGPT-4 |  |  |  |  |  |  |  | / |
|  | Bard |  |  |  |  |  |  |  | / |
| Coskun BN，2024^129^ | chatGPT-4 | 23 | open | / | / | focus on three core categories for the use of MTX in the treatment of rheumatoid arthritis: mechanism of action , potential side effects , and lifestyle-related concerns | methotrexate use | English | / |
|  | Bard |  |  |  |  |  |  |  | / |
|  | Bing chat |  |  |  |  |  |  |  | / |
| King RC，2024^130^ | chatGPT-3.5 | 83 | open | / | websites for professional medical societies and institutions | Of these questions, 56 addressed general amyloidosis topics, while 42 were specific to cardiology, gastroenterology, and neurology. | / | English | https://openai.com/blog/ |
|  | chatGPT-4 | 98 |  |  |  |  |  |  |  |
| Pinto VBP，2024^131^ | chatGPT-3.5 | 20 | open | conceptual and case-based | three urologists and The Incontinence After Prostate Treatment: AUA/SUFU Guideline | the guideline provides a list of statements divided in different domains: (a) Preprostate treatment, (b) postprostate treatment, (c) evaluation of incontinence after prostate treatment, (d) treatment options, (e) complications after surgery and (f) special situations, including a poor quality urethra, bladder neck stricture and the management of complications of surgical treatment for PPUI | postprostatectomy urinary incontinence | English | / |
|  | chatGPT-4 |  |  |  |  |  |  |  | / |
| Momenaei B，2024^132^ | chatGPT-3.5 | 88 | open | Author | / | we created sets of 88 typical inquiries covering diverse aspects, such as definition, visual implications, diagnostic approaches, management, postoperative details, complications associated with retinal detachment (RD, 39 questions); macular hole (MH, 25 questions); and epiretinal membrane (ERM, 24 questions) | ophthalmology | English | / |
|  | chatGPT-4 |  |  |  |  |  |  |  | https://openai.com/gpt-4 |
| Stevenson E，2024^133^ | chatGPT-3.5 | 15 | open | Author | fictional cases | / | thyroid function test | English | / |
|  | Bard |  |  |  |  |  |  |  | / |
| Dronkers EAC，2024^134^ | chatGPT-4 | 20 | open | clinical case | / | / | Acquired Bilateral Vocal Fold Paralysis | English | / |
|  | Llama 2 |  |  |  |  |  |  |  | / |
| Rahimli Ocakoglu S，2024^135^ | chatGPT-4 | 15 | open | / | RCOG website | These questions were classified as “definition” (questions 1–4), “diagnostic” (questions 5–7), and “treatment” (questions 8–15). | Pelvic Organ Prolapse | English | / |
|  | Bard |  |  |  |  |  |  |  | / |
|  | Bing |  |  |  |  |  |  |  | / |
| Gandhi AP，2024^136^ | chatGPT-3.5 | 100 | open | / | secondary data conducted in February 2023 at a publicly funded medical college in Hyderabad, India | the Final Year–Part I program comprises three subjects: otorhinolaryngology, ophthalmology, and community medicine. | India Undergraduate Community Medicine Examination | English | https://openai.com/blog/chatgpt/ |
|  | students |  |  |  |  |  |  |  | / |
| Tariq R，2024^137^ | chatGPT-3.5 | 47 | open | simulated scenarios | / | / | colonoscopy | English | https://openai.com/blog/chatgpt |
|  | chatGPT-4 |  |  |  |  |  |  |  | / |
|  | Bard |  |  |  |  |  |  |  | / |
| Li P，2024^138^ | chatGPT-3.5 | 68 | open | / | 2022 European Society of Cardiology guideline | / | cardio-oncology | English | / |
|  | chatGPT-4 |  |  |  |  |  |  |  | / |
|  | Bard |  |  |  |  |  |  |  | / |
|  | Llama 2 |  |  |  |  |  |  |  | / |
|  | Claude 2 |  |  |  |  |  |  |  | / |
| Sosa BR，2024^139^ | chatGPT-4 | 15 | open | clinical vignettes | / | / | orthopedics | English | / |
|  | Bard |  |  |  |  |  |  |  | / |
|  | Bing chat |  |  |  |  |  |  |  | / |
| Shukla R，2024^140^ | chatGPT-3.5 | 10 | top1 | clinical case | Neuro-Ophthalmology 2023: When Should I Worry? Concerning Signs, Symptoms, and Findings in Neuro-Ophthalmology. | / | neuro-ophthalmology | English | / |
|  | Bing chat |  |  |  |  |  |  |  | / |
|  | Gemini |  |  |  |  |  |  |  | / |
| Koga S，2024^141^ | chatGPT-3.5 | 25 | top1 | clinical case | Mayo Clinic brain bank clinicopathological conferences | / | neurology | English | / |
|  | chatGPT-4 |  |  |  |  |  |  |  | / |
|  | Bard |  |  |  |  |  |  |  | / |
| Warrier A，2024^142^ | chatGPT-3.5 | 100 | top1 | clinical vignettes | the second edition of *Otolaryngology Cases—The University of Cincinnati Clinical Portfolio* | detailed patient histories were selected as clinical vignettes of 10 different otolaryngology subcategories | otolaryngology | English | / |
|  | chatGPT-4 |  |  |  |  |  |  |  | https://openai.com/  gpt-4 |
|  | PaLM2 |  |  |  |  |  |  |  | / |
|  | Bing chat |  |  |  |  |  |  |  | / |
| Kumar RP，2024^143^ | chatGPT-3.5 | 20 | top1、top5 | clinical vignettes | Dynamed | Dynamed is a point-of-care resource for physicians offering a detailed disease overview. | neurosurgery | English | / |
|  | chatGPT-4 |  |  |  |  |  |  |  | / |
|  | Bard |  |  |  |  |  |  |  | / |
|  | Perplexity |  |  |  |  |  |  |  | / |
| Hirosawa T，2024^144^ | ChatGPT-4 | 392 | top1 | case reports | American Journal of Case Reports | This peer-reviewed scientific journal publishes original, often complex, case reports spanning a variety of medical fields. | / | English | https://chat.openai.com/ |
|  | Gemini |  |  |  |  |  |  |  | https://gemini.google.com/app |
|  | Llama 2 |  |  |  |  |  |  |  | https://llama2.ai/ |
| Mandalos A，2024^145^ | chatGPT-3.5 | 11 | top1 | scenarios | General Hospital of Karditsa,  Karditsa, Greece | / | ophthalmology | English | / |
|  | Copilot |  |  |  |  |  |  |  | / |
|  | Gemini |  |  |  |  |  |  |  | / |
|  | ophthalmic specialists |  |  |  |  |  |  |  | / |
| Krusche M，2024^146^ | chatGPT-4 | 132 | top1 | clinical vignettes | the data set of Gräf et al. | / | rheumatology | English | https://chat.openai.com/share/7d1ea5ff-2a17-4aad-bf62-75d610173efd |
|  | rheumatologists |  |  |  |  |  |  |  | / |
| Delsoz M，2024^147^ | chatGPT-3.5 | 20 | top1 | clinical vignettes | Department of Ophthalmology and Visual Sciences at the University of Iowa | / | ophthalmology | English | https://openai.com/  blog/chatgpt. |
|  | chatGPT-4 |  |  |  |  |  |  |  | https://openai.com/  blog/chatgpt. |
|  | corneal specialists |  |  |  |  |  |  |  | / |
| Kozel G，2024^148^ | chatGPT-3.5 | 20 | top1 | case details | medical literature | / | brain tumors | English | / |
|  | chatGPT-4 |  |  |  |  |  |  |  | / |
| Apornvirat S，2024^149^ | chatGPT-4 | 36 | top1 | clinical vignettes | emergency clinic at a single UK dermatology department | / | dermatology | English | / |
|  | dermatologists |  |  |  |  |  |  |  | / |
| Albaladejo A，2024^150^ | chatGPT-4 | 12 | top1 | clinical case | Printemps de la Médecine Interne | / | internal medicine | French | https://openai.com/blog/chatgpt |
|  | Bard |  |  |  |  |  |  |  | https://bard.google.com/ |
|  | internist experts |  |  |  |  |  |  |  | / |
| Zandi R，2024^151^ | chatGPT-4 | 80 | Top1、Triage | scenarios | designed by author | systematically constructed common scenarios encountered in ophthalmology  from the perspective of a patient. | ophthalmology | English | / |
|  | Bard |  |  |  |  |  |  |  | / |
| Hirosawa T，2023^152^ | chatGPT-3.5 | 52 | top1、top5 | clinical vignettes | PubMed | / | internal medicine | English | / |
|  | chatGPT-4 |  |  |  |  |  |  |  | / |
|  | General Internal Medicine physicians |  |  |  |  |  |  |  | / |
| Hirosawa T，2023^153^ | chatGPT-3.5 | 30 | top1、top5 | clinical vignettes | designed by author | / | / | English | https://chat.openai.com/chat |
|  | physicians |  |  |  |  |  |  |  | / |
| Fraser H，2023^154^ | chatGPT-3.5 | 37 | top1、top5、triage | symptom data | Rhode Island Hospital emergency department | / | / | English | / |
|  | chatGPT-4 |  |  |  |  |  |  |  | / |
|  | WebMD |  |  |  |  |  |  |  | https://symptoms.webmd.com/default.htm#/info |
|  | Ada Health |  |  |  |  |  |  |  | https://ada.com/ |
|  | people |  |  |  |  |  |  |  | / |
| Rojas-Carabali W，2024^155^ | chatGPT-3.5 | 25 | top1 | standard cases | designed by author | / | / | English | https://openai.com/  blog/chatgpt/ |
|  | chatGPT-4 |  |  |  |  |  |  |  | https://openai.com/  blog/chatgpt/ |
|  | ophthalmologists |  |  |  |  |  |  |  | / |
| Gräf M，2022^156^ | Ada Health | 132 | top1 | clinical vignettes | Evaluation of Triage Tools in Rheumatology dataset | bETTeR is an investigator-initiated multi-center, randomized controlled trial that recruited 600 patients newly presenting to three rheumatology outpatient clinics in Germany | rheumatology | English | www.ada.com |
|  | physicians |  |  |  |  |  |  |  | / |
| Ward M，2024^157^ | chatGPT-3.5 | 27 | top1 | scenarios | designed by author | / | neurosurgery | English | / |
|  | chatGPT-4 |  |  |  |  |  |  |  | / |
|  | neurosurgical attendings |  |  |  |  |  |  |  | / |
| Hirosawa T，2023^158^ | Bard | 82 | top1、top5 | vignettes | designed by author | / | / | English | https://bard.google.com/ |
|  | physicians |  |  |  |  |  |  |  | / |
| Lyons RJ，2024^159^ | chatGPT-4 | 44 | top3、triage | clinical vignettest | a literature review  of common emergency room ophthalmologic diagnoses | / | Ophthalmology | English | / |
|  | Bing chat |  |  |  |  |  |  |  | / |
|  | WebMD |  |  |  |  |  |  |  | / |
|  | Ophthalmology trainees |  |  |  |  |  |  |  | / |
| Makhoul M，2024^160^ | chatGPT-3.5 | 32 | top3 | case scenarios | designed by author | The principal investigator hand-picked 8 archived  patient cases for each of the four main ENT symptoms | ear, nose, and throat (ENT) | English | https://symptomchecker.io |
|  | ENT  physicians, ENT residents, FM specialists, Med2, Med3 |  |  |  |  |  |  |  | / |
| Shemer A，2024^161^ | chatGPT-3.5 | 63 | top3 | clinical cases | designed by author | include all adult patients (age>18 years) who were referred to the emergency  department and admitted to the ophthalmology department  at one tertiary medical center, from June 2022 to January  2023. | ophthalmology | English | https://openai.  com/blog/chatgpt |
|  | residents and attendings |  |  |  |  |  |  |  | / |
| Gunes YC，2024^162^ | chatGPT-3.5 | 124 | top 5 |  | Thoracic Society of Radiology | Thoracic Society of Radiology has published monthly cases in the “Case of the Month”category on its website (https://thoracicrad.org) | radiology | English | https://chat.openai.com |
|  | chatGPT-4 |  |  |  |  |  |  |  | https://chat.openai.com |
|  | Bard |  |  |  |  |  |  |  | https://bard.google.com |
|  | Gemini |  |  |  |  |  |  |  | https://gemini.google.com |
|  | Gemini 1.5 pro |  |  |  |  |  |  |  | https://deepmind.google/technologies/gemini/  #gemini-1.5 |
|  | Llama 3 70b |  |  |  |  |  |  |  | https://llama.meta.com/  llama3 |
|  | Bing chat |  |  |  |  |  |  |  | https://www.bing.com/ |
|  | Copilot |  |  |  |  |  |  |  | https://copilot.microsoft.com |
|  | Perplexity |  |  |  |  |  |  |  | https://www.perplexity.ai |
|  | Claude 3 Opus |  |  |  |  |  |  |  | https://www.anthropic.com/claude |
|  | Radiologist |  |  |  |  |  |  |  |  |
| Sarangi PK，2023^163^ | chatGPT-3.5 | 75 | top 5 |  | a textbook and an online platform | cardiovascular and thoracic imaging patterns sourced from a textbook (Chapman & Nakielny’s Aids to Radiological Differential Diagnosis) and an online platform https://radiopaedia.org | cardiology and thoracic surgery | English | https://chat.openai.com |
|  | Bard |  |  |  |  |  |  |  | https://bard.google.com |
|  | Bing chat |  |  |  |  |  |  |  | https://www.bing.com/ |
|  | Perplexity |  |  |  |  |  |  |  | https://www.perplexity.ai |
| Berg HT，2024^164^ | chatGPT-3.5 | 30 | top 5 |  | Jeroen Bosch Hospital’ hospital | / | / | English |  |
|  | chatGPT-4 |  |  |  |  |  |  |  |  |
|  | physicians |  |  |  |  |  |  |  |  |
| Haider SA，2024^165^ | chatGPT-4 | 50 | Triage | clinical vignettes | five well-established breast disease classification systems | include: Baker classification for capsular contracture; Fischer classification for gender-affirming mastectomy; Kajava classification for ectopic breast tissue; Regnault classification for breast ptosis, University of Texas Southwestern Medical Center (UTSW) classification for grading gynecomastia | breast diseases | English | / |
|  | Gemini |  |  |  |  |  |  |  | / |
| Pressman SM，2024^166^ | chatGPT-4 | 136 | Triage | scenarios | designed by author | each prompt included a fictionalized vignette and a specific hand injury diagnosis | / | English | https://chat.openai.com/chat |
|  | Gemini |  |  |  |  |  |  |  | https://gemini.google.com/app |
| Gan RK，2024^167^ | chatGPT-3.5 | 15 | Triage | questionnaire | designed by author | the questionnaire's 15 scenarios were expertly crafted by Emergency Medical Services (EMS) Medical Directors and Emergency Faculty affiliated with the University of North Carolina School of Medicine. The scenarios were carefully selected to ensure diverse triage levels and START criteria adherence. | / | English | https://help.openai.com/en/articles/6825453-chatgptrelease-notes |
|  | PaLM2 |  |  |  |  |  |  |  | https://blog.google/products/bard/google-bardnew-features-update-july-2023/ |
|  | people |  |  |  |  |  |  |  | / |
| Aiumtrakul N，2024^168^ | chatGPT-3.5 | 539 | Triage | / | / | use the Mayo Clinic Oxalate Diet Handbook as the reference to determine the  oxalate content of 539 different foods | / | English | https://chat.openai.com/ |
|  | chatGPT-4 |  |  |  |  |  |  |  | https://openai.com/gpt-4 |
|  | Bard |  |  |  |  |  |  |  | https://blog.google/technology/ai/bard-google-aisearch-updates/ |
|  | Bing AI |  |  |  |  |  |  |  | https://www.microsoft.com/en-us/edge/features/bing-chat?form=MT00D8 |

References:

1. Tsoutsanis P, Tsoutsanis A. Evaluation of Large language model performance on the Multi-Specialty Recruitment Assessment (MSRA) exam. Comput Biol Med. 2024;168:107794.
2. Long C, Subburam D, Lowe K, et al. ChatENT: Augmented Large Language Model for Expert Knowledge Retrieval in Otolaryngology-Head and Neck Surgery. Otolaryngol Head Neck Surg. 2024;171(4):1042-1051.
3. Tao BK, Hua N, Milkovich J, Micieli JA. ChatGPT-3.5 and Bing Chat in ophthalmology: an updated evaluation of performance, readability, and informative sources. Eye (Lond). 2024;38(10):1897-1902.
4. Shieh A, Tran B, He G, Kumar M, Freed JA, Majety P. Assessing ChatGPT 4.0's test performance and clinical diagnostic accuracy on USMLE STEP 2 CK and clinical case reports. Sci Rep. 2024;14(1):9330.
5. Sarangi PK, Narayan RK, Mohakud S, Vats A, Sahani D, Mondal H. Assessing the Capability of ChatGPT, Google Bard, and Microsoft Bing in Solving Radiology Case Vignettes. Indian J Radiol Imaging. 2023;34(2):276-282.
6. Singer MB, Fu JJ, Chow J, Teng CC. Development and Evaluation of Aeyeconsult: A Novel Ophthalmology Chatbot Leveraging Verified Textbook Knowledge and GPT-4. J Surg Educ. 2024;81(3):438-443.
7. Hanna RE, Smith LR, Mhaskar R, Hanna K. Performance of Language Models on the Family Medicine In-Training Exam. Fam Med. Published online August 12, 2024.
8. Kadoya N, Arai K, Tanaka S, et al. Assessing knowledge about medical physics in language-generative AI with large language model: using the medical physicist exam. Radiol Phys Technol. Published online September 10, 2024.
9. Sallam M, Al-Mahzoum K, Almutawaa RA, et al. The performance of OpenAI ChatGPT-4 and Google Gemini in virology multiple-choice questions: a comparative analysis of English and Arabic responses. BMC Res Notes. 2024;17(1):247.
10. Gravina AG, Pellegrino R, Palladino G, Imperio G, Ventura A, Federico A. Charting new AI education in gastroenterology: Cross-sectional evaluation of ChatGPT and perplexity AI in medical residency exam. Dig Liver Dis. 2024;56(8):1304-1311.
11. Passby L, Jenko N, Wernham A. Performance of ChatGPT on Specialty Certificate Examination in Dermatology multiple-choice questions. Clin Exp Dermatol. 2024;49(7):722-727.
12. Sabri H, Saleh MHA, Hazrati P, et al. Performance of three artificial intelligence (AI)-based large language models in standardized testing; implications for AI-assisted dental education. J Periodontal Res. Published online July 18, 2024.
13. Çamur E, Cesur T, Güneş YC. Can large language models be new supportive tools in coronary computed tomography angiography reporting?. Clin Imaging. 2024;114:110271.
14. Lubitz M, Latario L. Performance of Two Artificial Intelligence Generative Language Models on the Orthopaedic In-Training Examination. Orthopedics. 2024;47(3):e146-e150.
15. Gupta R, Hamid AM, Jhaveri M, Patel N, Suthar PP. Comparative Evaluation of AI Models Such as ChatGPT 3.5, ChatGPT 4.0, and Google Gemini in Neuroradiology Diagnostics. Cureus. 2024;16(8):e67766.
16. Lee GU, Hong DY, Kim SY, et al. Comparison of the problem-solving performance of ChatGPT-3.5, ChatGPT-4, Bing Chat, and Bard for the Korean emergency medicine board examination question bank. Medicine (Baltimore). 2024;103(9):e37325.
17. Is EE, Menekseoglu AK. Comparative performance of artificial intelligence models in rheumatology board-level questions: evaluating Google Gemini and ChatGPT-4o. Clin Rheumatol. Published online September 28, 2024.
18. D'Anna G, Van Cauter S, Thurnher M, Van Goethem J, Haller S. Can large language models pass official high-grade exams of the European Society of Neuroradiology courses? A direct comparison between OpenAI chatGPT 3.5, OpenAI GPT4 and Google Bard. Neuroradiology. 2024;66(8):1245-1250.
19. Altamimi I, Alhumimidi A, Alshehri S, et al. The scientific knowledge of three large language models in cardiology: multiple-choice questions examination-based performance. Ann Med Surg (Lond). 2024;86(6):3261-3266.
20. Lee Y, Tessier L, Brar K, et al. Performance of artificial intelligence in bariatric surgery: comparative analysis of ChatGPT-4, Bing, and Bard in the American Society for Metabolic and Bariatric Surgery textbook of bariatric surgery questions. Surg Obes Relat Dis. 2024;20(7):609-613.
21. Schoch J, Schmelz HU, Strauch A, Borgmann H, Nestler T. Performance of ChatGPT-3.5 and ChatGPT-4 on the European Board of Urology (EBU) exams: a comparative analysis. World J Urol. 2024;42(1):445. Published 2024 Jul 26.
22. May M, Körner-Riffard K, Kollitsch L, et al. Evaluating the Efficacy of AI Chatbots as Tutors in Urology: A Comparative Analysis of Responses to the 2022 In-Service Assessment of the European Board of Urology. Urol Int. 2024;108(4):359-366.
23. Sadeq MA, Ghorab RMF, Ashry MH, et al. AI chatbots show promise but limitations on UK medical exam questions: a comparative performance study. Sci Rep. 2024;14(1):18859.
24. Khalpey Z, Kumar U, King N, Abraham A, Khalpey AH. Large Language Models Take on Cardiothoracic Surgery: A Comparative Analysis of the Performance of Four Models on American Board of Thoracic Surgery Exam Questions in 2023. Cureus. 2024;16(7):e65083.
25. Patel EA, Fleischer L, Filip P, et al. Comparative Performance of ChatGPT 3.5 and GPT4 on Rhinology Standardized Board Examination Questions. OTO Open. 2024;8(2):e164.
26. Irmici G, Cozzi A, Della Pepa G, et al. How do large language models answer breast cancer quiz questions? A comparative study of GPT-3.5, GPT-4 and Google Gemini. Radiol Med. Published online August 13, 2024.
27. Kollitsch L, Eredics K, Marszalek M, et al. How does artificial intelligence master urological board examinations? A comparative analysis of different Large Language Models' accuracy and reliability in the 2022 In-Service Assessment of the European Board of Urology. World J Urol. 2024;42(1):20.
28. Morreel S, Verhoeven V, Mathysen D. Microsoft Bing outperforms five other generative artificial intelligence chatbots in the Antwerp University multiple choice medical license exam. PLOS Digit Health. 2024;3(2):e0000349.
29. Bajčetić M, Mirčić A, Rakočević J, Đoković D, Milutinović K, Zaletel I. Comparing the performance of artificial intelligence learning models to medical students in solving histology and embryology multiple choice questions. Ann Anat. 2024;254:152261.
30. Canillas Del Rey F, Canillas Arias M. Exploring the potential of Artificial Intelligence in Traumatology: Conversational answers to specific questions. Explorando el potencial de la inteligencia artificial en traumatología: respuestas conversacionales a preguntas específicas. Rev Esp Cir Ortop Traumatol.
31. Meyer A, Riese J, Streichert T. Comparison of the Performance of GPT-3.5 and GPT-4 With That of Medical Students on the Written German Medical Licensing Examination: Observational Study. JMIR Med Educ. 2024;10:e50965.
32. Toyama Y, Harigai A, Abe M, et al. Performance evaluation of ChatGPT, GPT-4, and Bard on the official board examination of the Japan Radiology Society. Jpn J Radiol. 2024;42(2):201-207.
33. Touma NJ, Caterini J, Liblk K. Performance of artificial intelligence on a simulated Canadian urology board exam: Is CHATGPT ready for primetime?. Can Urol Assoc J. Published online June 10, 2024.
34. Chan J, Dong T, Angelini GD. The performance of large language models in intercollegiate Membership of the Royal College of Surgeons examination. Ann R Coll Surg Engl. Published online March 6, 2024.
35. Patil NS, Huang RS, van der Pol CB, Larocque N. Comparative Performance of ChatGPT and Bard in a Text-Based Radiology Knowledge Assessment. Can Assoc Radiol J. 2024;75(2):344-350.
36. Hubany SS, Scala FD, Hashemi K, et al. ChatGPT-4 Surpasses Residents: A Study of Artificial Intelligence Competency in Plastic Surgery In-service Examinations and Its Advancements from ChatGPT-3.5. Plast Reconstr Surg Glob Open. 2024;12(9):e6136.
37. Vaishya R, Iyengar KP, Patralekh MK, et al. Effectiveness of AI-powered Chatbots in responding to orthopaedic postgraduate exam questions-an observational study. Int Orthop. 2024;48(8):1963-1969.
38. Nakajima N, Fujimori T, Furuya M, et al. A Comparison Between GPT-3.5, GPT-4, and GPT-4V: Can the Large Language Model (ChatGPT) Pass the Japanese Board of Orthopaedic Surgery Examination?. Cureus. 2024;16(3):e56402.
39. Thibaut G, Dabbagh A, Liverneaux P. Does Google's Bard Chatbot perform better than ChatGPT on the European hand surgery exam?. Int Orthop. 2024;48(1):151-158.
40. Lum ZC, Collins DP, Dennison S, et al. Generative Artificial Intelligence Performs at a Second-Year Orthopedic Resident Level. Cureus. 2024;16(3):e56104.
41. Menekşeoğlu AK, İş EE. Comparative performance of artificial ıntelligence models in physical medicine and rehabilitation board-level questions. Rev Assoc Med Bras (1992). 2024;70(7):e20240241.
42. Cheong RCT, Pang KP, Unadkat S, et al. Performance of artificial intelligence chatbots in sleep medicine certification board exams: ChatGPT versus Google Bard. Eur Arch Otorhinolaryngol. 2024;281(4):2137-2143.
43. Mesnard B, Schirmann A, Branchereau J, et al. Artificial Intelligence: Ready To Pass the European Board Examinations in Urology?. Eur Urol Open Sci. 2024;60:44-46.
44. Ming S, Guo Q, Cheng W, Lei B. Influence of Model Evolution and System Roles on ChatGPT's Performance in Chinese Medical Licensing Exams: Comparative Study. JMIR Med Educ. 2024;10:e52784.
45. Chow R, Hasan S, Zheng A, et al. The Accuracy of Artificial Intelligence ChatGPT in Oncology Examination Questions. J Am Coll Radiol. Published online August 2, 2024.
46. Kim SE, Lee JH, Choi BS, Han HS, Lee MC, Ro DH. Performance of ChatGPT on Solving Orthopedic Board-Style Questions: A Comparative Analysis of ChatGPT 3.5 and ChatGPT 4. Clin Orthop Surg. 2024;16(4):669-673.
47. Oura T, Tatekawa H, Horiuchi D, et al. Diagnostic accuracy of vision-language models on Japanese diagnostic radiology, nuclear medicine, and interventional radiology specialty board examinations. Jpn J Radiol. Published online July 20, 2024.
48. Lewandowski M, Łukowicz P, Świetlik D, Barańska-Rybak W. ChatGPT-3.5 and ChatGPT-4 dermatological knowledge level based on the Specialty Certificate Examination in Dermatology. Clin Exp Dermatol. 2024;49(7):686-691.
49. Knoedler L, Alfertshofer M, Knoedler S, et al. Pure Wisdom or Potemkin Villages? A Comparison of ChatGPT 3.5 and ChatGPT 4 on USMLE Step 3 Style Questions: Quantitative Analysis. JMIR Med Educ. 2024;10:e51148.
50. Khan AA, Yunus R, Sohail M, et al. Artificial Intelligence for Anesthesiology Board-Style Examination Questions: Role of Large Language Models. J Cardiothorac Vasc Anesth. 2024;38(5):1251-1259.
51. Sheikh MS, Thongprayoon C, Qureshi F, et al. Personalized Medicine Transformed: ChatGPT's Contribution to Continuous Renal Replacement Therapy Alarm Management in Intensive Care Units. J Pers Med. 2024;14(3):233.
52. Mayo-Yáñez M, Lechien JR, Maria-Saibene A, Vaira LA, Maniaci A, Chiesa-Estomba CM. Examining the Performance of ChatGPT 3.5 and Microsoft Copilot in Otolaryngology: A Comparative Study with Otolaryngologists' Evaluation. Indian J Otolaryngol Head Neck Surg. 2024;76(4):3465-3469.
53. Rydzewski NR, Dinakaran D, Zhao SG, et al. Comparative Evaluation of LLMs in Clinical Oncology. NEJM AI. 2024;1(5):10.1056/aioa2300151.
54. Wang T, Mu J, Chen J, Lin CC. Comparing ChatGPT and clinical nurses' performances on tracheostomy care: A cross-sectional study. Int J Nurs Stud Adv. 2024;6:100181.
55. Liang R, Zhao A, Peng L, et al. Enhanced Artificial Intelligence Strategies in Renal Oncology: Iterative Optimization and Comparative Analysis of GPT 3.5 Versus 4.0. Ann Surg Oncol. 2024;31(6):3887-3893.
56. Jaworski A, Jasiński D, Jaworski W, et al. Comparison of the Performance of Artificial Intelligence Versus Medical Professionals in the Polish Final Medical Examination. Cureus. 2024;16(8):e66011.
57. Bharatha A, Ojeh N, Fazle Rabbi AM, et al. Comparing the Performance of ChatGPT-4 and Medical Students on MCQs at Varied Levels of Bloom's Taxonomy. Adv Med Educ Pract. 2024;15:393-400.
58. Le M, Davis M. ChatGPT Yields a Passing Score on a Pediatric Board Preparatory Exam but Raises Red Flags. Glob Pediatr Health. 2024;11:2333794X241240327. Published 2024 Mar 24.
59. Arango SD, Flynn JC, Zeitlin J, et al. The Performance of ChatGPT on the American Society for Surgery of the Hand Self-Assessment Examination. Cureus. 2024;16(4):e58950.
60. Rojas M, Rojas M, Burgess V, Toro-Pérez J, Salehi S. Exploring the Performance of ChatGPT Versions 3.5, 4, and 4 With Vision in the Chilean Medical Licensing Examination: Observational Study. JMIR Med Educ. 2024;10:e55048.
61. Chau RCW, Thu KM, Yu OY, Hsung RT, Lo ECM, Lam WYH. Performance of Generative Artificial Intelligence in Dental Licensing Examinations. Int Dent J. 2024;74(3):616-621.
62. Thirunavukarasu AJ, Mahmood S, Malem A, et al. Large language models approach expert-level clinical knowledge and reasoning in ophthalmology: A head-to-head cross-sectional study. PLOS Digit Health. 2024;3(4):e0000341.
63. Bicknell BT, Butler D, Whalen S, et al. Critical Analysis of ChatGPT 4 Omni in USMLE Disciplines, Clinical Clerkships, and Clinical Skills. JMIR Med Educ. Published online September 14, 2024.
64. Haddad F, Saade JS. Performance of ChatGPT on Ophthalmology-Related Questions Across Various Examination Levels: Observational Study. JMIR Med Educ. 2024;10:e50842.
65. Noda R, Izaki Y, Kitano F, Komatsu J, Ichikawa D, Shibagaki Y. Performance of ChatGPT and Bard in self-assessment questions for nephrology board renewal. Clin Exp Nephrol. 2024;28(5):465-469.
66. Yudovich MS, Makarova E, Hague CM, Raman JD. Performance of GPT-3.5 and GPT-4 on standardized urology knowledge assessment items in the United States: a descriptive study. J Educ Eval Health Prof. 2024;21:17.
67. Li DJ, Kao YC, Tsai SJ, et al. Comparing the performance of ChatGPT GPT-4, Bard, and Llama-2 in the Taiwan Psychiatric Licensing Examination and in differential diagnosis with multi-center psychiatrists. Psychiatry Clin Neurosci. 2024;78(6):347-352.
68. Farhat F, Chaudhry BM, Nadeem M, Sohail SS, Madsen DØ. Evaluating Large Language Models for the National Premedical Exam in India: Comparative Analysis of GPT-3.5, GPT-4, and Bard. JMIR Med Educ. 2024;10:e51523.
69. Gilson A, Safranek CW, Huang T, et al. How Does ChatGPT Perform on the United States Medical Licensing Examination (USMLE)? The Implications of Large Language Models for Medical Education and Knowledge Assessment [published correction appears in JMIR Med Educ. 2024 Feb 27;10:e57594.
70. Kung JE, Marshall C, Gauthier C, Gonzalez TA, Jackson JB 3rd. Evaluating ChatGPT Performance on the Orthopaedic In-Training Examination. JB JS Open Access. 2023;8(3):e23.00056.
71. Gencer A, Aydin S. Can ChatGPT pass the thoracic surgery exam?. Am J Med Sci. 2023;366(4):291-295.
72. Ali R, Tang OY, Connolly ID, et al. Performance of ChatGPT and GPT-4 on Neurosurgery Written Board Examinations. Neurosurgery. 2023;93(6):1353-1365。
73. Massey PA, Montgomery C, Zhang AS. Comparison of ChatGPT-3.5, ChatGPT-4, and Orthopaedic Resident Performance on Orthopaedic Assessment Examinations. J Am Acad Orthop Surg. 2023;31(23):1173-1179.
74. Suchman K, Garg S, Trindade AJ. Chat Generative Pretrained Transformer Fails the Multiple-Choice American College of Gastroenterology Self-Assessment Test. Am J Gastroenterol. 2023;118(12):2280-2282.
75. Sakai D, Maeda T, Ozaki A, Kanda GN, Kurimoto Y, Takahashi M. Performance of ChatGPT in Board Examinations for Specialists in the Japanese Ophthalmology Society. Cureus. 2023;15(12):e49903.
76. Huang Y, Gomaa A, Semrau S, et al. Benchmarking ChatGPT-4 on a radiation oncology in-training exam and Red Journal Gray Zone cases: potentials and challenges for ai-assisted medical education and decision making in radiation oncology. Front Oncol. 2023;13:1265024.
77. Yanagita Y, Yokokawa D, Uchida S, Tawara J, Ikusaka M. Accuracy of ChatGPT on Medical Questions in the National Medical Licensing Examination in Japan: Evaluation Study. JMIR Form Res. 2023;7:e48023.
78. Teebagy S, Colwell L, Wood E, Yaghy A, Faustina M. Improved Performance of ChatGPT-4 on the OKAP Examination: A Comparative Study with ChatGPT-3.5. J Acad Ophthalmol (2017). 2023;15(2):e184-e187.
79. Kaneda Y, Takahashi R, Kaneda U, et al. Assessing the Performance of GPT-3.5 and GPT-4 on the 2023 Japanese Nursing Examination. Cureus. 2023;15(8):e42924.
80. Flores-Cohaila JA, García-Vicente A, Vizcarra-Jiménez SF, et al. Performance of ChatGPT on the Peruvian National Licensing Medical Examination: Cross-Sectional Study. JMIR Med Educ. 2023;9:e48039.
81. Fowler T, Pullen S, Birkett L. Performance of ChatGPT and Bard on the official part 1 FRCOphth practice questions. Br J Ophthalmol. 2024;108(10):1379-1383.
82. Moshirfar M, Altaf AW, Stoakes IM, Tuttle JJ, Hoopes PC. Artificial Intelligence in Ophthalmology: A Comparative Analysis of GPT-3.5, GPT-4, and Human Expertise in Answering StatPearls Questions. Cureus. 2023;15(6):e40822.
83. Brin D, Sorin V, Vaid A, et al. Comparing ChatGPT and GPT-4 performance in USMLE soft skill assessments. Sci Rep. 2023;13(1):16492.
84. Miao J, Thongprayoon C, Garcia Valencia OA, et al. Performance of ChatGPT on Nephrology Test Questions. Clin J Am Soc Nephrol. 2024;19(1):35-43.
85. Kaneda Y, Namba M, Kaneda U, Tanimoto T. Artificial Intelligence in Childcare: Assessing the Performance and Acceptance of ChatGPT Responses. Cureus. 2023;15(8):e44484.
86. Takagi S, Watari T, Erabi A, Sakaguchi K. Performance of GPT-3.5 and GPT-4 on the Japanese Medical Licensing Examination: Comparison Study. JMIR Med Educ. 2023;9:e48002.
87. Ali R, Tang OY, Connolly ID, et al. Performance of ChatGPT, GPT-4, and Google Bard on a Neurosurgery Oral Boards Preparation Question Bank. Neurosurgery. 2023;93(5):1090-1098.
88. Ohta K, Ohta S. The Performance of GPT-3.5, GPT-4, and Bard on the Japanese National Dentist Examination: A Comparison Study. Cureus. 2023;15(12):e50369.
89. Watari T, Takagi S, Sakaguchi K, et al. Performance Comparison of ChatGPT-4 and Japanese Medical Residents in the General Medicine In-Training Examination: Comparison Study. JMIR Med Educ. 2023;9:e52202.
90. Roos J, Kasapovic A, Jansen T, Kaczmarczyk R. Artificial Intelligence in Medical Education: Comparative Analysis of ChatGPT, Bing, and Medical Students in Germany. JMIR Med Educ. 2023;9:e46482.
91. Guillen-Grima F, Guillen-Aguinaga S, Guillen-Aguinaga L, et al. Evaluating the Efficacy of ChatGPT in Navigating the Spanish Medical Residency Entrance Examination (MIR): Promising Horizons for AI in Clinical Medicine. Clin Pract. 2023;13(6):1460-1487.
92. Huang RS, Lu KJQ, Meaney C, Kemppainen J, Punnett A, Leung FH. Assessment of Resident and AI Chatbot Performance on the University of Toronto Family Medicine Residency Progress Test: Comparative Study. JMIR Med Educ. 2023;9:e50514.
93. Schubert MC, Wick W, Venkataramani V. Performance of Large Language Models on a Neurology Board-Style Examination [published correction appears in JAMA Netw Open. 2024 Jan 2;7(1):e240194.
94. Torres-Zegarra BC, Rios-Garcia W, Ñaña-Cordova AM, et al. Performance of ChatGPT, Bard, Claude, and Bing on the Peruvian National Licensing Medical Examination: a cross-sectional study. J Educ Eval Health Prof. 2023;20:30.
95. Kirshteyn G, Golan R, Chaet M. Performance of ChatGPT vs. HuggingChat on OB-GYN Topics. Cureus. 2024;16(3):e56187.
96. van Nuland M, Erdogan A, Aςar C, et al. Performance of ChatGPT on Factual Knowledge Questions Regarding Clinical Pharmacy. J Clin Pharmacol. 2024;64(9):1095-1100.
97. Danesh A, Pazouki H, Danesh F, Danesh A, Vardar-Sengul S. Artificial intelligence in dental education: ChatGPT's performance on the periodontic in-service examination. J Periodontol. 2024;95(7):682-687.
98. Huang CY, Zhang E, Caussade MC, Brown T, Stockton Hogrogian G, Yan AC. Pediatric dermatologists versus AI bots: Evaluating the medical knowledge and diagnostic capabilities of ChatGPT. Pediatr Dermatol. 2024;41(5):831-834.
99. Fiedler B, Azua EN, Phillips T, Ahmed AS. ChatGPT performance on the American Shoulder and Elbow Surgeons maintenance of certification exam. J Shoulder Elbow Surg. 2024;33(9):1888-1893.
100. Coleman MC, Moore JN. Two artificial intelligence models underperform on examinations in a veterinary curriculum. J Am Vet Med Assoc. 2024;262(5):692-697.
101. Abbas A, Rehman MS, Rehman SS. Comparing the Performance of Popular Large Language Models on the National Board of Medical Examiners Sample Questions. Cureus. 2024;16(3):e55991.
102. Jarou ZJ, Dakka A, McGuire D, Bunting L. ChatGPT Versus Human Performance on Emergency Medicine Board Preparation Questions. Ann Emerg Med. 2024;83(1):87-88.
103. Sensoy E, Citirik M. Assessing the proficiency of artificial intelligence programs in the diagnosis and treatment of cornea, conjunctiva, and eyelid diseases and exploring the advantages of each other benefits. Cont Lens Anterior Eye. 2024;47(2):102125.
104. Guerra GA, Hofmann HL, Le JL, et al. ChatGPT, Bard, and Bing Chat are large language processing models that answered OITE questions with a similar accuracy to first-year orthopaedic surgery residents. Arthroscopy. Published online August 27, 2024.
105. Agarwal M, Goswami A, Sharma P. Evaluating ChatGPT-3.5 and Claude-2 in Answering and Explaining Conceptual Medical Physiology Multiple-Choice Questions. Cureus. 2023;15(9):e46222.
106. Cheong KX, Zhang C, Tan TE, et al. Comparing generative and retrieval-based chatbots in answering patient questions regarding age-related macular degeneration and diabetic retinopathy. Br J Ophthalmol. 2024;108(10):1443-1449.
107. Zhou S, Luo X, Chen C, et al. The performance of large language model powered chatbots compared to oncology physicians on colorectal cancer queries. Int J Surg. Published online June 27, 2024.
108. Kozaily E, Geagea M, Akdogan ER, et al. Accuracy and consistency of online large language model-based artificial intelligence chat platforms in answering patients' questions about heart failure. Int J Cardiol. 2024;408:132115.
109. Xia S, Hua Q, Mei Z, et al. Clinical application potential of large language model: a study based on thyroid nodules. Endocrine. Published online July 30, 2024.
110. Lee Y, Shin T, Tessier L, et al. Harnessing artificial intelligence in bariatric surgery: comparative analysis of ChatGPT-4, Bing, and Bard in generating clinician-level bariatric surgery recommendations. Surg Obes Relat Dis. 2024;20(7):603-608.
111. Doğan L, Özçakmakcı GB, Yılmaz ĬE. The Performance of Chatbots and the AAPOS Website as a Tool for Amblyopia Education. J Pediatr Ophthalmol Strabismus. 2024;61(5):325-331.
112. Lee TJ, Campbell DJ, Patel S, et al. Unlocking Health Literacy: The Ultimate Guide to Hypertension Education From ChatGPT Versus Google Gemini. Cureus. 2024;16(5):e59898.
113. Lang SP, Yoseph ET, Gonzalez-Suarez AD, et al. Analyzing Large Language Models' Responses to Common Lumbar Spine Fusion Surgery Questions: A Comparison Between ChatGPT and Bard. Neurospine. 2024;21(2):633-641.
114. Iannantuono GM, Bracken-Clarke D, Karzai F, Choo-Wosoba H, Gulley JL, Floudas CS. Comparison of Large Language Models in Answering Immuno-Oncology Questions: A Cross-Sectional Study. Oncologist. 2024;29(5):407-414.
115. Anguita R, Downie C, Ferro Desideri L, Sagoo MS. Assessing large language models' accuracy in providing patient support for choroidal melanoma. Eye (Lond). Published online July 13, 2024.
116. Zhang Y, Dong Y, Mei Z, et al. Performance of large language models on benign prostatic hyperplasia frequently asked questions. Prostate. 2024;84(9):807-813.
117. Xue E, Bracken-Clarke D, Iannantuono GM, Choo-Wosoba H, Gulley JL, Floudas CS. Utility of Large Language Models for Health Care Professionals and Patients in Navigating Hematopoietic Stem Cell Transplantation: Comparison of the Performance of ChatGPT-3.5, ChatGPT-4, and Bard. J Med Internet Res. 2024;26:e54758.
118. Cao JJ, Kwon DH, Ghaziani TT, et al. Large language models' responses to liver cancer surveillance, diagnosis, and management questions: accuracy, reliability, readability. Abdom Radiol (NY).
119. Monroe CL, Abdelhafez YG, Atsina K, Aman E, Nardo L, Madani MH. Evaluation of responses to cardiac imaging questions by the artificial intelligence large language model ChatGPT. Clin Imaging. 2024;112:110193.
120. Chervonski E, Harish KB, Rockman CB, et al. Generative artificial intelligence chatbots may provide appropriate informational responses to common vascular surgery questions by patients. Vascular. Published online March 18, 2024.
121. Kassab J, Hadi El Hajjar A, Wardrop RM 3rd, Brateanu A. Accuracy of Online Artificial Intelligence Models in Primary Care Settings. Am J Prev Med. 2024;66(6):1054-1059.
122. Al-Sharif EM, Penteado RC, Dib El Jalbout N, et al. Evaluating the Accuracy of ChatGPT and Google BARD in Fielding Oculoplastic Patient Queries: A Comparative Study on Artificial versus Human Intelligence. Ophthalmic Plast Reconstr Surg. 2024;40(3):303-311.
123. Mejia MR, Arroyave JS, Saturno M, et al. Use of ChatGPT for Determining Clinical and Surgical Treatment of Lumbar Disc Herniation With Radiculopathy: A North American Spine Society Guideline Comparison. Neurospine. 2024;21(1):149-158.
124. Lee TJ, Rao AK, Campbell DJ, Radfar N, Dayal M, Khrais A. Evaluating ChatGPT-3.5 and ChatGPT-4.0 Responses on Hyperlipidemia for Patient Education. Cureus. 2024;16(5):e61067.
125. Oliveira AL, Coelho M, Guedes LC, Cattoni MB, Carvalho H, Duarte-Batista P. Performance of ChatGPT 3.5 and 4 as a tool for patient support before and after DBS surgery for Parkinson's disease. Neurol Sci. Published online August 29, 2024.
126. Lim ZW, Pushpanathan K, Yew SME, et al. Benchmarking large language models' performances for myopia care: a comparative analysis of ChatGPT-3.5, ChatGPT-4.0, and Google Bard. EBioMedicine. 2023;95:104770.
127. Rahsepar AA, Tavakoli N, Kim GHJ, Hassani C, Abtin F, Bedayat A. How AI Responds to Common Lung Cancer Questions: ChatGPT vs Google Bard. Radiology. 2023;307(5):e230922.
128. Pushpanathan K, Lim ZW, Er Yew SM, et al. Popular large language model chatbots' accuracy, comprehensiveness, and self-awareness in answering ocular symptom queries. iScience. 2023;26(11):108163. Published 2023 Oct 10.
129. Coskun BN, Yagiz B, Ocakoglu G, Dalkilic E, Pehlivan Y. Assessing the accuracy and completeness of artificial intelligence language models in providing information on methotrexate use. Rheumatol Int. 2024;44(3):509-515.
130. King RC, Samaan JS, Yeo YH, et al. A Multidisciplinary Assessment of ChatGPT's Knowledge of Amyloidosis: Observational Study. JMIR Cardio. 2024;8:e53421.
131. Pinto VBP, de Azevedo MF, Wroclawski ML, et al. Conformity of ChatGPT recommendations with the AUA/SUFU guideline on postprostatectomy urinary incontinence. Neurourol Urodyn. 2024;43(4):935-941.
132. Momenaei B, Wakabayashi T, Shahlaee A, et al. Assessing ChatGPT-3.5 Versus ChatGPT-4 Performance in Surgical Treatment of Retinal Diseases: A Comparative Study. Ophthalmic Surg Lasers Imaging Retina. 2024;55(8):481-482.
133. Stevenson E, Walsh C, Hibberd L. Can artificial intelligence replace biochemists? A study comparing interpretation of thyroid function test results by ChatGPT and Google Bard to practising biochemists. Ann Clin Biochem. 2024;61(2):143-149.
134. Dronkers EAC, Geneid A, Al Yaghchi C, Lechien JR. Evaluating the Potential of AI Chatbots in Treatment Decision-making for Acquired Bilateral Vocal Fold Paralysis in Adults. J Voice. Published online April 6, 2024.
135. Rahimli Ocakoglu S, Coskun B. The Emerging Role of AI in Patient Education: A Comparative Analysis of LLM Accuracy for Pelvic Organ Prolapse. Med Princ Pract. Published online March 25, 2024.
136. Gandhi AP, Joesph FK, Rajagopal V, et al. Performance of ChatGPT on the India Undergraduate Community Medicine Examination: Cross-Sectional Study. JMIR Form Res. 2024;8:e49964.
137. Tariq R, Malik S, Khanna S. Evolving Landscape of Large Language Models: An Evaluation of ChatGPT and Bard in Answering Patient Queries on Colonoscopy. Gastroenterology. 2024;166(1):220-221.
138. Li P, Zhang X, Zhu E, et al. Potential Multidisciplinary Use of Large Language Models for Addressing Queries in Cardio-Oncology. J Am Heart Assoc. 2024;13(6):e033584.
139. Sosa BR, Cung M, Suhardi VJ, et al. Capacity for large language model chatbots to aid in orthopedic management, research, and patient queries. J Orthop Res. 2024;42(6):1276-1282.
140. Shukla R, Mishra AK, Banerjee N, Verma A. The Comparison of ChatGPT 3.5, Microsoft Bing, and Google Gemini for Diagnosing Cases of Neuro-Ophthalmology. Cureus. 2024;16(4):e58232.
141. Koga S, Martin NB, Dickson DW. Evaluating the performance of large language models: ChatGPT and Google Bard in generating differential diagnoses in clinicopathological conferences of neurodegenerative disorders. Brain Pathol. 2024;34(3):e13207.
142. Warrier A, Singh R, Haleem A, Zaki H, Eloy JA. The Comparative Diagnostic Capability of Large Language Models in Otolaryngology. Laryngoscope. 2024;134(9):3997-4002.
143. Kumar RP, Sivan V, Bachir H, et al. Can Artificial Intelligence Mitigate Missed Diagnoses by Generating Differential Diagnoses for Neurosurgeons?. World Neurosurg. 2024;187:e1083-e1088.
144. Hirosawa T, Harada Y, Mizuta K, Sakamoto T, Tokumasu K, Shimizu T. Diagnostic performance of generative artificial intelligences for a series of complex case reports. Digit Health. 2024;10:20552076241265215.
145. Mandalos A, Tsouris D. Artificial Versus Human Intelligence in the Diagnostic Approach of Ophthalmic Case Scenarios: A Qualitative Evaluation of Performance and Consistency. Cureus. 2024;16(6):e62471.
146. Krusche M, Callhoff J, Knitza J, Ruffer N. Diagnostic accuracy of a large language model in rheumatology: comparison of physician and ChatGPT-4. Rheumatol Int. 2024;44(2):303-306.
147. Delsoz M, Madadi Y, Raja H, et al. Performance of ChatGPT in Diagnosis of Corneal Eye Diseases. Cornea. 2024;43(5):664-670.
148. Kozel G, Gurses ME, Gecici NN, et al. Chat-GPT on brain tumors: An examination of Artificial Intelligence/Machine Learning's ability to provide diagnoses and treatment plans for example neuro-oncology cases. Clin Neurol Neurosurg. 2024;239:108238.
149. Stoneham S, Livesey A, Cooper H, Mitchell C. ChatGPT versus clinician: challenging the diagnostic capabilities of artificial intelligence in dermatology. Clin Exp Dermatol. 2024;49(7):707-710.
150. Albaladejo A, Lorleac'h A, Allain JS. Les Printemps de la Médecine Interne : l’intelligence artificielle face aux experts internistes [The spring of artificial intelligence: AI vs. expert for internal medicine cases]. Rev Med Interne. 2024;45(7):409-414.
151. Zandi R, Fahey JD, Drakopoulos M, et al. Exploring Diagnostic Precision and Triage Proficiency: A Comparative Study of GPT-4 and Bard in Addressing Common Ophthalmic Complaints. Bioengineering (Basel). 2024;11(2):120. Published 2024 Jan 26
152. Hirosawa T, Kawamura R, Harada Y, et al. ChatGPT-Generated Differential Diagnosis Lists for Complex Case-Derived Clinical Vignettes: Diagnostic Accuracy Evaluation. JMIR Med Inform. 2023;11:e48808.
153. Hirosawa T, Harada Y, Yokose M, Sakamoto T, Kawamura R, Shimizu T. Diagnostic Accuracy of Differential-Diagnosis Lists Generated by Generative Pretrained Transformer 3 Chatbot for Clinical Vignettes with Common Chief Complaints: A Pilot Study. Int J Environ Res Public Health. 2023;20(4):3378.
154. Fraser H, Crossland D, Bacher I, Ranney M, Madsen T, Hilliard R. Comparison of Diagnostic and Triage Accuracy of Ada Health and WebMD Symptom Checkers, ChatGPT, and Physicians for Patients in an Emergency Department: Clinical Data Analysis Study. JMIR Mhealth Uhealth. 2023;11:e49995.
155. Rojas-Carabali W, Cifuentes-González C, Wei X, et al. Evaluating the Diagnostic Accuracy and Management Recommendations of ChatGPT in Uveitis. Ocul Immunol Inflamm. 2024;32(8):1526-1531.
156. Gräf M, Knitza J, Leipe J, et al. Comparison of physician and artificial intelligence-based symptom checker diagnostic accuracy. Rheumatol Int. 2022;42(12):2167-2176.
157. Ward M, Unadkat P, Toscano D, et al. A Quantitative Assessment of ChatGPT as a Neurosurgical Triaging Tool. Neurosurgery. 2024;95(2):487-495.
158. Hirosawa T, Mizuta K, Harada Y, Shimizu T. Comparative Evaluation of Diagnostic Accuracy Between Google Bard and Physicians. Am J Med. 2023;136(11):1119-1123.e18.
159. Lyons RJ, Arepalli SR, Fromal O, Choi JD, Jain N. Artificial intelligence chatbot performance in triage of ophthalmic conditions. Can J Ophthalmol. 2024;59(4):e301-e308.
160. Makhoul M, Melkane AE, Khoury PE, Hadi CE, Matar N. A cross-sectional comparative study: ChatGPT 3.5 versus diverse levels of medical experts in the diagnosis of ENT diseases. Eur Arch Otorhinolaryngol. 2024;281(5):2717-2721.
161. Shemer A, Cohen M, Altarescu A, et al. Diagnostic capabilities of ChatGPT in ophthalmology. Graefes Arch Clin Exp Ophthalmol. 2024;262(7):2345-2352.
162. Gunes YC, Cesur T. The Diagnostic Performance of Large Language Models and General Radiologists in Thoracic Radiology Cases: A Comparative Study. J Thorac Imaging. Published online September 13, 2024.
163. Sarangi PK, Irodi A, Panda S, Nayak DSK, Mondal H. Radiological Differential Diagnoses Based on Cardiovascular and Thoracic Imaging Patterns: Perspectives of Four Large Language Models. Indian J Radiol Imaging. 2023;34(2):269-275.
164. Berg HT, van Bakel B, van de Wouw L, et al. ChatGPT and Generating a Differential Diagnosis Early in an Emergency Department Presentation. Ann Emerg Med. 2024;83(1):83-86.
165. Haider SA, Pressman SM, Borna S, et al. Evaluating Large Language Model (LLM) Performance on Established Breast Classification Systems. Diagnostics (Basel). 2024;14(14):1491.
166. Pressman SM, Borna S, Gomez-Cabello CA, Haider SA, Forte AJ. AI in Hand Surgery: Assessing Large Language Models in the Classification and Management of Hand Injuries. J Clin Med. 2024;13(10):2832.
167. Gan RK, Ogbodo JC, Wee YZ, Gan AZ, González PA. Performance of Google bard and ChatGPT in mass casualty incidents triage. Am J Emerg Med. 2024;75:72-78.
168. Aiumtrakul N, Thongprayoon C, Arayangkool C, et al. Personalized Medicine in Urolithiasis: AI Chatbot-Assisted Dietary Management of Oxalate for Kidney Stone Prevention. J Pers Med. 2024;14(1).
