# Supplementary material for: Accuracy of Large Language Models When Answering Clinical Research Questions: Systematic Review and Network Meta-Analysis
Source: J Med Internet Res. 2025 Apr 30;27:e64486. doi: 10.2196/64486 (PMC12079073; doi:10.2196/64486)
Supplement: Multimedia Appendix 6 [file jmir_v27i1e64486_app6.docx]

Multimedia Appendix 6 Quality assessment of observational study

| Study ID | Quality assessment（score: 0, 1or2） | | | | | | | | Total score | Overall risk of  bias （L: Low risk, H: High risk, U: Unknoen） | |
| --- | --- | --- | --- | --- | --- | --- | --- | --- | --- | --- | --- |
|  | Representativen  ess of the  exposed cohort  （1 分） | Selection of the non-exposed  cohort  （1 分） | Ascertainme-nt of exposure （1 分） | Demonstration  that outcome of  interest was not  present at start of study  （1 分） | Compar--ability  （2 分） | Assessment of outcome （1 分） | Was follow -up long enough for outcomes to  occur （1 分） | Adequacy of follow- up of cohorts （1 分） |  |  |  |
| Tsoutsanis P，2024 | 1 | 1 | 1 | 1 | 1 | 1 | 0 | 0 | 6 | M | |
| Long C，2024 | 1 | 1 | 1 | 1 | 1 | 1 | 0 | 0 | 6 | M | |
| Tao BK，2024 | 1 | 1 | 1 | 1 | 1 | 1 | 0 | 0 | 6 | M | |
| Shieh A，2024 | 1 | 1 | 1 | 1 | 2 | 1 | 0 | 0 | 7 | L | |
| Sarangi PK，2023 | 1 | 1 | 1 | 1 | 1 | 1 | 0 | 0 | 6 | M | |
| Singer MB，2024 | 1 | 1 | 1 | 1 | 1 | 1 | 0 | 0 | 6 | M | |
| Hanna RE，2024 | 1 | 1 | 1 | 1 | 1 | 1 | 0 | 0 | 6 | M | |
| Kadoya N，2024 | 1 | 1 | 1 | 1 | 1 | 1 | 0 | 0 | 6 | M | |
| Sallam M，2024 | 1 | 1 | 1 | 1 | 1 | 1 | 0 | 0 | 6 | M | |
| Gravina AG，2024 | 1 | 1 | 1 | 1 | 1 | 1 | 0 | 0 | 6 | M | |
| Passby L，2024 | 1 | 1 | 1 | 1 | 1 | 1 | 0 | 0 | 6 | M | |
| Sabri H，2024 | 1 | 1 | 1 | 1 | 1 | 1 | 0 | 0 | 6 | M | |
| Çamur E，2024 | 1 | 1 | 1 | 1 | 1 | 1 | 0 | 0 | 6 | M | |
| Lubitz M，2024 | 1 | 1 | 1 | 1 | 1 | 1 | 0 | 0 | 6 | M | |
| Gupta R，2024 | 1 | 1 | 1 | 1 | 1 | 1 | 0 | 0 | 6 | M | |
| Lee GU，2024 | 1 | 1 | 1 | 1 | 1 | 1 | 0 | 0 | 6 | M | |
| Is EE，2024 | 1 | 1 | 1 | 1 | 1 | 1 | 0 | 0 | 6 | M | |
| D'Anna G，2024 | 1 | 1 | 1 | 1 | 1 | 1 | 0 | 0 | 6 | M | |
| Altamimi I，2024 | 1 | 1 | 1 | 1 | 1 | 1 | 0 | 0 | 6 | M | |
| Lee Y，2024 | 1 | 1 | 1 | 1 | 2 | 1 | 0 | 0 | 7 | L | |
| Schoch J，2024 | 1 | 1 | 1 | 1 | 1 | 1 | 0 | 0 | 6 | M | |
| May M，2024 | 1 | 1 | 1 | 1 | 1 | 1 | 0 | 0 | 6 | M | |
| Sadeq MA，2024 | 1 | 1 | 1 | 1 | 1 | 1 | 0 | 0 | 6 | M | |
| Khalpey Z，2024 | 1 | 1 | 1 | 1 | 1 | 1 | 0 | 0 | 6 | M | |
| Patel EA，2024 | 1 | 1 | 1 | 1 | 1 | 1 | 0 | 0 | 6 | M | |
| Irmici G，2024 | 1 | 1 | 1 | 1 | 2 | 1 | 0 | 0 | 7 | L | |
| Kollitsch L，2024 | 1 | 1 | 1 | 1 | 2 | 1 | 0 | 0 | 7 | L | |
| Morreel S，2024 | 1 | 1 | 1 | 1 | 1 | 1 | 0 | 0 | 6 | M | |
| Bajčetić M，2024 | 1 | 1 | 1 | 1 | 1 | 1 | 0 | 0 | 6 | M | |
| Canillas Del Rey F，2024 | 1 | 1 | 1 | 1 | 1 | 1 | 0 | 0 | 6 | M | |
| Meyer A，2024 | 1 | 1 | 1 | 1 | 1 | 1 | 0 | 0 | 6 | M | |
| Toyama Y，2024 | 1 | 1 | 1 | 1 | 1 | 1 | 0 | 0 | 6 | M | |
| Touma NJ，2024 | 1 | 1 | 1 | 1 | 1 | 1 | 0 | 0 | 6 | M | |
| Chan J，2024 | 1 | 1 | 1 | 1 | 1 | 1 | 0 | 0 | 6 | M | |
| Patil NS，2024 | 1 | 1 | 1 | 1 | 1 | 1 | 0 | 0 | 6 | M | |
| Hubany SS，2024 | 1 | 1 | 1 | 1 | 1 | 1 | 0 | 0 | 6 | M | |
| Vaishya R，2024 | 1 | 1 | 1 | 1 | 1 | 1 | 0 | 0 | 6 | M | |
| Nakajima N，2024 | 1 | 1 | 1 | 1 | 1 | 1 | 0 | 0 | 6 | M | |
| Thibaut G，2024 | 1 | 1 | 1 | 1 | 1 | 1 | 0 | 0 | 6 | M | |
| Lum ZC，2024 | 1 | 1 | 1 | 1 | 1 | 1 | 0 | 0 | 6 | M | |
| Menekşeoğlu AK，2024 | 1 | 1 | 1 | 1 | 1 | 1 | 0 | 0 | 6 | M | |
| Cheong RCT，2024 | 1 | 1 | 1 | 1 | 1 | 1 | 0 | 0 | 6 | M | |
| Mesnard B，2024 | 1 | 1 | 1 | 1 | 1 | 1 | 0 | 0 | 6 | M | |
| Ming S，2024 | 1 | 1 | 1 | 1 | 1 | 1 | 1 | 1 | 8 | L | |
| Chow R，2024 | 1 | 1 | 1 | 1 | 1 | 1 | 0 | 0 | 6 | M | |
| Kim SE，2024 | 1 | 1 | 1 | 1 | 1 | 1 | 0 | 0 | 6 | M | |
| Oura T，2024 | 1 | 1 | 1 | 1 | 1 | 1 | 0 | 0 | 6 | M | |
| Lewandowski M，2024 | 1 | 1 | 1 | 1 | 1 | 1 | 0 | 0 | 6 | M | |
| Knoedler L，2024 | 1 | 1 | 1 | 1 | 1 | 1 | 0 | 0 | 6 | M | |
| Khan AA，2024 | 1 | 1 | 1 | 1 | 1 | 1 | 0 | 0 | 6 | M | |
| Sheikh MS，2024 | 1 | 1 | 1 | 1 | 1 | 1 | 0 | 0 | 6 | M | |
| Mayo-Yáñez M，2024 | 1 | 1 | 1 | 1 | 1 | 1 | 0 | 0 | 6 | M | |
| Rydzewski NR，2024 | 1 | 1 | 1 | 1 | 1 | 1 | 1 | 1 | 8 | L | |
| Wang T，2024 | 1 | 1 | 1 | 1 | 2 | 1 | 0 | 0 | 7 | L | |
| Liang R，2024 | 1 | 1 | 1 | 1 | 1 | 1 | 1 | 1 | 8 | L | |
| Jaworski A，2024 | 1 | 1 | 1 | 1 | 1 | 1 | 0 | 0 | 6 | M | |
| Bharatha A，2024 | 1 | 1 | 1 | 1 | 1 | 1 | 0 | 0 | 6 | M | |
| Le M，2024 | 1 | 1 | 1 | 1 | 1 | 1 | 0 | 0 | 6 | M | |
| Arango SD，2024 | 1 | 1 | 1 | 1 | 1 | 1 | 0 | 0 | 6 | M | |
| Rojas M，2024 | 1 | 1 | 1 | 1 | 1 | 1 | 1 | 1 | 8 | L | |
| Chau RCW，2024 | 1 | 1 | 1 | 1 | 1 | 1 | 0 | 0 | 6 | M | |
| Thirunavukarasu AJ，2024 | 1 | 1 | 1 | 1 | 2 | 1 | 0 | 0 | 7 | L | |
| Bicknell BT，2024 | 1 | 1 | 1 | 1 | 1 | 1 | 0 | 0 | 6 | M | |
| Haddad F，2024 | 1 | 1 | 1 | 1 | 1 | 1 | 0 | 0 | 6 | M | |
| Noda R，2024 | 1 | 1 | 1 | 1 | 1 | 1 | 0 | 0 | 6 | M | |
| Yudovich MS，2024 | 1 | 1 | 1 | 1 | 1 | 1 | 0 | 0 | 6 | M | |
| Li DJ，2024 | 1 | 1 | 1 | 1 | 1 | 1 | 0 | 0 | 6 | M | |
| Farhat F，2024 | 1 | 1 | 1 | 1 | 1 | 1 | 0 | 0 | 6 | M | |
| Gilson A，2023 | 1 | 1 | 1 | 1 | 1 | 1 | 0 | 0 | 6 | M | |
| Kung JE，2023 | 1 | 1 | 1 | 1 | 1 | 1 | 0 | 0 | 6 | M | |
| Gencer A，2023 | 1 | 1 | 1 | 1 | 1 | 1 | 1 | 1 | 8 | L | |
| Ali R，2023 | 1 | 1 | 1 | 1 | 1 | 1 | 0 | 0 | 6 | M | |
| Massey PA，2023 | 1 | 1 | 1 | 1 | 1 | 1 | 0 | 0 | 6 | M | |
| Suchman K，2023 | 1 | 1 | 1 | 1 | 1 | 1 | 0 | 0 | 6 | M | |
| Sakai D，2023 | 1 | 1 | 1 | 1 | 2 | 1 | 0 | 0 | 7 | L | |
| Huang Y，2023 | 1 | 1 | 1 | 1 | 1 | 1 | 0 | 0 | 6 | M | |
| Yanagita Y，2023 | 1 | 1 | 1 | 1 | 1 | 1 | 0 | 0 | 6 | M | |
| Teebagy S，2023 | 1 | 1 | 1 | 1 | 1 | 1 | 0 | 0 | 6 | M | |
| Kaneda Y，2023 | 1 | 1 | 1 | 1 | 1 | 1 | 0 | 0 | 6 | M | |
| Flores-Cohaila JA，2023 | 1 | 1 | 1 | 1 | 1 | 1 | 1 | 1 | 8 | L | |
| Fowler T，2024 | 1 | 1 | 1 | 1 | 1 | 1 | 0 | 0 | 6 | M | |
| Moshirfar M，2023 | 1 | 1 | 1 | 1 | 2 | 1 | 0 | 0 | 7 | L | |
| Brin D，2023 | 1 | 1 | 1 | 1 | 1 | 1 | 0 | 0 | 6 | M | |
| Miao J，2024 | 1 | 1 | 1 | 1 | 1 | 1 | 1 | 1 | 8 | L | |
| Kaneda Y，2023 | 1 | 1 | 1 | 1 | 2 | 1 | 0 | 0 | 7 | L | |
| Takagi S，2023 | 1 | 1 | 1 | 1 | 1 | 1 | 0 | 0 | 6 | M | |
| Ohta K，2023 | 1 | 1 | 1 | 1 | 2 | 1 | 0 | 0 | 7 | L | |
| Watari T，2023 | 1 | 1 | 1 | 1 | 1 | 1 | 0 | 0 | 6 | M | |
| Roos J，2023 | 1 | 1 | 1 | 1 | 1 | 1 | 0 | 0 | 6 | M | |
| Guillen-Grima F，2023 | 1 | 1 | 1 | 1 | 1 | 1 | 0 | 0 | 6 | M | |
| Huang RS，2023 | 1 | 1 | 1 | 1 | 1 | 1 | 0 | 0 | 6 | M | |
| Schubert MC，2024 | 1 | 1 | 1 | 1 | 1 | 1 | 0 | 0 | 6 | M | |
| Torres-Zegarra BC，2023 | 1 | 1 | 1 | 1 | 1 | 1 | 1 | 1 | 8 | L | |
| Kirshteyn G，2024 | 1 | 1 | 1 | 1 | 1 | 1 | 0 | 0 | 6 | M | |
| van Nuland M，2024 | 1 | 1 | 1 | 1 | 1 | 1 | 1 | 1 | 8 | L | |
| Danesh A，2024 | 1 | 1 | 1 | 1 | 1 | 1 | 0 | 0 | 6 | M | |
| Huang CY，2024 | 1 | 1 | 1 | 1 | 1 | 1 | 0 | 0 | 6 | M | |
| Fiedler B，2024 | 1 | 1 | 1 | 1 | 1 | 1 | 0 | 0 | 6 | M | |
| Coleman MC，2024 | 1 | 1 | 1 | 1 | 1 | 1 | 1 | 1 | 8 | L | |
| Abbas A，2024 | 1 | 1 | 1 | 1 | 2 | 1 | 0 | 0 | 7 | L | |
| \| Jarou ZJ，2024 \| \| --- \| | 1 | 1 | 1 | 1 | 1 | 1 | 1 | 1 | 8 | L | |
| Sensoy E，2024 | 1 | 1 | 1 | 1 | 1 | 1 | 0 | 0 | 6 | M | |
| Guerra GA，2024 | 1 | 1 | 1 | 1 | 1 | 1 | 0 | 0 | 6 | M | |
| Agarwal M，2023 | 1 | 1 | 1 | 1 | 1 | 1 | 0 | 0 | 6 | M | |
| Cheong KX，2024 | 1 | 1 | 1 | 1 | 1 | 1 | 0 | 0 | 6 | M | |
| Zhou S，2024 | 1 | 1 | 1 | 1 | 1 | 1 | 1 | 1 | 8 | L | |
| Kozaily E，2024 | 1 | 1 | 1 | 1 | 1 | 1 | 0 | 0 | 6 | M | |
| Xia S，2024 | 1 | 1 | 1 | 1 | 1 | 1 | 1 | 1 | 8 | L | |
| Lee Y，2024 | 1 | 1 | 1 | 1 | 1 | 1 | 0 | 0 | 6 | M | |
| Doğan L，2024 | 1 | 1 | 1 | 1 | 1 | 1 | 1 | 1 | 8 | L | |
| Lee TJ，2024 | 1 | 1 | 1 | 1 | 1 | 1 | 0 | 0 | 6 | M | |
| Lang SP，2024 | 1 | 1 | 1 | 1 | 1 | 1 | 0 | 0 | 6 | M | |
| Iannantuono GM，2024 | 1 | 1 | 1 | 1 | 1 | 1 | 1 | 1 | 8 | L | |
| Anguita R，2024 | 1 | 1 | 1 | 1 | 1 | 1 | 1 | 1 | 8 | L | |
| Zhang Y，2024 | 1 | 1 | 1 | 1 | 1 | 1 | 1 | 1 | 8 | L | |
| Xue E，2024 | 1 | 1 | 1 | 1 | 1 | 1 | 1 | 1 | 8 | L | |
| Cao JJ，2024 | 1 | 1 | 1 | 1 | 1 | 1 | 1 | 1 | 8 | L | |
| Monroe CL，2024 | 1 | 1 | 1 | 1 | 1 | 1 | 1 | 1 | 8 | L | |
| Chervonski E，2024 | 1 | 1 | 1 | 1 | 1 | 1 | 0 | 0 | 6 | M | |
| Kassab J，2024 | 1 | 1 | 1 | 1 | 1 | 1 | 0 | 0 | 6 | M | |
| Al-Sharif EM，2024 | 1 | 1 | 1 | 1 | 1 | 1 | 0 | 0 | 6 | M | |
| Mejia MR，2024 | 1 | 1 | 1 | 1 | 1 | 1 | 0 | 0 | 6 | M | |
| Lee TJ，2024 | 1 | 1 | 1 | 1 | 1 | 1 | 1 | 1 | 8 | L | |
| Oliveira AL，2024 | 1 | 1 | 1 | 1 | 1 | 1 | 0 | 0 | 6 | M | |
| Lim ZW，2023 | 1 | 1 | 1 | 1 | 1 | 1 | 0 | 0 | 6 | M | |
| Rahsepar AA，2023 | 1 | 1 | 1 | 1 | 1 | 1 | 0 | 0 | 6 | M | |
| Pushpanathan K，2023 | 1 | 1 | 1 | 1 | 1 | 1 | 1 | 1 | 8 | L | |
| Coskun BN，2024 | 1 | 1 | 1 | 1 | 1 | 1 | 0 | 0 | 6 | M |  |
| King RC，2024 | 1 | 1 | 1 | 1 | 1 | 1 | 0 | 0 | 6 | M | |
| Pinto VBP，2024 | 1 | 1 | 1 | 1 | 1 | 1 | 0 | 0 | 6 | M | |
| Momenaei B，2024 | 1 | 1 | 1 | 1 | 1 | 1 | 1 | 1 | 8 | L | |
| Stevenson E，2024 | 1 | 1 | 1 | 1 | 1 | 1 | 0 | 0 | 6 | M | |
| Dronkers EAC，2024 | 1 | 1 | 1 | 1 | 1 | 1 | 0 | 0 | 6 | M | |
| Rahimli Ocakoglu S，2024 | 1 | 1 | 1 | 1 | 1 | 1 | 0 | 0 | 6 | M | |
| Gandhi AP，2024 | 1 | 1 | 1 | 1 | 1 | 1 | 0 | 0 | 6 | M | |
| Tariq R，2024 | 1 | 1 | 1 | 1 | 1 | 1 | 1 | 1 | 8 | L | |
| Li P，2024 | 1 | 1 | 1 | 1 | 1 | 1 | 0 | 0 | 6 | M | |
| Sosa BR，2024 | 1 | 1 | 1 | 1 | 1 | 1 | 0 | 0 | 6 | M | |
| Shukla R，2024 | 1 | 1 | 1 | 1 | 1 | 1 | 0 | 0 | 6 | M | |
| Koga S，2024 | 1 | 1 | 1 | 1 | 1 | 1 | 0 | 0 | 6 | M | |
| Warrier A，2024 | 1 | 1 | 1 | 1 | 1 | 1 | 0 | 0 | 6 | M | |
| Kumar RP，2024 | 1 | 1 | 1 | 1 | 1 | 1 | 1 | 1 | 8 | L | |
| Hirosawa T，2024 | 1 | 1 | 1 | 1 | 2 | 1 | 0 | 0 | 7 | L | |
| Mandalos A，2024 | 1 | 1 | 1 | 1 | 1 | 1 | 0 | 0 | 6 | M | |
| Krusche M，2024 | 1 | 1 | 1 | 1 | 1 | 1 | 0 | 0 | 6 | M | |
| Delsoz M，2024 | 1 | 1 | 1 | 1 | 1 | 1 | 0 | 0 | 6 | M | |
| Kozel G，2024 | 1 | 1 | 1 | 1 | 1 | 1 | 0 | 0 | 6 | M | |
| Stoneham S，2024 | 1 | 1 | 1 | 1 | 1 | 1 | 0 | 0 | 6 | M | |
| Albaladejo A，2024 | 1 | 1 | 1 | 1 | 1 | 1 | 0 | 0 | 6 | M | |
| Zandi R，2024 | 1 | 1 | 1 | 1 | 1 | 1 | 0 | 0 | 6 | M | |
| Hirosawa T，2023 | 1 | 1 | 1 | 1 | 2 | 1 | 0 | 0 | 7 | L | |
| Hirosawa T，2023 | 1 | 1 | 1 | 1 | 1 | 1 | 0 | 0 | 6 | M | |
| Fraser H, 2023 | 1 | 1 | 1 | 1 | 1 | 1 | 0 | 0 | 6 | M | |
| Rojas-Carabali W，2024 | 1 | 1 | 1 | 1 | 1 | 1 | 0 | 0 | 6 | M | |
| Gräf M，2022 | 1 | 1 | 1 | 1 | 1 | 1 | 0 | 0 | 6 | M | |
| Ward M，2024 | 1 | 1 | 1 | 1 | 2 | 1 | 0 | 0 | 7 | L | |
| Hirosawa T，2023 | 1 | 1 | 1 | 1 | 1 | 1 | 0 | 0 | 6 | M | |
| Lyons RJ，2024 | 1 | 1 | 1 | 1 | 1 | 1 | 0 | 0 | 6 | M | |
| Makhoul M，2024 | 1 | 1 | 1 | 1 | 1 | 1 | 0 | 0 | 6 | M | |
| Shemer A，2024 | 1 | 1 | 1 | 1 | 1 | 1 | 0 | 0 | 6 | M | |
| Gunes YC，2024 | 1 | 1 | 1 | 1 | 1 | 1 | 0 | 0 | 6 | M | |
| Sarangi PK，2023 | 1 | 1 | 1 | 1 | 1 | 1 | 0 | 0 | 6 | M | |
| Berg HT，2024 | 1 | 1 | 1 | 1 | 1 | 1 | 0 | 0 | 6 | M | |
| Tsoutsanis P，2024 | 1 | 1 | 1 | 1 | 1 | 1 | 0 | 0 | 6 | M | |
| Haider SA，2024 | 1 | 1 | 1 | 1 | 1 | 1 | 0 | 0 | 6 | M | |
| Pressman SM，2024 | 1 | 1 | 1 | 1 | 1 | 1 | 1 | 1 | 8 | L | |
| Gan RK，2024 | 1 | 1 | 1 | 1 | 1 | 1 | 0 | 0 | 6 | M | |
