# Supplementary material for: Accuracy of Large Language Models When Answering Clinical Research Questions: Systematic Review and Network Meta-Analysis
Source: J Med Internet Res. 2025 Apr 30;27:e64486. doi: 10.2196/64486 (PMC12079073; doi:10.2196/64486)
Supplement: Multimedia Appendix 7 [file jmir_v27i1e64486_app7.docx]

**Multimedia Appendix 7** Indirect comparison of accuracy of LLMs in answering objective questions

**
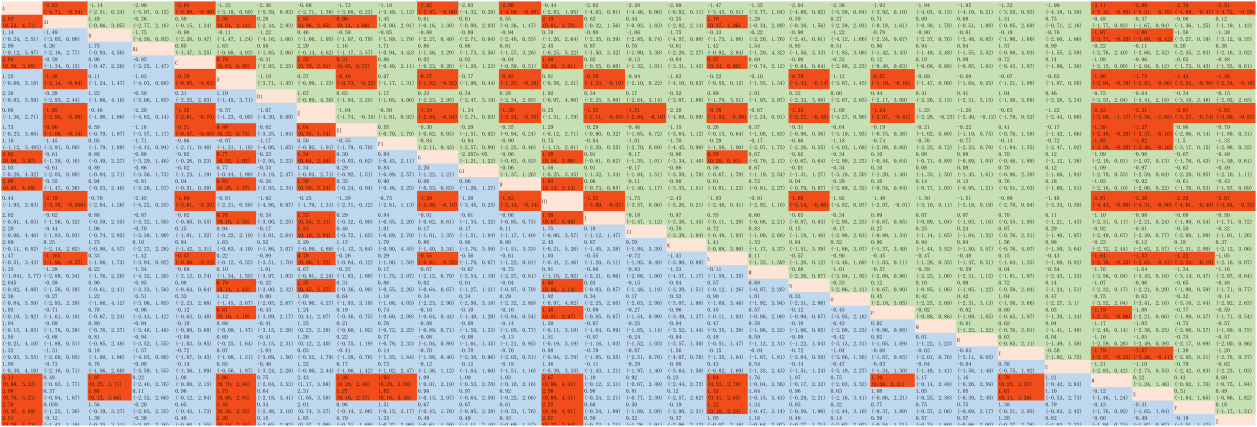
**

Note: A: instructGPT; B: GTP-3; C: ChatGPT-3.5; D: ChatGPT-4; E: ChatGPT-4o; G: Bard; H: PaLM2; I: Gemini; K: Gemini 1.5 pro; L: Bing chat; M: Copilot; N: Perplexity; O: Perplexity Pro; P: Claude; Q: Claude-instant; R: Claude 2; S: Claude 2.1; T: Claude 3 Opus; U: Claude 3 Sonnet; W: LLaMA 7B; X: LLaMA 13B; Y: LLaMA 33B; Z: LLaMA 65B; A1: LLaMA 2; B1: LLaMA 3; C1: Mixtral-8x7B; D1: Mistral Large; E1: people; F1: chatENT; G1: ChatSonic; H1: Aeyeconsult; I1: Med-PaLM 2; J1: OcularBERT; K1: Doctor GPT; L1: Docs-GPT Beta; M1: WebMD; N1: Ada Health

The values in the green and blue cells are the logOR and 95% CI from the comparison of the LLMs represented in the columns with the LLMs represented in the rows. A logOR value less than 0 indicates that the accuracy of the LLM corresponding to a column is lower than the LLM corresponding to a row. A value greater than 0 indicates a higher accuracy. The red cells indicates that the accuracy of both LLMs is statistically significant.

**Multimedia Appendix 7** Indirect comparison of accuracy of LLMs in answering open-ended questions


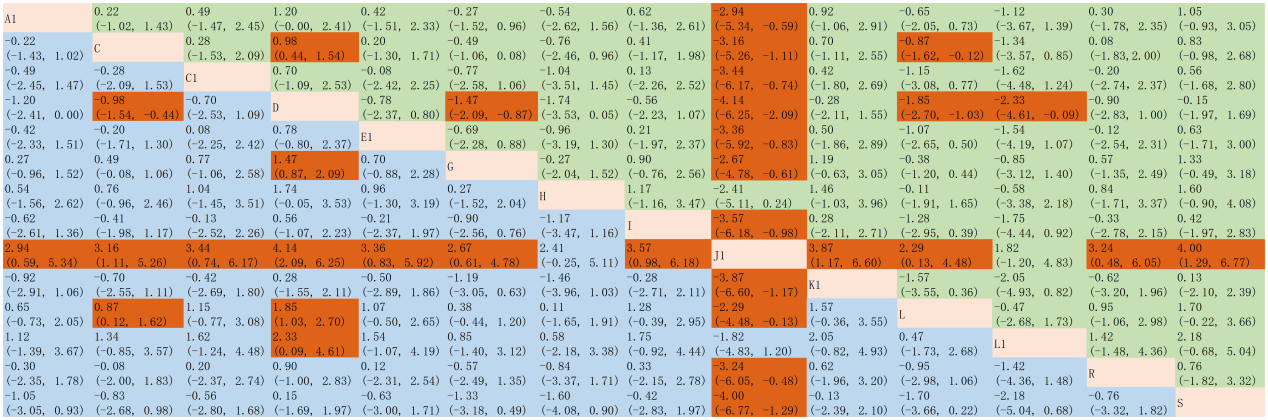


Note: C: ChatGPT-3.5; D: ChatGPT-4; G: Bard; H: PaLM2; I: Gemini; L: Bing chat; R: Claude 2; S: Claude 2.1; A1: LLaMA 2; C1: Mixtral-8x7B; E1: people; J1: OcularBERT; K1: Doctor GPT; L1: Docs-GPT Beta

The values in the green and blue cells are the logOR and 95% CI from the comparison of the LLMs represented in the columns with the LLMs represented in the rows. A logOR value less than 0 indicates that the accuracy of the LLM corresponding to a column is lower than the LLM corresponding to a row. A value greater than 0 indicates a higher accuracy. The red cells indicates that the accuracy of both LLMs is statistically significant.

**Multimedia Appendix 7** Indirect comparison of accuracy of LLMs in top 1 diagnosis


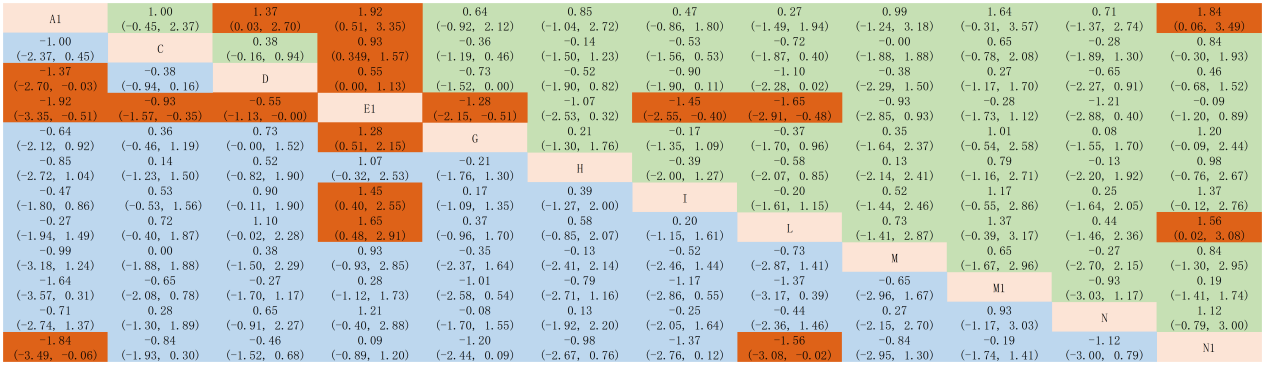


Note: C: ChatGPT-3.5; D: ChatGPT-4; G: Bard; H: PaLM2; I: Gemini; L: Bing chat; M: Copilot; N: Perplexity; A1: LLaMA 2; E1: people; M1: WebMD; N1: Ada Health

The values in the green and blue cells are the logOR and 95% CI from the comparison of the LLMs represented in the columns with the LLMs represented in the rows. A logOR value less than 0 indicates that the accuracy of the LLM corresponding to a column is lower than the LLM corresponding to a row. A value greater than 0 indicates a higher accuracy. The red cells indicates that the accuracy of both LLMs is statistically significant.

**Multimedia Appendix 7** Indirect comparison of accuracy of LLMs in top 3 diagnosis


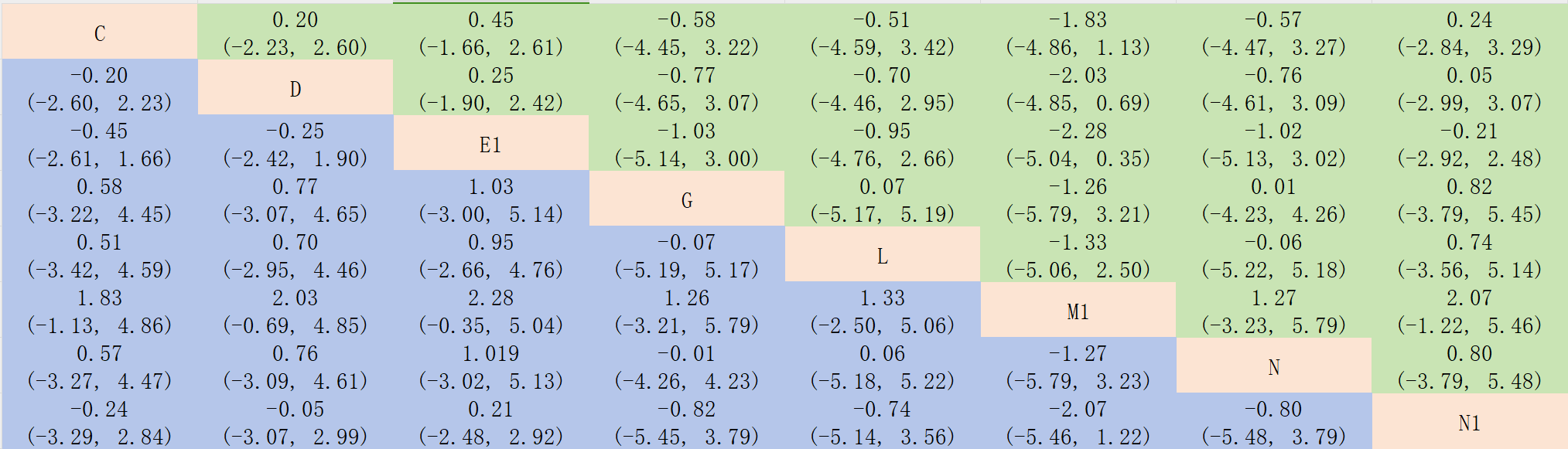


Note: C: ChatGPT-3.5; D: ChatGPT-4; G: Bard; L: Bing chat; N: Perplexity; E1: people; M1: WebMD; N1: Ada Health

The values in the green and blue cells are the logOR and 95% CI from the comparison of the LLMs represented in the columns with the LLMs represented in the rows. A logOR value less than 0 indicates that the accuracy of the LLM corresponding to a column is lower than the LLM corresponding to a row. A value greater than 0 indicates a higher accuracy. The red cells indicates that the accuracy of both LLMs is statistically significant.

**Multimedia Appendix 7** Indirect comparison of accuracy of LLMs in top 5 diagnosis


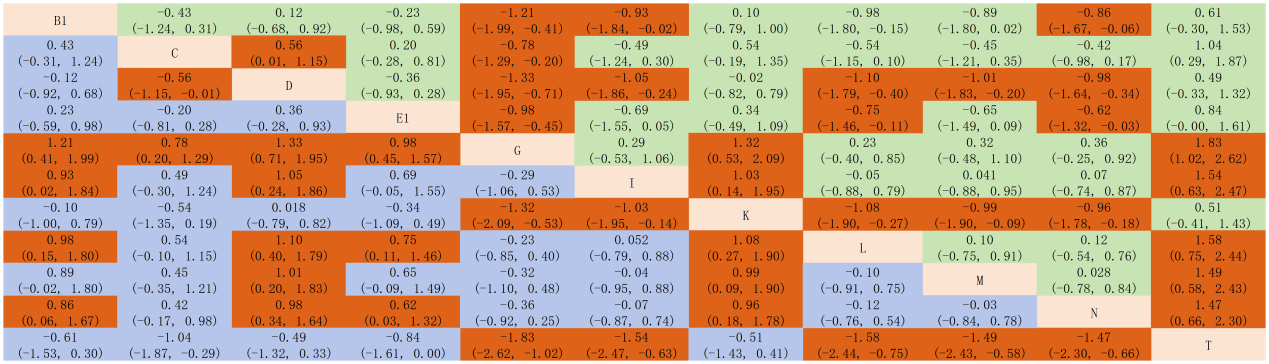


Note: C: ChatGPT-3.5; D: ChatGPT-4; G: Bard; I: Gemini; K: Gemini 1.5 pro; L: Bing chat; M: Copilot; N: Perplexity; T: Claude 3 Opus; B1: LLaMA 3; E1: people

The values in the green and blue cells are the logOR and 95% CI from the comparison of the LLMs represented in the columns with the LLMs represented in the rows. A logOR value less than 0 indicates that the accuracy of the LLM corresponding to a column is lower than the LLM corresponding to a row. A value greater than 0 indicates a higher accuracy. The red cells indicates that the accuracy of both LLMs is statistically significant.

**Multimedia Appendix 7** Indirect comparison of accuracy of LLMs in triage and classification


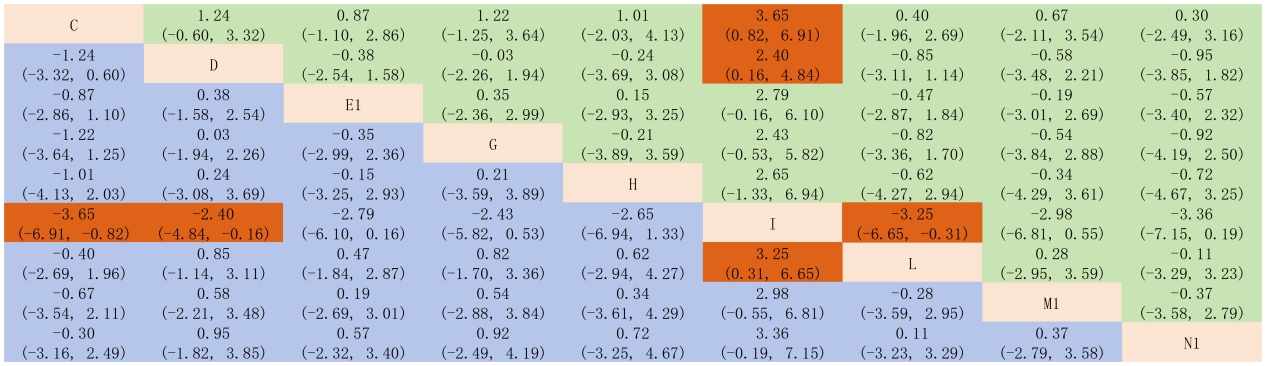


Note: C: ChatGPT-3.5; D: ChatGPT-4; G: Bard; H: PaLM2; I: Gemini; L: Bing chat; E1: people; M1: WebMD; N1: Ada Health

The values in the green and blue cells are the logOR and 95% CI from the comparison of the LLMs represented in the columns with the LLMs represented in the rows. A logOR value less than 0 indicates that the accuracy of the LLM corresponding to a column is lower than the LLM corresponding to a row. A value greater than 0 indicates a higher accuracy. The red cells indicates that the accuracy of both LLMs is statistically significant.
