# Supplementary material for: Accuracy of Large Language Models When Answering Clinical Research Questions: Systematic Review and Network Meta-Analysis
Source: J Med Internet Res. 2025 Apr 30;27:e64486. doi: 10.2196/64486 (PMC12079073; doi:10.2196/64486)
Supplement: Multimedia Appendix 8 [file jmir_v27i1e64486_app8.docx]

**Multimedia Appendix 8** Node splitting inconsistency test for objective questions


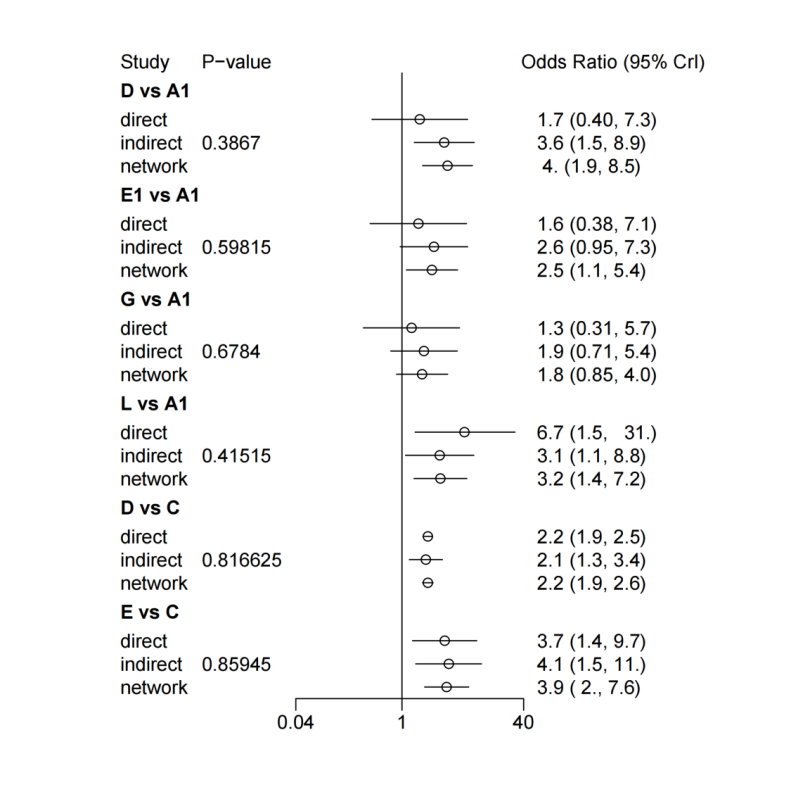

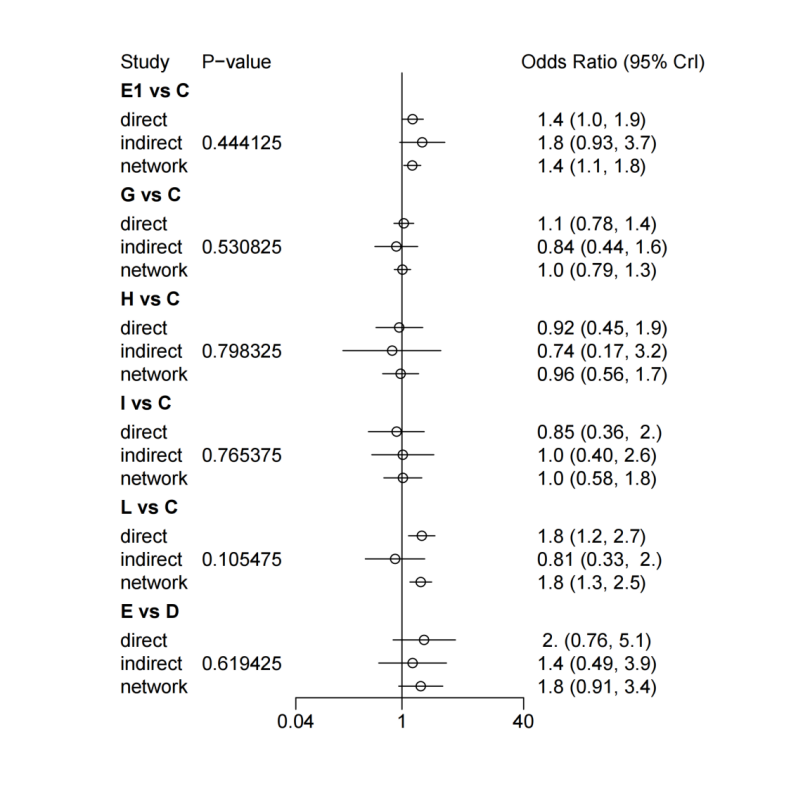


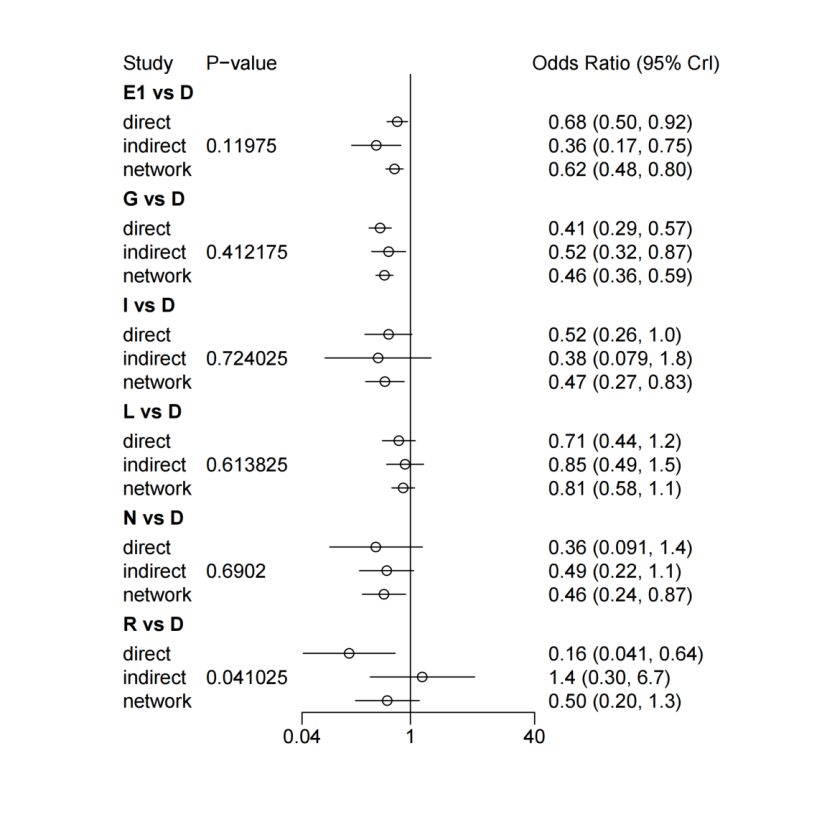

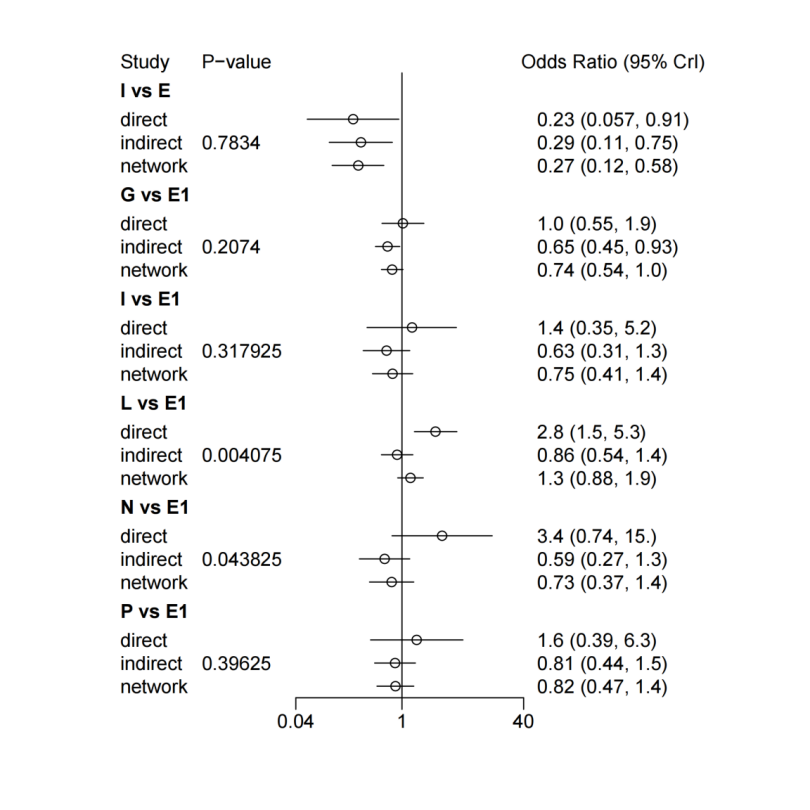

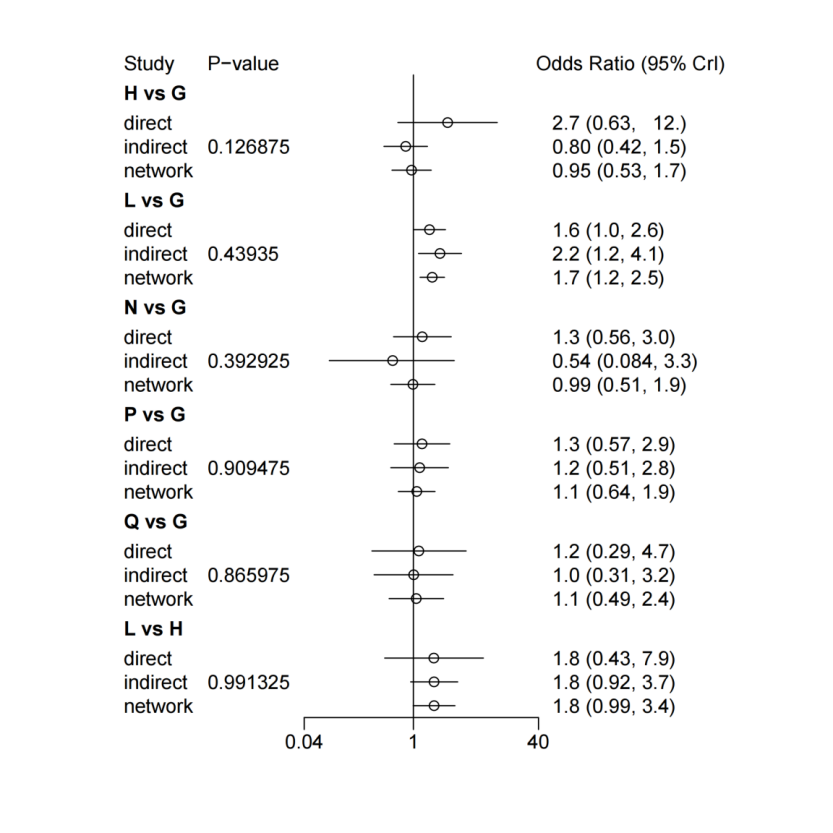

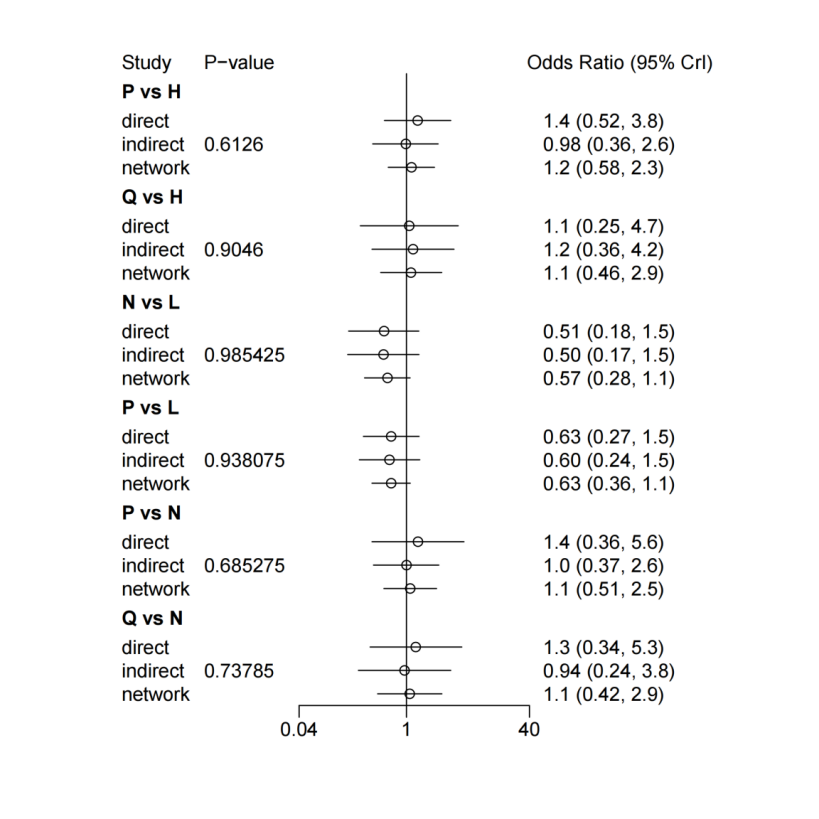


Note: A: instructGPT; B: GTP-3; C: ChatGPT-3.5; D: ChatGPT-4; E: ChatGPT-4o; F: ChatGPT-4V; G: Bard; H: PaLM2; I: Gemini; J: Gemini Advanced; K: Gemini 1.5 pro; L: Bing chat; M: Copilot; N: Perplexity; O: Perplexity Pro; P: Claude; Q: Claude-instant; R: Claude 2; S: Claude 2.1; T: Claude 3 Opus; U: Claude 3 Sonnet; V: Claude 3.5 Sonnet; W: LLaMA 7B; X: LLaMA 13B; Y: LLaMA 33B; Z: LLaMA 65B; A1: LLaMA 2; B1: LLaMA 3; C1: Mixtral-8x7B; D1: Mistral Large; E1: people; F1: chatENT; G1: ChatSonic; H1: Aeyeconsult; I1: Med-PaLM 2; J1: OcularBERT; K1: Doctor GPT; L1: Docs-GPT Beta; M1: WebMD; N1: Ada Health

There was no evidence of statistically significant inconsistency (*P* all >.05) in the node splitting test for NMA, except for the Claude 2 vs ChatGPT-4 (*P* =.04)、Bing chat vs people (*P* =.004) and Perplexity vs people (*P* =.04).

**Multimedia Appendix 8** Node splitting inconsistency test for open-ended questions


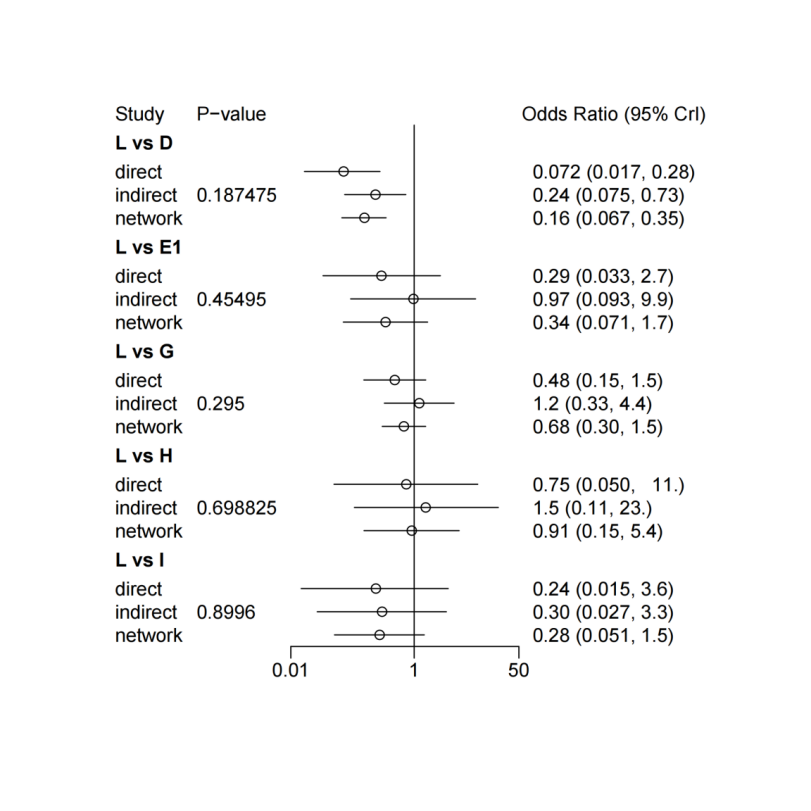

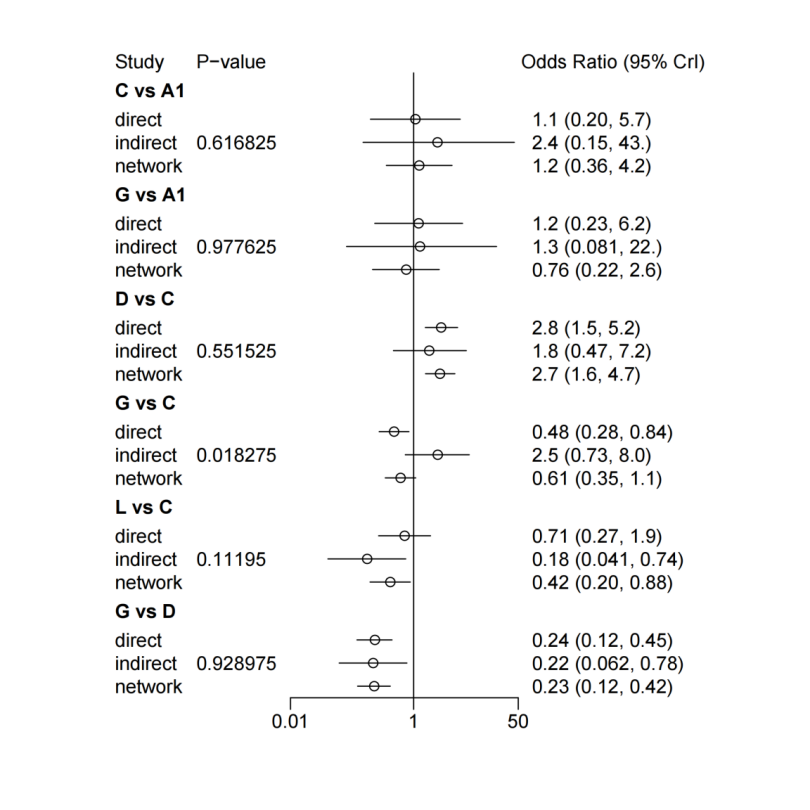


Note: C: ChatGPT-3.5; D: ChatGPT-4; E: ChatGPT-4o; G: Bard; H: PaLM2; I: Gemini; L: Bing chat; N: Perplexity; P: Claude; Q: Claude-instant; R: Claude 2; A1: LLaMA 2; E1: people

There was no evidence of statistically significant inconsistency (*P* all >.05) in the node splitting test for NMA, except for the Claude 2 vs ChatGPT-4 (*P* =.04)、Bing chat vs people (*P* =.004) and Perplexity vs people (*P* =.04).

**Multimedia Appendix 8** Node splitting inconsistency test for top 1 diagnosis


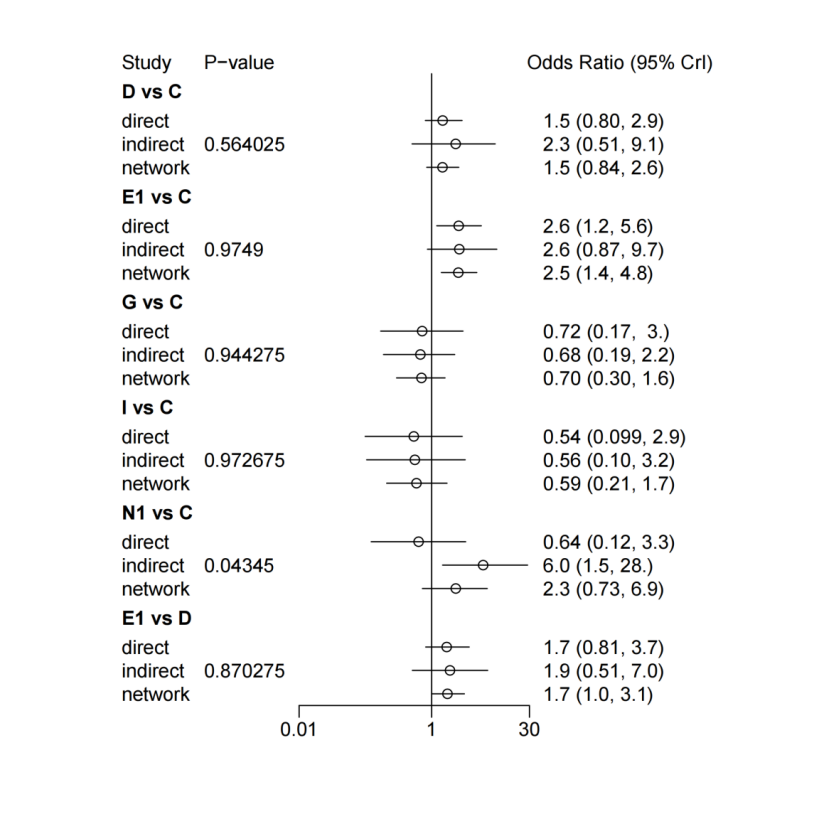

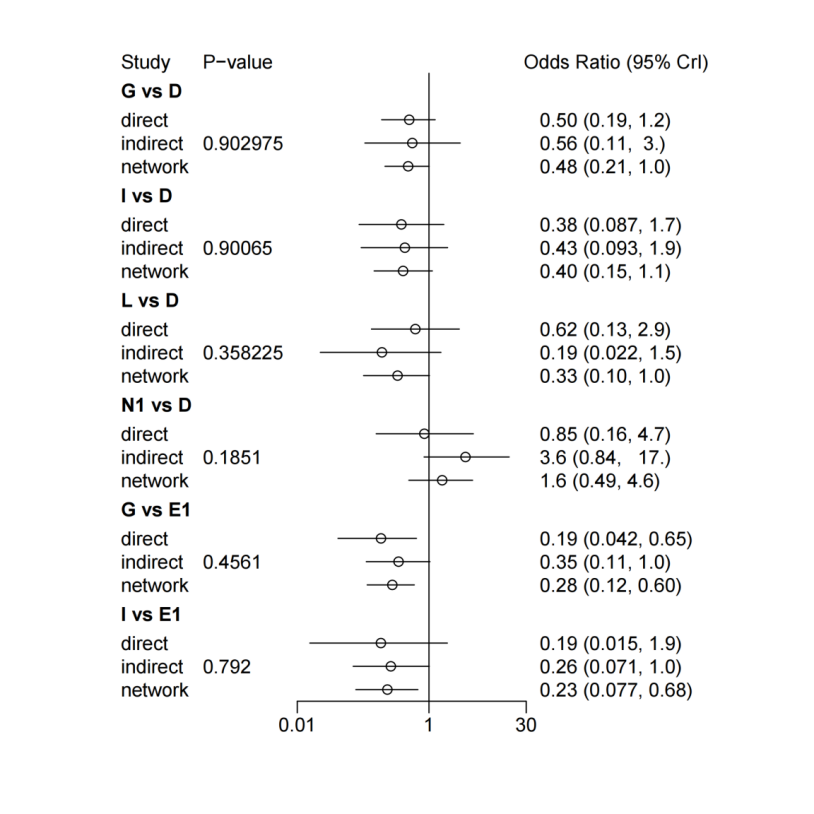


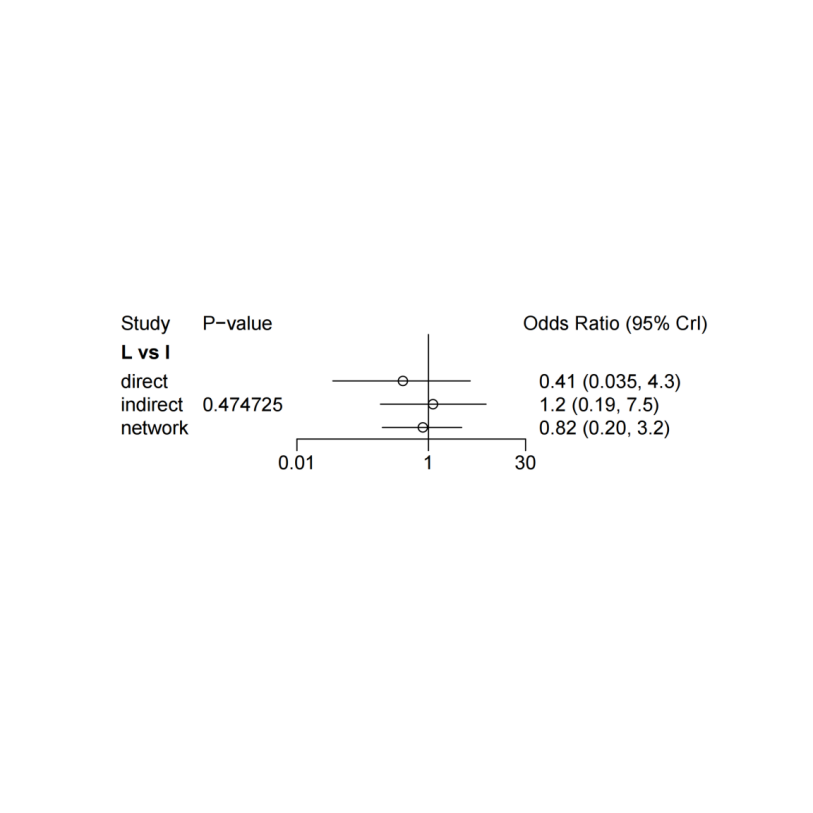


Note: C: ChatGPT-3.5; D: ChatGPT-4; G: Bard; I: Gemini; L: Bing chat; E1: people; N1: Ada Health

There was no evidence of statistically significant inconsistency (*P* all >.05) in the node splitting test for NMA, except for the Ada Health vs ChatGPT-3.5 (*P* =.043).

**Multimedia Appendix 8** Node splitting inconsistency test for top 3 diagnosis


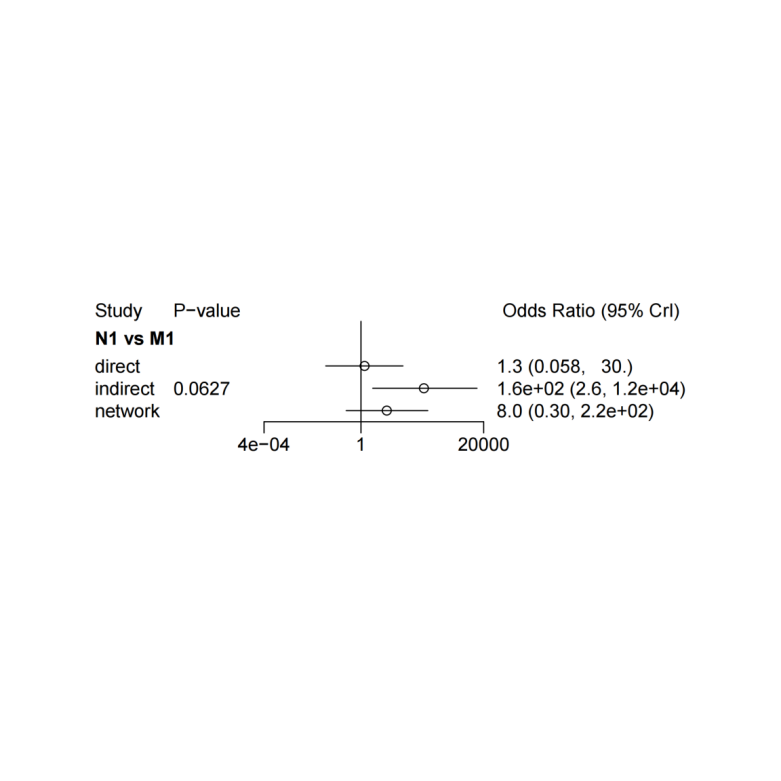

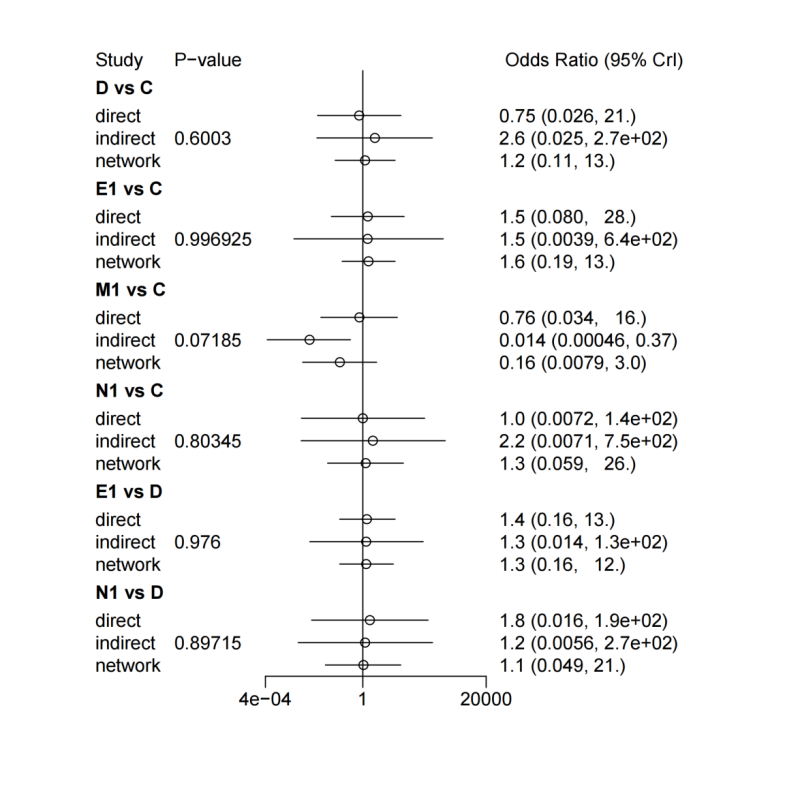


Note: C: ChatGPT-3.5; D: ChatGPT-4; E1: people; M1: WebMD; N1: Ada Health

There was no evidence of statistically significant inconsistency (*P* all > .05) in the node splitting test for NMA.

**Multimedia Appendix 8** Node splitting inconsistency test for top 5 diagnosis


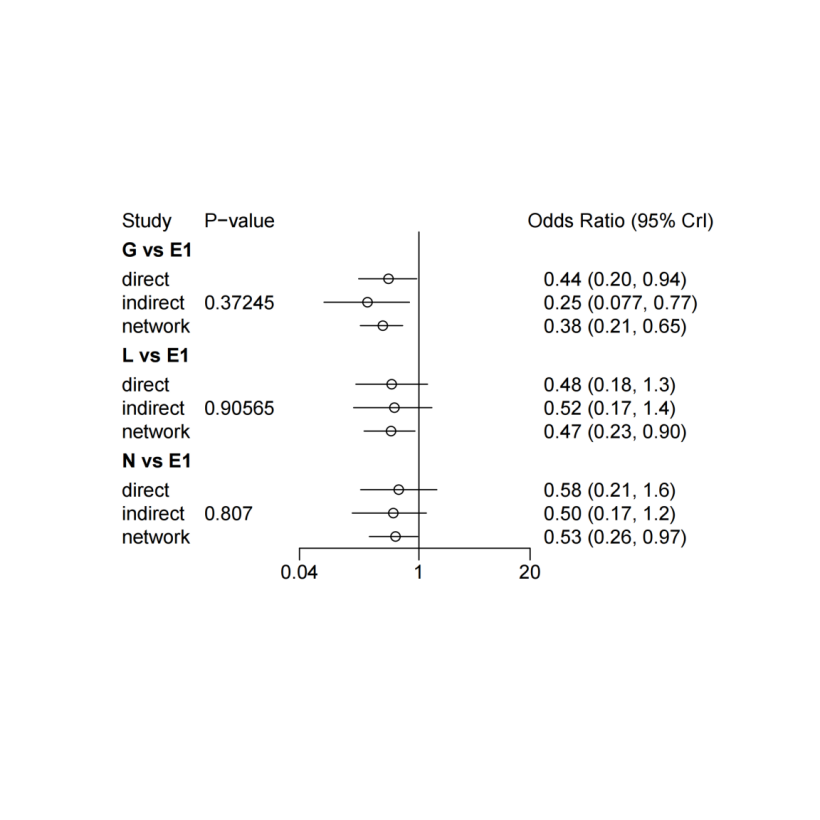

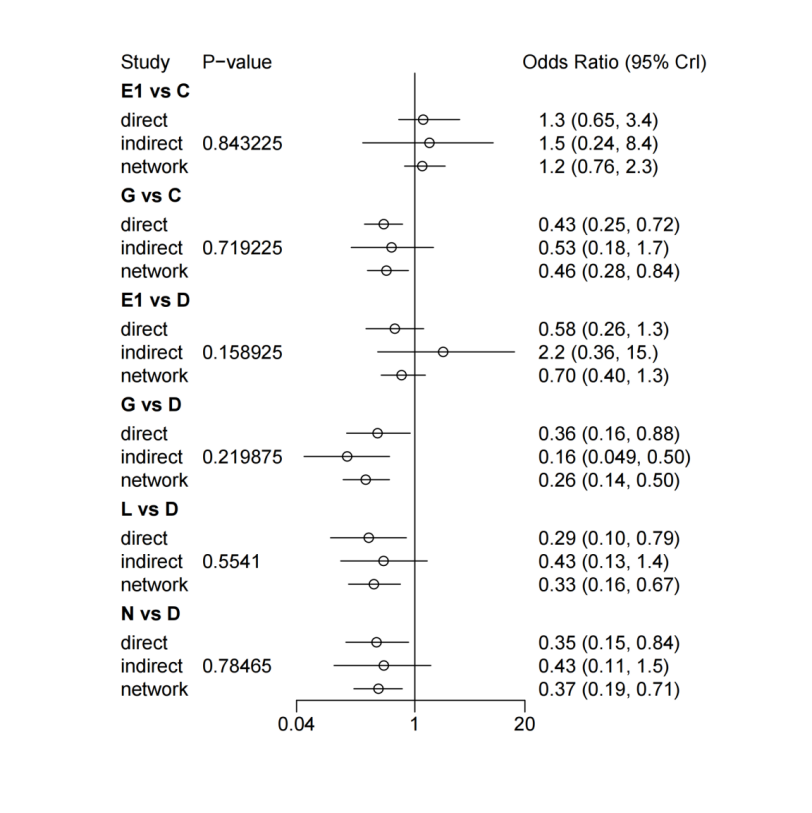


Note: C: ChatGPT-3.5; D: ChatGPT-4; G: Bard; L: Bing chat; N: Perplexity; E1: people

There was no evidence of statistically significant inconsistency (*P* all > .05) in the node splitting test for NMA.

**Multimedia Appendix
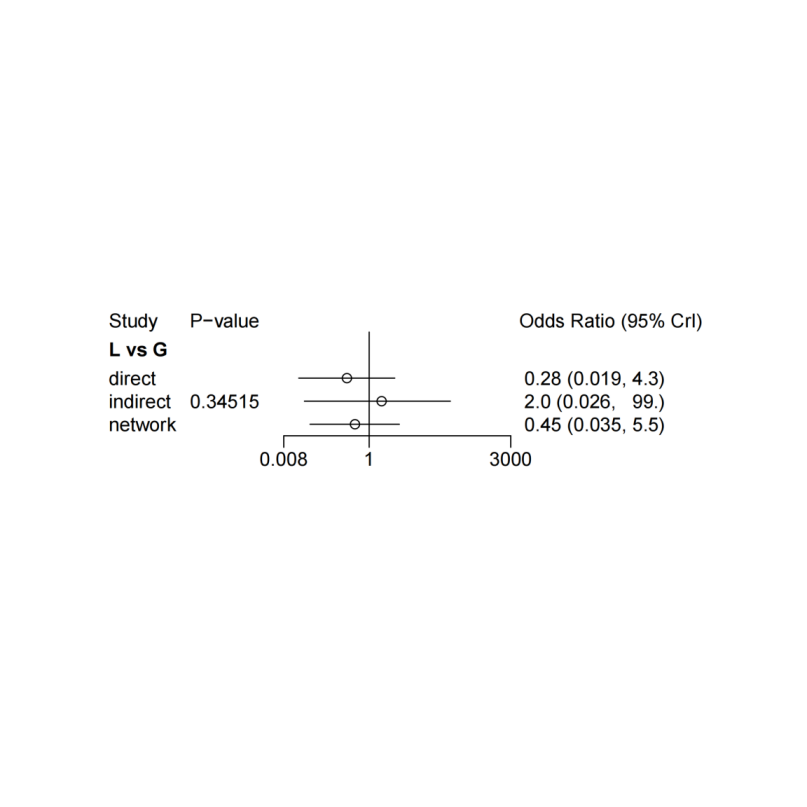
8** Node splitting inconsistency test for triage and classification


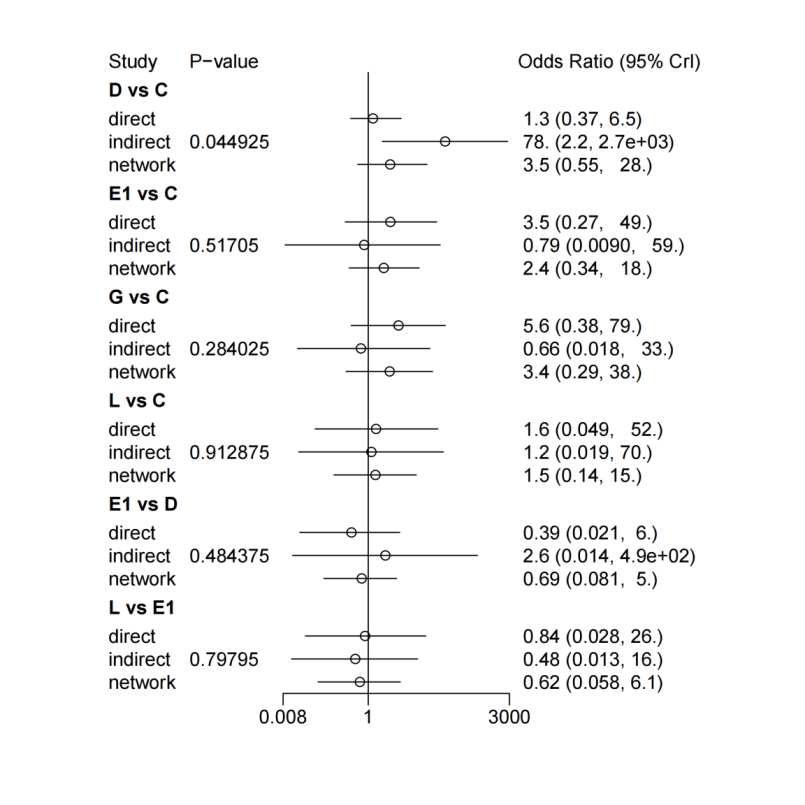


Note: C: ChatGPT-3.5; D: ChatGPT-4; G: Bard; L: Bing chat; E1: people

There was no evidence of statistically significant inconsistency (*P* all > .05) in the node splitting test for NMA, except for the chatGPT-4 vs ChatGPT-3.5 (*P* = .045).
