# Supplementary material for: Accuracy of Large Language Models When Answering Clinical Research Questions: Systematic Review and Network Meta-Analysis
Source: J Med Internet Res. 2025 Apr 30;27:e64486. doi: 10.2196/64486 (PMC12079073; doi:10.2196/64486)
Supplement: Multimedia Appendix 10 [file jmir_v27i1e64486_app10.docx]

**Multimedia Appendix 10** Objective questions are stratified according to different fields of the questions

| Field | Number of studies |
| --- | --- |
| ophthalmology | 9 |
| orthopedics | 9 |
| urology | 6 |
| dentistry | 4 |
| oncology | 4 |
| radiology | 6 |
| medical physics | 1 |
| medical physiology | 1 |
| Virology | 1 |
| gastroenterology | 2 |
| dermatology | 3 |
| cardiology | 3 |
| bariatric surgery | 1 |
| histology and embryology | 1 |
| Plastic Surgery | 1 |
| sleep medicine | 1 |
| Chinese medicine | 1 |
| anesthesiology | 1 |
| Neurology | 2 |
| otolaryngology | 2 |
| pediatrics | 2 |
| surgery | 2 |
| nephrology | 3 |
| nursing | 1 |
| Childcare | 1 |
| emergency medicine | 1 |
| veterinary surgeon | 1 |
| clinical pharmacy practice | 1 |
| breast cancer | 1 |
| obstetrics and gynecology | 1 |
| psychiatry | 1 |
| thoracic | 1 |
| comprehensive | 30 |
